# Natural Variation in Fish Transcriptomes: Comparative Analysis of the Fathead Minnow (*Pimephales promelas*) and Zebrafish (*Danio rerio*)

**DOI:** 10.1371/journal.pone.0114178

**Published:** 2014-12-10

**Authors:** Rong-Lin Wang, David C. Bencic, Natàlia Garcia-Reyero, Edward J. Perkins, Daniel L. Villeneuve, Gerald T. Ankley, Adam D. Biales

**Affiliations:** 1 Ecological Exposure Research Division, National Exposure Research Laboratory, US Environmental Protection Agency, Cincinnati, Ohio, United States of America; 2 Institute for Genomics, Biocomputing and Biotechnology, Mississippi State University, Starkville, Mississippi, United States of America; 3 Environmental Laboratory, US Army Engineer Research and Development Center, US Army Corps of Engineers, Vicksburg, Mississippi, United States of America; 4 Mid-Continent Ecology Division, National Health and Environmental Effects Research Laboratory, US Environmental Protection Agency, Duluth, Minnesota, United States of America; NIEHS/NIH, United States of America

## Abstract

Fathead minnow and zebrafish are among the most intensively studied fish species in environmental toxicogenomics. To aid the assessment and interpretation of subtle transcriptomic effects from treatment conditions of interest, better characterization and understanding are needed for natural variation in gene expression among fish individuals from lab cultures. Leveraging the transcriptomics data from a number of our toxicogenomics studies conducted over the years, we conducted a meta-analysis of nearly 600 microarrays generated from the ovary tissue of untreated, reproductively mature fathead minnow and zebrafish samples. As expected, there was considerable batch-to-batch transcriptomic variation; this “batch-effect” appeared to differentially impact subsets of fish transcriptomes in a nonsystematic way. Temporally more closely spaced batches tended to share a greater transcriptomic similarity among one another. The overall level of within-batch variation was quite low in fish ovary tissue, making it a suitable system for studying chemical stressors with subtle biological effects. The observed differences in the within-batch variability of gene expression, at the levels of both individual genes and pathways, were probably both technical and biological. This suggests that biological interpretation and prioritization of genes and pathways targeted by experimental conditions should take into account both their intrinsic variability and the size of induced transcriptional changes. There was significant conservation of both the genomes and transcriptomes between fathead minnow and zebrafish. The high degree of conservation offers promising opportunities in not only studying fish molecular responses to environmental stressors by a comparative biology approach, but also effective sharing of a large amount of existing public transcriptomics data for developing toxicogenomics applications.

## Introduction

Among an organism’s responses to environmental perturbations, gene transcription could be regarded as one of the earliest molecular events along the genotype-phenotype continuum. Dynamic and transient in nature for most genes, variation in this transcriptional response and its complex regulatory mechanisms is believed to contribute to much of the phenotypic complexity across biota [1], [2]. Whole genome expression profiling, now almost two decades old, along with other companion technologies, has fundamentally shifted the paradigms in biological research. Simultaneous determination of a large number of gene transcripts enables experimental and computational construction of a multitude of molecular interactions at the levels of both individual genes and biological networks/pathways, greatly facilitating the dissection of an organism’s responses to changes in its environmental conditions.

An organism’s transcriptome represents its full range of expressed gene transcripts regulated by a wide variety of molecular control mechanisms. At the molecular level, gene regulatory complexity is determined in part by the combinatorial nature of multiple cis-regulatory elements and trans-acting transcription factors [1]. Gene transcription is also a somewhat stochastic process [3], [4], with a certain degree of intrinsic “noise” [5]. Organisms with diverse genotypes are known to impact transcriptomes as well, probably through rewired gene regulatory circuits since regulatory polymorphisms are both cis- and trans-acting [6]. From a pragmatic perspective, transcriptome profiling could also be potentially complicated by the fact that, like many other fields of research, the work often has to be conducted in different phases over a period of time, often by different laboratories, and almost always using different materials (e.g., biological samples and reagents). Under these circumstances, transcriptomic data tend to be “batch-specific”. In this context, a batch can be defined by any one of several factors intrinsic to a study, for example, individual experiments, dates samples are treated, collected, or processed, and personnel involved in the lab work. Samples within a batch generally have a greater similarity to one another in their gene expression profiles than those between, a phenomenon commonly referred as “batch effects” [7]. Since these batch factors are not necessarily independent, between-batch variation may reflect some of the interactive effects of these variables as well. While batch effects themselves are typically not the intended targets of a scientific investigation, their correlations with treatment conditions need to be taken into account statistically during data analysis to avoid making erroneous conclusions [8]–[10].

It is, of course, also possible to introduce technical variation into transcriptomic data due to methodological differences across studies, for example, in RNA sample preparation, choice of profiling platforms, data processing approaches, and so on. Over the years, there have been extensive investigations into these technical issues and their impact on gene expression profiling by microarrays [11], [12]. Typically in these studies, common RNA samples were distributed across labs and tested on various microarray platforms. Different normalization and analytical procedures were then applied to identify differentially expressed genes (DEGs) and the concordance among the resultant gene sets evaluated. It has been found that technical reproducibility across microarray platforms and labs is generally high [11], and that fold-change in gene expression is the most consistent metric for comparisons between microarrays and qPCR (quantitative polymerase chain reaction) [13], [14]. While generally giving similar results, optimum normalization methods seem to be data-dependent [15]. Variability observed among microarray platforms could largely be attributed to differences in probe sequences and reduced sensitivity in detecting more weakly expressed genes [14]. Variability among microarray runs is also typically low. Overall, technical variability beyond between-batch variation appears to be of relatively minor importance in microarrays and the concordance is high across sites, platforms, and data analysis approaches.

Among a wide range of applications of transcriptomics in various disciplines are human disease diagnostics/prognostics [16] as well as chemical toxicity and exposure assessment in ecotoxicology [17]. Yet, in spite of years of developmental effort, consistency and predictability are still quite low in many gene expression-based disease biomarkers [18], [19], nor is there a significant number of ecotoxicological biomarkers proved to be field-ready. One of the major contributing factors behind these underperforming biomarkers is probably our inadequate knowledge of the extent and scope of the variability in their respective transcriptomes, particularly that of biological nature. A lack of thorough characterization and understanding of such variability makes it difficult to optimize the designs of transcriptomics experiments, has an adverse impact on the delineation of molecular mechanisms of action for hazardous chemicals, and impedes the development of their molecular biomarkers effective on samples independently collected under different conditions over time.

Fish in general are among the most commonly studied non-mammalian organisms in environmental toxicogenomics, with the fathead minnow and zebrafish (*Danio rerio*) arguably the most studied species in this diverse group. As a common biological model with extensive genome-level knowledge, the zebrafish is a logical species of choice for toxicogenomics work. The fathead minnow also is an attractive model species because it has been the dominant aquatic vertebrate test organism in regulatory toxicity testing for decades [20]. The relatively recent evolutionary divergence between the two species also means that a substantial amount of biological information is transferable between them. There are abundant and growing “-omics” resources available for both species. Nonetheless, despite numerous transcriptomic studies focused on molecular mechanisms of action of chemicals in the context of hazard identification and biomarker development, relatively little attention has been paid to transcriptomic variation among a common batch of untreated individuals from lab cultures. While well studied in other species, between-batch variation in these fish, to our knowledge, has not been characterized either. In a recent report on fathead minnow (*Pimephales promelas;* PPR), based on samples across different gender-tissue-treatment conditions, variability among individuals was found to be very high, with a wide-ranging distribution among genes as well as molecular networks [21].

An assessment and basic understanding of the extent and scope of such variations should be considered an important priority in order to improve interpretation of treatment effects caused by chemical stressors and their discrimination from background variability. Our research team has conducted a number of microarray studies focused on chemical effects on reproductive pathways in zebrafish and fathead minnows [22]. This dataset provides an opportunity to evaluate issues related to transcriptome variability. Given the strong impact of gender and tissue type on gene expression profiles from these studies [23], the “baseline” analysis described herein was restricted to ovary samples from untreated, reproductively mature fathead minnow and zebrafish in order to avoid contributions from sex- and tissue-dependent effects. The objectives of the current analyses were to determine: 1) the extent of between-batch variation, namely, how much gene expression profiles of untreated lab fish change over time or experiments; 2) the extent of within-batch variation in gene expression among individuals and its partitioning across different levels of organization, from whole transcriptome, to molecular pathways and individual genes; and 3) transcriptomic conservation between fathead minnow and zebrafish with respect to their within-batch variation.

## Materials and Methods

A total of 511 fathead minnow and 80 zebrafish microarrays from samples of ovary tissue in untreated lab fish were assembled from a number of experiments conducted by our research team between 2004 and 2010 (Table 1). These microarray samples could be grouped in a variety of ways. For the purposes of this investigation, we chose to consider five factors that broadly align with various stages of gene expression profiling: the original experiments they belonged to (Experiment), dates the ovary tissue samples were collected (Sampling Date), dates RNA samples were prepared (RNA Date), people who prepared RNA (RNA Person), and the date microarrays were scanned (Scan Date). These factors were nested to various degrees so they were not independent. Each factor contained a different number of batches (i.e., different experiments, sampling dates, or people). Throughout this report, between-batch variation is defined as significant changes in gene expression between two batches of untreated samples within the same factor and measured by the number of DEGs. Within-batch variation is defined as differences in gene expression among individuals within the same batch after taking into account between-batch variation statistically. Note that standard normalization methods cannot effectively remove between-batch variation because of its nonsystematic and differential impact on subsets of a transcriptome [8].

**Table 1:** Factors and batches (N) by which fathead minnow (PPR) and zebrafish (DRE) microarray samples were organized.

Species	Experiment	Sampling Date	RNA Date	RNA Person	Scan Date
	N = 9	N = 9	N = 4	N = 2	N = 5
DRE (80)	CTL_FAD24 (5)	CTL_2004_12 (10)	CTL_2005_1 (10)	CTL_A (65)	CTL_2007_12 (47)
	CTL_FIP48_96 (10)	CTL_2005_1 (5)	CTL_2005_3 (5)	CTL_B (15)	CTL_2007_2 (4)
	CTL_FLU48_96 (10)	CTL_2006_10 (10)	CTL_2006_11 (30)		CTL_2007_3 (24)
	CTL_KTC24_48_96 (15)	CTL_2006_4 (10)	CTL_2007_6 (35)		CTL_2007_4 (2)
	CTL_MUSC96 (5)	CTL_2006_5 (10)			CTL_2008_4 (3)
	CTL_PRO48_96 (10)	CTL_2006_9 (10)			
	CTL_TRB24_48 (10)	CTL_2007_2 (5)			
	CTL_TRI96 (5)	CTL_2007_4 (5)			
	CTL_VIN48_96 (10)	CTL_2007_5 (15)			
	N = 23	N = 16	N = 17	N = 10	N = 18
PPR (511)	BPA_NOTEL (6)	2007_12 (4)	11111 (12)	DDD (20)	2008_10 (10)
	FAD_I_Acute (12)	2007_2 (12)	2007_12 (58)	A (32)	2008_11 (22)
	FAD_III_Acute (20)	2007_3 (72)	2007_4 (12)	B (124)	2008_12 (62)
	FAD_Phase3 (60)	2007_6 (54)	2007_6 (60)	B_ROBOT (54)	2008_4 (12)
	FLU_II_Acute (20)	2007_7 (24)	2008_11 (19)	C (24)	2008_5 (10)
	FLU_Phase3 (38)	2008_1 (54)	2008_2 (12)	J (23)	2008_6 (132)
	GEM (5)	2008_2 (12)	2008_6 (124)	L (126)	2008_7 (38)
	KTC_I_Acute (12)	2008_4 (51)	2008_7 (44)	R (60)	2008_8 (26)
	KTC_IV_Acute (28)	2008_5 (78)	2008_9 (32)	X (36)	2009_1 (40)
	KTCv2_Phase3 (39)	2008_8 (52)	2009_1 (20)	YYY (12)	2009_10 (6)
	PRO_I_Acute (12)	2009_1 (5)	2009_10 (44)		2009_11 (39)
	PRO_Phase3 (54)	2009_10 (67)	2009_11 (29)		2009_2 (30)
	RDX_Repro (4)	2009_6 (4)	2009_7 (9)		2009_6 (23)
	TNT_KTC_Acute (24)	2009_7 (10)	2009_9 (5)		2009_7 (8)
	TRB_BPA (6)	2009_9 (6)	2010_02 (6)		2009_9 (14)
	TRB_EE2 (4)	2010_1 (6)	2010_1 (5)		2010_1 (28)
	TRB_Phase3 (54)		55555 (20)		2010_2 (5)
	TRB_TCC (5)				2010_7 (6)
	TRI_II_Acute (20)				
	TRI_Phase3 (32)				
	VIN_II_Acute (19)				
	VIN_Phase3 (32)				
	WLSSD (5)				

Sample sizes were indicated in parenthesis.

BPA, bisphenol-A; EE2, 17a-ethynyl estradiol; FAD, fadrozole; FIP, fipronil; FLU, flutamide; GEM, Gemfibrozil; KTC, ketoconazole; PRO, prochloraz; RDX, hexahydro-1,3,5-trinitro-1,3,5-triazine; TRB, 17 -trenbolone; TNT, 2,4,6-trinitrotoluene; TRI, trilostane; VIN, vinclozolin; WLSSD, effluent from Western Lake Superior Sanitary District; RNA Date not determined: 11111, 55555; RNA Person not determined: DDD, YYY.

### Fish culture, sampling, RNA preparation, and microarray profiling

A brief overview of fish culture, sampling, RNA preparation, and microarray gene expression profiling is provided below. Further details about these procedures were described elsewhere [22]–[24]. Because these samples originally served as controls for treatments with a variety of chemicals, this overview describes common procedures applied to both treated and untreated fish from their respective experiments. Moreover, these experiments were conducted over a period of six years, thus can be considered largely independent. All animals were treated humanely and with regard for alleviation of suffering, and all laboratory procedures involving animals were reviewed and approved by the US EPA Animal Care and Use Committee in accordance with Animal Welfare Act regulations and Interagency Research Animal Committee guidelines. The entire microarray dataset is available at the National Center for Biotechnology Information Gene Expression Omnibus (NCBI-GEO) [25] as the accession GSE60202.

#### Zebrafish Experiments

Reproductively mature zebrafish (ab wild-type strain, 5–7 months old) were exposed to a continuous flow of sand filtered, UV-sterilized, Lake Superior water (LSW; controls) for 24, 48, or 96 h at the US Environmental Protection Agency (USEPA) laboratory in Duluth, Minnesota. At the end of each exposure period, fish were anesthetized in a buffered solution of tricaine methanesulfonate (MS-222; Finquel, Argent, Redmond WA, USA) and ovaries were collected and shipped overnight on dry ice to the USEPA laboratory in Cincinnati, Ohio. Total RNA isolated from selected tissue samples was then sent to Cogenics Corporation, an Agilent certified contract laboratory (Morrisville, North Carolina 27560, USA). Hybridization was conducted using a two-color protocol on Agilent zebrafish microarrays with 21K probes (design 013223 and design 015064, Agilent Technologies, Santa Clara, CA, USA), followed by high-resolution scanning and image processing by Agilent Feature Extraction software. Eighty ovary controls for nine treatment conditions, all based on the design 015064, were included in this study.

#### Fathead Minnow Experiments

Reproductively mature fathead minnows (5–7 months old) reared at the US EPA laboratory in Duluth were exposed to LSW. All exposures were continuous flow-through exposures. Representative experimental designs for the experiments included as part of the current analysis have been detailed elsewhere [26], [27]. At the end of each exposure period, ovary tissues used for the transcriptomic analyses considered in the present study were snap-frozen in liquid nitrogen and stored at −80°C until RNA was extracted, using either Qiagen RNeasy mini kits (Qiagen, Valencia, CA, USA) or Tri-Reagent (Sigma, St. Louis, MO, USA). Expression profiling was carried out using a single-color protocol on Agilent fathead minnow microarray with 15 K probes (design 019597, GEO accession GPL9248) [28] at the Environmental Laboratory of the US Army Engineer Research and Development Center in Vicksburg, Mississippi. One µg of total RNA was used for all hybridizations. Probe labeling, amplification, and hybridization were performed using Agilent Quick Amp Labeling Kit following the manufacturer’s One-Color Microarray Hybridization Protocol. Microarrays were scanned with a high-resolution scanner and the images were processed with Agilent Feature Extraction software.

### Microarray Data Analysis

Various analyses were conducted in the R environment (www.r-project.org) primarily by using the Limma package [29]. Pre-processing was first conducted on the raw data files from Agilent Feature Extraction software prior to any analysis. This included background correction and quantile normalization for single-color fathead minnow microarrays, and background correction, loess normalization, and quantile normalization for two-color zebrafish microarrays.

#### Between-batch Variation

The size of between-batch effect could be estimated by DEG count, variance partitioning of total variance on a per gene basis to within-batch and between-batch components, or a composite variance measure such as that from principal variance component analysis, a hybrid of principle component analysis (PCA) and variance partitioning [7]. To be comparable, however, to the typical estimates of treatment effects in transcriptomics as well as the estimates of transcriptomic variation both within and between populations [30]–[36], DEG count was chosen in this study to approximate the scale of between-batch variation. Since many combinations of factor by batch were not available in this leveraged dataset, factorial analysis was not possible. Some of the combinations also lacked adequate replication. Hence, between-batch variation in gene expression was determined by forming statistical contrasts among various batches under individual factors and then identifying the number of DEGs therein by modified t-tests. Samples from individual batches were compared to two different types of references so the between-batch variation for a given factor could have two estimates for comparison. The first was constructed by taking the mean expression value of each gene among the replicates within each batch under a factor. In other words, a factor with four batches each containing multiple replicates would form a simulated reference group containing four expression values for each gene. This method will hereafter be referred to as the simulated reference. The second reference type was used in conjunction with estimating within-batch variation, where an original factor (for example Experiment) and a simulated factor (containing two created levels of “case” and “control”) were included in a general linear model (GLM). The batches under the original factor effectively served as experimental blocks. To estimate between-batch variation, one batch was designated as a common reference and compared against each of the remaining batches throughout the permutations. This method will be referred to as the designated reference.

Between-batch variation was further evaluated by several approaches. To provide an empirical critical value to assess the statistical significance of DEG counts, a non-parametric distribution of the number of DEGs between batches was generated. Microarray sample labels were permuted according to the original number of replicates in each batch under the factor Experiment to generate 1000 simulated datasets, followed by identifying DEGs in paired comparisons between a selected common reference batch and each of the remaining batches in every dataset. Note that a different batch was selected as a common reference in the analysis of each simulated dataset. To determine if between-batch variation is biologically significant, the 100 DEGs with the highest F statistic p-values for between-batch variation under each factor were also combined and analyzed for possible enrichment in biological pathways by DAVID (The Database for Annotation, Visualization and Integrated Discovery) [37]. Finally, to visualize batch effects, all DEGs identified in each factor were combined and made non-redundant. PCAs were conducted on these DEGs based on the expression values of either individual samples or the average of individual batch in each factor. Similarly, hierarchical clustering was carried out on DEGs using the R package PVclust with 100 bootstraps (http://www.is.titech.ac.jp/~shimo/prog/pvclust/).

#### Within-batch Variation

Individuals within a batch are subjected to the same degree of technical variation, thus their differences should better reflect biological variability. We used two different approaches to evaluate within-batch variation. First, for a given factor, half the samples in each batch randomly assigned as “case” were compared to the remaining half assigned as “control” for DEGs during each of 250 permutations. Between-batch variation was controlled in these analyses by including the factor under consideration in the GLM to ensure the comparisons were made within a batch. This method essentially searched through samples’ possible membership assignments between the two classes to uncover the configuration where the number of DEGs was maximized. This DEG count served as an indirect measure of the variation among individuals. To be included in these permutations, each batch needed to contain a minimum of ten (zebrafish) or 12 (fathead minnow) samples, respectively, to ensure that there were at least 250 unique permutations and the maximum amount of data was utilized. Second, the coefficient of variation (CV) and intensity of individual probes were also calculated from multiple biological replicates in individual batches under the Experiment factor and averaged over all its batches. An assessment of these two simple metrics both within and between the two species, at the levels of entire transcriptome, KEGG (Kyoto Encyclopedia of Genes and Genomes) molecular pathways, and individual genes, would be informative of not only gene expression variability among individuals and relative biological contribution to this variability, but also the interspecific transcriptomic conservation as well. The simple linear regressions and validations of their underlying statistical assumptions were conducted for selected inter- and intraspecific comparisons of these two metrics using “RegressIt”, an Excel macro developed jointly at Duke University and University of Texas (http://regressit.com/index.html).

To allow interspecific comparison of within-batch variation, probes from Agilent design 019597 (fathead minnow) and 015064 (zebrafish) representing orthologous genes were identified through several successive steps of identification (ID) mapping. Probe sequences from Agilent 019597 were first mapped to their corresponding EST (Expressed Sequence Tag) target sequences (courtesy of Dr. Nancy Denslow, University of Florida, Gainesville, Florida, USA; ndenslow@ufl.edu) by TBLASTX so the latter with a greater sequence length could be used as queries for more successful mapping across species. The EST sequences were then mapped to the NCBI (National Center for Biotechnology Information) nucleotide (NT, as of March, 2013) and protein (NR, as of July 2013) databases by TBLASTX and BLASTX respectively, effectively associating fathead minnow probe IDs to their corresponding NCBI accession IDs. All three rounds of mapping had a minimum E-value cutoff of E^−06^. These fathead minnow IDs were then joined to a variety of zebrafish accession IDs prepared by the NCBI (“gene2accession” as of April, 2014; ftp://ftp.ncbi.nlm.nih.gov/gene/DATA/), and finally to Agilent probe annotations (https://earray.chem.agilent.com/earray/). In the end, a total of 9311 probes from probes from Agilent 019597 through 6617 common Entrez GeneIDs in NCBI (S1 Table). These probes were then organized into 162 KEGG pathways available as of April, 2014 (http://rest.kegg.jp/link/dre/pathway) by these GeneIDs. Before the pathway level the CV and intensity of those duplicated probes were consolidated first by Entrez GeneIDs.

The analytical procedures described above for both between- and within-batch variation were incorporated into several R (www.r-project.org) scripts developed by the authors based primarily on the R. In contrast to DEG determination by a regular t-test (or F-test), this software fits expression data of each gene into a linear model and generates a modified t-statistic (or F-statistic for multiple contrasts) using an empirical Bayesian and hierarchical modeling approach to adjust for unreliable variance estimates caused by small sample size [38]. The work flow started with data pre-processing, followed by linear model fitting, calculating a modified t-statistic for each gene, and finally multiple test corrections to generate DEGs from a given statistical contrast. Since the fathead minnow and zebrafish data were in Agilent one-color and two-color formats respectively, they were handled differently in normalization. The fathead minnow data were quantile-normalized only. The zebrafish data were, however, both loess- [39] and quantile-normalized. These within- and between-array normalization were necessary for zebrafish microarrays because their two-color channels each containing a treated and a control sample had to be split into single channel intensities; and only the control samples were analyzed across microarrays [29]. The channel-splitting was accomplished by several relevant functions in the Limma package based on mixed model methods to effectively decouple the correlated intensities between the two channels.

## Results

### Between-batch Variation

Between-batch variation was estimated by organizing samples according to one of the five factors under consideration and then comparing each batch to either the simulated reference or the designated reference. Both types of comparisons revealed considerable variation between batches in fathead minnow and zebrafish (Table 2, 3). Measured against the threshold values of 104 and 594 at 0.1% significance from the non-parametric distributions generated for fathead minnow and zebrafish by permutations (Figure S1A, S1B in S1 File), the average numbers of DEGs per pair of batch comparison were all statistically significant, regardless of the species and factors under which batches were compared. In general, between-batch variation measured by the simulated reference method was less than that of the designated reference, especially in fathead minnow. When all the DEGs from between-batch variation were pooled under individual factors, the total number (percentage of total genes) impacted were 13101 (86%; Experiment), 10871 (71%; RNA Date), 10576 (70%; Sampling Date), 10160 (67%; Scan Date), and 3322 (22%; RNA Person) for fathead minnow, and 13581 (63%; Experiment), 12522 (58%; RNA Date), 13581 (63%; Sampling Date), 10187 (47%; Scan Date), 9383 (44%; RNA Person) for zebrafish.

**Table 2:** Between-batch variation as measured by average number of DEGs (standard deviations where N>3).

Species/sample condition	Experiment	Sampling Date	Scan Date	RNA Date	RNA Person
PPR	2360 (1033)	1498 (1032)	1123 (1102)	1907 (756)	434 (525)
DRE	2754 (1684)	2754 (1684)	2845 (2320)	4403 (2435)	5593

The DEGs were identified in paired comparisons of N batches against a simulated reference made up of the batch means of their respective factors. DRE, zebrafish; PPR, fathead minnow.

**Table 3:** Between-batch variation as measured by average number of DEGs (standard deviations where N>3).

Species/sample condition	Experiment	Sampling Date	Scan Date	RNA Date	RNA Person
PPR	4517 (889)	4312 (1009)	4640 (1616)	4667 (941)	5872 (2045)
DRE	2523 (1651)	2841 (2141)	6321	5503	3669

The DEGs were identified in paired comparisons of N–1 batches against a batch designated as a common reference. N is the total number of batches in a factor. The comparisons were made in conjunction with the analysis of within-batch effects involving 250 permutations. There were little variations in between-batch effects among permutations so their calculations were made only from the first permutation. DRE, zebrafish; PPR, fathead minnow.

Between-batch variation was also apparent in PCA plots (Figure S2–S6 in S2 File, Figure S7–S11 in S3 File) and dendrograms (Figure S12–S16 in S4 File, Figure S17–S21 in S5 File). For example, samples were largely segregated in the PCA plots by the batch they belonged to under the Experiment factor in both species (Figure S2B in S2 Files, Figure S7B in S3 File). There was also a tendency for batches that were temporally closer to one another to share a greater similarity in their gene expression profiles (Figure S5A in S2 File, Figure S10A in S3 File). The numerous fathead minnow samples made it difficult to observe sample clustering by batch in their crowded dendrogram (Figure S12B in S4 File), but a pattern could clearly be seen with the zebrafish samples (Figure S17B in S5 File). Indeed, the PCA plots and dendrograms showed very similar patterns of sample clustering by batch in each of the four other factors of both species.

The DEGs from between-batch variation in both the fathead minnow and zebrafish were not enriched with any KEGG pathways, GO terms, or other types of gene functional groups (S2 Table, S3).

### Within-batch Variation

**Figure 1:**
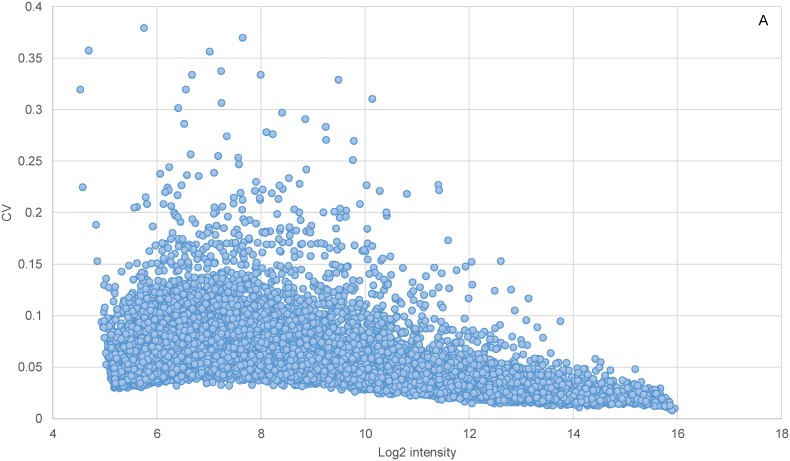
Estimation of within-batch variation. Coefficients of variation (CV) were computed at various intensities of 15208 fathead minnow probes (A) and 21495 zebrafish probes (B).

Within-batch variation was assessed based on DEG counts and gene expression CVs. Identified between two classes of “case” and “control” randomly created within each batch by many permutations, the maximum number of DEGs per permutation varied considerably among the factors. In the fathead minnow, RNA Person generated the greatest number of DEGs, followed by Sampling Date, Experiment, RNA Date, and Scan Date (Table 4). In zebrafish, within-batch variation was again the largest under RNA Person, followed by Scan Date, RNA Date, Experiment, and Sampling Date. When measured by the CVs of individual genes within each batch under the Experiment factor, the picture of within-batch variation for these species became quite complex. Globally, when all the genes from each species were considered, those with lower expression tended to have greater CVs in general (Fig. 1A, 1B). However, the genes with the highest variability appeared to have different levels of expression in the two species: lower level in the fathead minnow but more mid-level in zebrafish. With a global CV of around 0.05 as averaged across all genes over individual batches, approximately 50% of genes had CVs below and 30% of genes above this average, in both species (Table 5, Figure S22A and S22B in S6 File). There was little batch-to-batch difference in CVs averaged within individual batches. Roughly 5% of genes had CVs greater than 0.1. And similar percentages of genes were distributed over the entire range of CVs in the two transcriptomes. However, comparison of orthologs between the two species suggested that fathead minnow was somewhat more variable than zebrafish, as a greater percentage of genes of the former species were found in ranges with higher CVs.

**Table 4:** Within-batch variation as measured by the maximum (% of transcriptome) and minimum number of DEGs per permutation.

DEGs perpermutation	Experiment	Experiment	SamplingDate	SamplingDate	ScanDate	ScanDate	RNADate	RNADate	RNAPerson	RNAPerson
	PPR	DRE	PPR	DRE	PPR	DRE	PPR	DRE	PPR	DRE
Maximum	267 (1.8)	52 (0.2)	393 (2.6)	10 (0.05)	25 (0.2)	1120 (5.2)	156 (1.0)	192 (0.9)	883 (5.8)	3613 (16.8)
Minimum	0	0	0	0	0	216	0	0	0	1153

There were 250 permutations conducted under individual factors. Between-batch variation was controlled statistically in these analyses. PPR, fathead minnow; DRE, zebrafish.

**Table 5:** Within-batch variation as measured by coefficient of variation (CV).

CV range	DRE probes (% total)	PPR probes (% total)	DRE probes orthologous to PPR	PPR probes orthologous to DRE
<0.01	409 (1.9)	3 (0.02)	344 (3.7)	1 (0)
0.01–0.049	10721 (49.9)	7625 (50.1)	5956 (64.0)	3825 (55)
0.05–0.059	3651 (17.0)	2738 (18)	922 (9.9)	1166 (16.8)
0.06–0.069	2728 (12.7)	1661 (10.9)	738 (7.9)	662 (9.5)
0.07–0.079	1573 (7.3)	1065 (7.0)	440 (4.7)	429 (6.2)
0.08–0.089	856 (4.0)	664 (4.4)	276 (3.0)	278 (4.0)
0.09–0.099	491 (2.3)	466 (3.1)	168 (1.8)	183 (2.6)
0.1–0.199	896 (4.2)	909 (6.0)	406 (4.4)	379 (5.5)
≥0.2	170 (0.8)	77 (0.5)	61 (0.7)	27 (0.4)
Total	21495	15208	9311	6950

The CVs were calculated for each probe by individual batches under the Experiment factor and averaged over all batches. Further averaging these CVs across the entire transcriptome yielded an overall CV of 0.056 for fathead minnow (PPR) and 0.051 for zebrafish (DRE). The total number of orthologous genes identified between Agilent 015064 and 019597 was 6617, represented by 9311 and 6950 unique probes respectively. The PPR probes mapped to their EST sequences and ESTs to NCBI databases by BLAST all had a minimum E-value of E-06.

**Figure 2:**
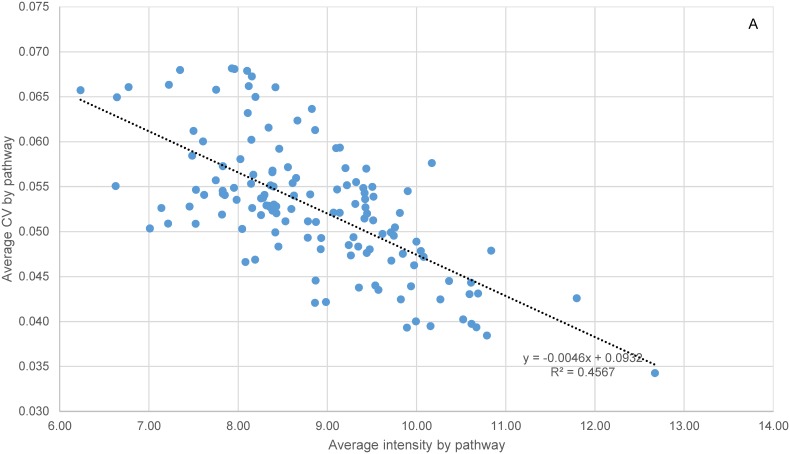
Intraspecific correlation between the average CV and average intensity by KEGG pathways. A total of 136 pathways (eight outliers excluded) were included for fathead minnow (A) and 144 pathways for zebrafish (B). The CCs were −0.68 and −0.70 respectively, with both p-values = 0. The p-values of normality test of error distribution for linear regressions were 0.094 (no significant departure from normality) and 0 (significant non-normality) respectively.

Within-batch variation was also examined within individual species at the molecular pathway level by placing the 6617 orthologs into individual KEGG pathways (S4 Table). There were 144 out of 162 pathways each containing at least five orthologs. Compared to the global transcriptomic CV average of 0.05 in both species, the average CV by pathways ranged from 0.018 (dre03430, Mismatch Repair) to 0.098 (dre00360, Phenylalanine Metabolism) in zebrafish, and 0.034 (dre03010, Ribosome) to 0.082 (dre00072, Synthesis and Degradation of Ketone Bodies) in fathead minnow. Globally, average gene expression intensity among the individuals within the same batch was 8.5 for the two species. At pathway level, gene expression intensity ranged from 6.33 (dre04080, Neuroactive ligand-receptor interaction) to 13.63 (dre03010, Ribosome) in zebrafish, and from 6.23 (dre00360, Phenylalanine Metabolism) to 12.67 (dre03010, Ribosome) in fathead minnow. In both species, the average CV by pathway was inversely correlated with the average expression intensity (Fig. 2A, 2B).

**Figure 3:**
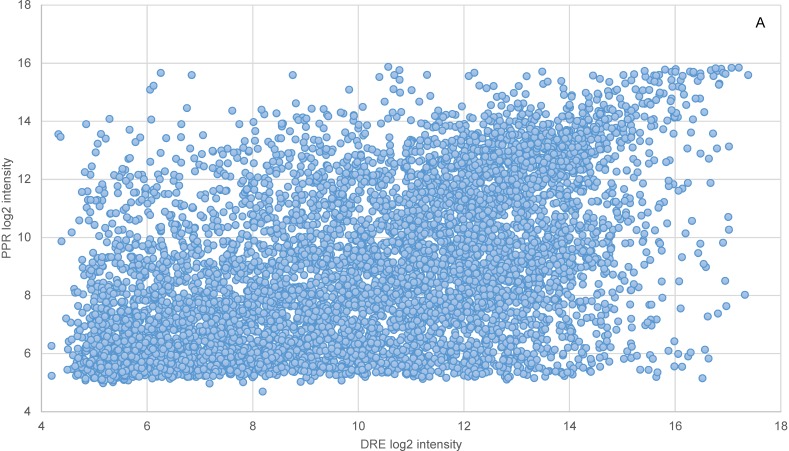
Interspecific correlation by within-batch intensity and coefficient of variation of orthologs. The within-batch intensities (A) and coefficients of variation (CV; B) were based on 6617 orthologous genes. The orthologs were represented by 9311 zebrafish (DRE) and 6950 fathead minnow (PPR) probes. The intensity and CV of an ortholog with duplicated probes were probe means. The correlation coefficients over the orthologs for the two metrics were 0.49 and 0.33 respectively, with the both p-values = 0.

**Figure 4:**
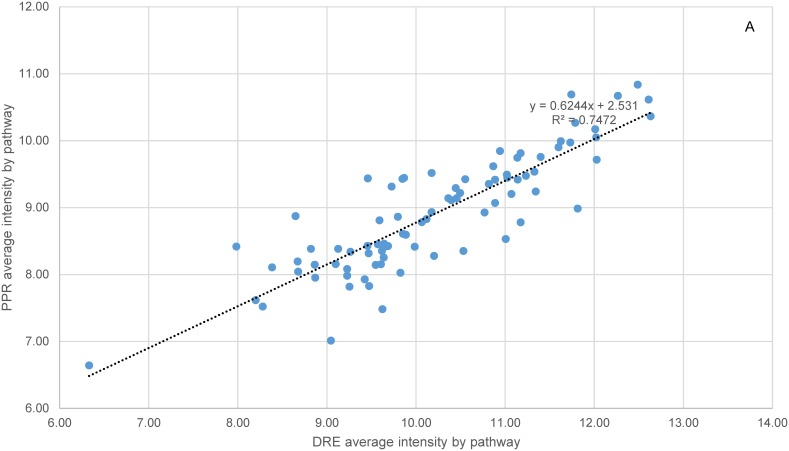
Interspecific correlation by average intensity and average coefficient of variation of individual pathways. A total of 84 (three outliers excluded) KEGG pathways were calculated for their average intensities (A), and 53 (two outliers excluded) pathways for their average CVs (B), based on a combined total of 6617 orthologous genes. To be included, each pathway must have at least five orthologs and a p-value of ≤0.1 for the correlation of the intensities or CVs of its member genes as estimated within a batch. The CCs were 0.86 and 0.80 for the average intensity and average CV by pathway respectively, with the both p-values = 0. The p-values of normality test of error distribution for linear regressions were 0.045 and 0.585 respectively.

Within-batch variation was further compared at the pathway level between the two species. There were 87 and 55 pathways significantly correlated by ortholog intensities and CVs respectively at p-value of 0.1 or less. Each of these pathways contained at least five orthologs (S4 Table). The average membership representation of these pathways (number of orthologs over total number of member genes) was 43%, over a range of 12%–75%. When the two species were compared across all the orthologs, both their gene expression intensities and the CVs were significantly correlated (Fig. 3A, 3B), with correlation coefficients (CCs) of 0.49 and 0.33, respectively, and p-values of 0. The correlation of CVs by individual pathways ranged from 0.24 (dre04010, MAPK signaling pathway; dre01100, Metabolic Pathways) to 0.85 (dre00563, Glycosylphophatidylinositol-anchor biosynthesis), and the correlation of intensities varied from 0.19 (dre03040, Spliceosome) to 0.93 (dre00790, Folate biosynthesis). Correlation and regression of average gene expression intensities of the 84 pathways (three outliers excluded) between the two species resulted in a CC of 0.86 and R^2^ of 0.75 (Fig. 4A). Similarly, a correlation and regression of the average CVs of the 53 pathways (two outliers excluded) yielded a CC of 0.80 and R^2^ of 0.64 (Fig. 4B). The p-values for both CCs were 0.

Lastly, within-batch variation was also examined at the individual gene level. In fathead minnow, 36 of the top 50 most variable genes had expression intensities below the global average of 8.5 (S5 Table). For zebrafish, the pattern was reversed, with only 18 of the top 50 having expression intensities less than the global average (S6 Table). Interestingly, many members of the vitellogenin (egg yolk precursor) gene family were found among this group of the most variable genes.

## Discussion

An ideal design to study within-batch transcriptomic variation in untreated fish samples should closely reflect the real world conditions under which these organisms are deployed for toxicity testing. These typically include random samples from a large and long term lab culture with a genetically heterogeneous background, chemical exposures and gene expression profiling conducted over an extended period of time, as well as variation in lab personnel and supplies. We leveraged data from almost 600 microarrays generated from many different studies conducted over several years to evaluate transcriptomic variation among a common batch of untreated fish samples.

Conceptually, there are at least two different approaches to assess and remove between-batch variation present among samples assembled from different studies in order to estimate transcriptomic variation among individual fish. One is to pre-process gene expression data using various algorithms so between-batch variation is removed prior to conducting any analysis of interest [9], [10]. This, in effect, creates an adjusted gene expression matrix encompassing the entire dataset. Variance, for example, could then be estimated for each probe as a measure of variation in gene expression among individual fish. Alternatively, between-batch variation could be appropriately controlled statistically in GLM and unbiased estimates could then be obtained for between- and/or within-batch variation separately. The latter approach was adopted in this study, using either the simulated reference or the designated reference method. The main advantage of the GLM method is that within- and between-batch variation is estimated simultaneously in a well-established statistical framework. Moreover, it also presents variation in the form of DEG counts, the most common measure of treatment effects utilized in transcriptomic studies.

As expected, there was significant variation between batches in both the fathead minnow and zebrafish regardless of the factors, as evidenced by both high DEG counts from modified t-tests, and graphic separation of samples by the original batches in relevant PCA plots and dendrograms. Several lines of evidence indicate that the between-batch variation has a nonsystematic impact at the transcriptome level [8]. First, regular data pre-processing including normalization designed to primarily deal with systematic biases could not remove between-batch variation. Second, the total number of genes pooled from between-batch variation under an individual factor was much greater than those from its individual pair of between-batch comparisons. In other words, each such comparison turned up a great many previously unobserved DEGs. And third, the top-ranked DEGs by p-values from between-batch variation were not enriched with any KEGG pathways, GO terms, or gene functional groups. As to the generally smaller estimate of between-batch variation observed using the simulated reference compared to the designated reference method, it is likely that the former artificially inflated the variance of each gene in the constructed reference group, thus making t-tests more conservative. Note that the five factors selected in this study to group samples are not independent, which is reflected in their many shared DEGs and the similar patterns observed in their PCA plots and clustering dendrograms. These factors may have all contributed, to a various extent, to the tendency that temporally more closely spaced batches shared a greater transcriptomic similarity to one another.

In the context of an overall low level of transcriptomic variation among fish ovary samples within the same batch, relative variability across the transcriptome, molecular pathways, and genes was still quite substantial. The estimated overall CV of 0.05 in ovary is only 10% of the previously reported fathead minnow CV across genders, tissues, and experimental conditions [21]. In both species, however, a large number of genes are at least twice as variable (CV≥0.1); and when assembled into molecular pathways, the difference in average variability and expression intensity could reach several-fold. There are at least two ways to interpret these observations. On the one hand, the generally low variability in ovary tissue should make it a suitable system to study chemicals with subtle transcriptomic effects. On the other hand, even relatively slight differences in gene expression could very well be biologically significant and consequential in some cases. Given that the transcriptomic variability in this study was assessed under the homogeneous conditions of a common tissue type from fish raised in a controlled environment to a similar reproductive maturity, at least some of these differences are probably attributable to fish ovaries at different stages of egg development.

Within-batch variation measured indirectly by DEG counts is probably both over- and under-estimated. For the factor RNA Person and Scan Date in zebrafish, the DEG counts were likely inflated because a large number of sample replicates were distributed into only two batches under each of these two factors, and between-batch variation was probably not removed completely. The other estimates were generally low suggesting less than 2% of the transcriptomes was impacted. Without knowing the exact distribution of sample variability, an even split of samples into two classes randomized over many permutations may not discover the optimum configuration where the DEG count is maximal. Exhaustively searching through all possible sample class assignments for such a configuration is computationally very resource-intensive. For example, assuming the fathead minnow has an equal batch size of 22 samples per batch (511 samples/23 batches) under the Experiment factor, dividing the samples into two classes with each containing four to 11 samples will result in 2.4×E^+6^ configurations. In this context of possible under-estimation, within-batch variation seems to be dependent on the factors upon which samples were grouped, with the factor RNA Person, which had the fewest groups, being responsible for the largest variation.

There have been a limited number of studies reporting transcriptomic variation among individuals within a population, which are similar in nature to the within-batch variation under consideration here. These studies were limited to several organisms sampled from wild or lab populations with different levels of heterozygosity. Most of them were based on utilizing technical replicates to various degrees to estimate variability. The range of variation by percentage of genes identified as DEGs is quite wide: 2 to 9% in nematode (whole organism) [31], 17 to 28% (heart) and 38 to 61% (brain) in mummichog [35], [40], 0.8% (liver), 1.9% (testis), 3.3% (kidney), and 4% (liver) in mouse [34], [41], and 11 to 83% in human (lymphoblastoid cell lines) [33], [42]. Compared to these values, the 0.2% and 1.8% of genes determined as DEGs in the present study for zebrafish and fathead minnow ovary tissue under the factor Experiment, although probably underestimated, appear to be at the lower end of the variation range. However, it is difficult to generalize here about interspecific trends in transcriptomic variation because the current fish study is limited only to ovary tissues sampled from a long term lab culture, while these previous studies vary considerably with regard to levels of genetic variation, tissue types, and degrees of technical replication.

The expression intensities of the orthologs and their variation within-batch differed considerably among the molecular pathways in the two species. For example, in both species, some of the pathways involved in basic cellular functions such as Ribosome, RNA Transport, Citrate Cycle, Cell Cycle, and RNA Polymerase tended to be less variable. Some of the metabolic pathways, on the other hand, were more variable in both species but involved in different functions. Many pathways involved in signal transduction were expressed on average at a fairly low level, while those participating in basic cellular functions like DNA replication and protein synthesis were highly expressed. Although this difference in the average variability of pathways could be explained to a considerable extent by an inverse correlation with average intensity (in other words due to technical reasons), a large portion of the variance in the average CV remains unaccounted for by the average intensity at pathway level (R^2^ = 0.46 and 0.49, Fig. 2A, 2B). Conceivably, this unexplained variance could have several contributing sources. Within an individual organism, the variances of mRNA transcripts and proteins in a population of similar cells such as those from ovary tissue were believed to be inversely proportional to their levels of expression [43], [44]. In other words, weakly expressed genes are inherently more variable. Among individuals, asynchronous egg development in fish sampled within-batch may also be linked to altered gene expression profiles [45]. Potential genetic polymorphisms among individuals could also contribute to the variability in gene expression [46], [47]. Further complicating the picture is the finding that genes with different expression variance were not randomly distributed among molecular pathways [48], [49]. Those genes with a greater network connectivity and serving more critical functions in a pathway tended to be less variable. This hypothesis is not suited for testing in the current dataset, however, because reverse engineering of gene networks requires expression data with a wide dynamic range, achieved by either experimental perturbations or taking samples with diverse phenotypes [50]. For future studies, a proper deconvolution of gene expression variability due to technical and biological reasons is essential to the interpretation of treatment-induced effects in a transcriptomics study.

The levels of within-batch variation differed among individual genes. The top 50 most variable genes, however, did not overlap between the two species, with the exception of vitellogenin. While some of these genes lack adequate annotations, asynchronous egg development in ovaries is probably a primary contributing factor to the discrepancy. This developmental heterogeneity, along with a naturally large dynamic range of expression, could also be invoked to explain in part why vitellogenin genes are among the most variable ones in fish ovary transcriptomes. The common observation of high variance linked to low expression does not fully explain vitellogenin variability in this study, at least not for zebrafish. Interestingly, in the livers of fathead minnow males, variance of vitellogenin expression increased with estrogen exposure concentration and duration [51]. If this gene is somehow intrinsically variable, it may become difficult to detect its differential expression during ovary development [52].

A comparative analysis of transcriptomic variation based on the orthologous genes between fathead minnow and zebrafish could provide significant biological insights to the integration of these two model species for toxicogenomics applications. Both are members of the family Cyprinidae, and were estimated to have shared a last common ancestor 31 million years ago [53]. In the absence of a finished fathead minnow genome and with incomplete annotations of zebrafish, a total of only 6617 orthologs between the two species were identified through indirect multi-step ID mapping. This number represents only 25% of the 26000 zebrafish protein-coding genes [54]. Given that 69% of zebrafish genes have at least one ortholog to phylogenetically distant human species, the 6617 orthologs determined here most likely represent only a subset of the two evolutionarily more closely-related fish genomes under this study. Still, the strong correlation of both the variation and expression intensity over these orthologs suggests that the overall transcriptomes of the two species are probably well conserved. Even more importantly, the strongest correlation of both variation and intensity in gene expression was observed when evaluated at the pathway level. If this high degree of conservation, reflected in both relatively static genomes and dynamic transcriptomes, and reflected in a number of currently annotated molecular pathways dedicated to critical cellular functions, extends to most of the orthologs and pathways yet to be identified and annotated, there will be scientific opportunities to integrate these two model species for a variety of toxicogenomics applications. For example, zebrafish has a very large and increasing number of gene expression profiles available to the public. The expression data from both species could be effectively combined based on their orthologs to create a reference collection of rank-ordered gene lists much greater than it is possible from the fathead minnow alone. Such a collection will enable fish connectivity mapping for studying chemical exposures and their mechanisms of action [55], [56].

In summary, achieving a better understanding of the level and extent of transcriptomic variation among untreated individuals should improve our ability to discriminate treatment effects from background “noise” and inform their biological interpretations. Significant, and most likely nonsystematic, between-batch variation found in the fathead minnow and zebrafish transcriptomes calls for its appropriate handling in their future meta-analysis. Temporally more closely spaced batches tended to share a greater transcriptomic similarity among one another. The overall low level of within-batch transcriptomic variation in fish ovary tissue, on the other hand, makes it a suitable system for studying chemical stressors with subtle biological effects. The observed differences in the within-batch variability of gene expression, at the levels of both individual genes and pathways, were probably both technical and biological. This suggests that biological interpretation and prioritization of genes and pathways targeted by experimental conditions should take into account both their intrinsic variability and the size of induced transcriptional changes. An intrinsically less variable gene or pathway with a slight change in expression might be just as informative for evaluating treatment effects as the highly expressed but more variable one. The significant conservation of both the genomes and transcriptomes between the fathead minnow and zebrafish over currently identified orthologs suggests promising opportunities in not only studying fish molecular responses to environmental stressors by a comparative biology approach, but also effective sharing of a large amount of existing public transcriptomics data for developing toxicogenomics applications.

## Supporting Information

S1 Table **Cross-mapped probes from Agilent design 015064 (zebrafish) and 019597 (fathead minnow) via their orthologs.** A total of 9311 probes from Agilent 015064 were linked to 6950 probes from Agilent 019597 through 6617 common Entrez GeneIDs in NCBI.
(XLSX)

S2 Table **The DAVID analysis of fathead minnow DEGs.** The top 100 DEGs were combined from the between-batch variation under each factor for possible enrichment in biological pathways.
(PDF)

S3 Table **The DAVID analysis of zebrafish DEGs.** The top 100 DEGs were combined from the between-batch variation under each factor for possible enrichment in biological pathways.
(PDF)

S4 Table **Interspecific comparison of within-batch variation at the molecular pathway level.** A total of 6617 orthologous genes were grouped into 144 out of the 162 KEGG pathways as of April, 2014. The intensity and CV were calculated by individual batches each containing multiple biological replicates under the Experiment factor, and then averaged over all batches. Only pathways with at least five orthologs were included. KEGG, Kyoto Encyclopedia of Genes and Genomes; DRE, zebrafish; PPR, fathead minnow.
(XLSX)

S5 Table **The top 50 most variable fathead minnow (PPR) genes based on average within-batch coefficient of variation (CV) under the Experiment factor.**
(DOCX)

S6 Table **The top 50 most variable zebrafish (DRE) genes based on average within-batch coefficient of variation (CV) under the Experiment factor.**
(DOCX)

S1 File **Average number of DEGs per pair of between-batch comparison in 1000 permutations.** Fathead minnow (Figure S1A) critical cutoffs were: 5%, 15; 1%, 27; 0.1%, 104. Zebrafish (Figure S1B) critical cutoffs were: 5%, 418; 1%, 479; 0.1%, 594. Samples were grouped by the factor Experiment.
(PDF)

S2 File **The PCA plots of fathead minnow samples based on all the DEGs identified as between-batch variation.** Samples were grouped by Experiment (Figure S2A, S2B), RNA Date (Figure S3A, S3B), RNA Person (Figure S4A, S4B), Sampling Date (Figure S5A, S5B), and Scan Date (Figure S6A, S6B). Each figure was based on either the average gene intensity by individual batches (A) or the gene intensity of individual samples (B). DEGs were based on the simulated reference method.
(PDF)

S3 File **The PCA plots of zebrafish samples based on all the DEGs identified as between-batch variation.** Samples were grouped by Experiment (Figure S7A, S7B), RNA Date (Figure S8A, S8B), RNA Person (Figure S9A, S9B), Sampling Date (Figure S10A, S10B), and Scan Date (Figure S11A, S11B). Each figure was based on either the average gene intensity by individual batches (A) or the gene intensity of individual samples (B). DEGs were based on the simulated reference method.
(PDF)

S4 File **The dendrograms from resampling clustering of fathead minnow samples based on all the DEGs identified as between-batch variation.** Samples were grouped by Experiment (Figure S12A, S12B), RNA Date (Figure S13A, S13B), RNA Person (Figure S14A, S14B), Sampling Date (Figure S15A, S15B), and Scan Date (Figure S16A, S16B). Each figure was based on either the average gene intensity by individual batches (A) or the gene intensity of individual samples (B). DEGs were based on the simulated reference method.
(PDF)

S5 File **The dendrogram from resampling clustering of zebrafish samples based on all the DEGs identified as between-batch variation.** Samples were grouped by Experiment (Figure S17A, S17B), RNA Date (Figure S18A, S18B), RNA Person (Figure S19A, S19B), Sampling Date (Figure S20A, S20B), and Scan Date (Figure S21A, S21B). Each figure was based on either the average gene intensity by individual batches (A) or the gene intensity of individual samples (B). DEGs were based on the simulated reference method.
(PDF)

S6 File **The distribution of within-batch coefficients of variation (CV) of probe intensities.** Fathead minnow (Figure S22A) CVs were based on 15208 probes, and zebrafish (Figure S22B) CVs were based on 21495 probes.
(PDF)
