# Correction: Natural Variation in Fish Transcriptomes: Comparative Analysis of the Fathead Minnow (*Pimephales promelas*) and Zebrafish (*Danio rerio*)

**DOI:** 10.1371/journal.pone.0118398

**Published:** 2015-03-18

**Authors:** The PLOS ONE Staff

Panel B of each figure is missing. Please see the complete Fig. 1, Fig. 2, Fig. 3, and Fig. 4 here:

**Fig 1:**
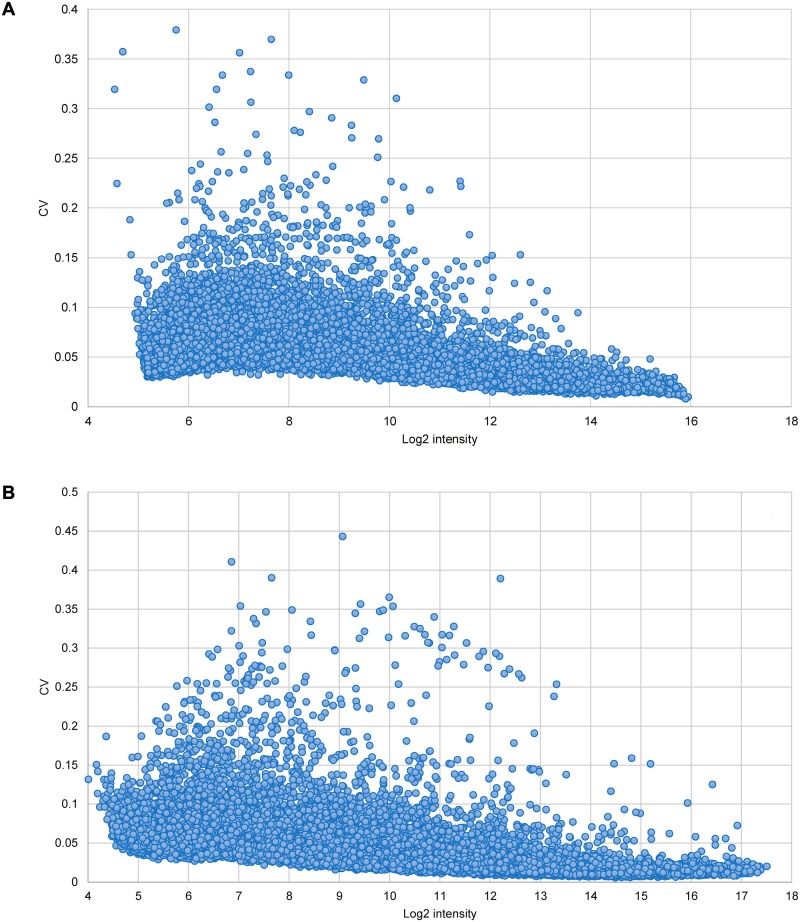
Estimation of within-batch variation. Coefficients of variation (CV) were computed at various intensities of 15208 fathead minnow probes (A) and 21495 zebrafish probes (B).

**Fig 2:**
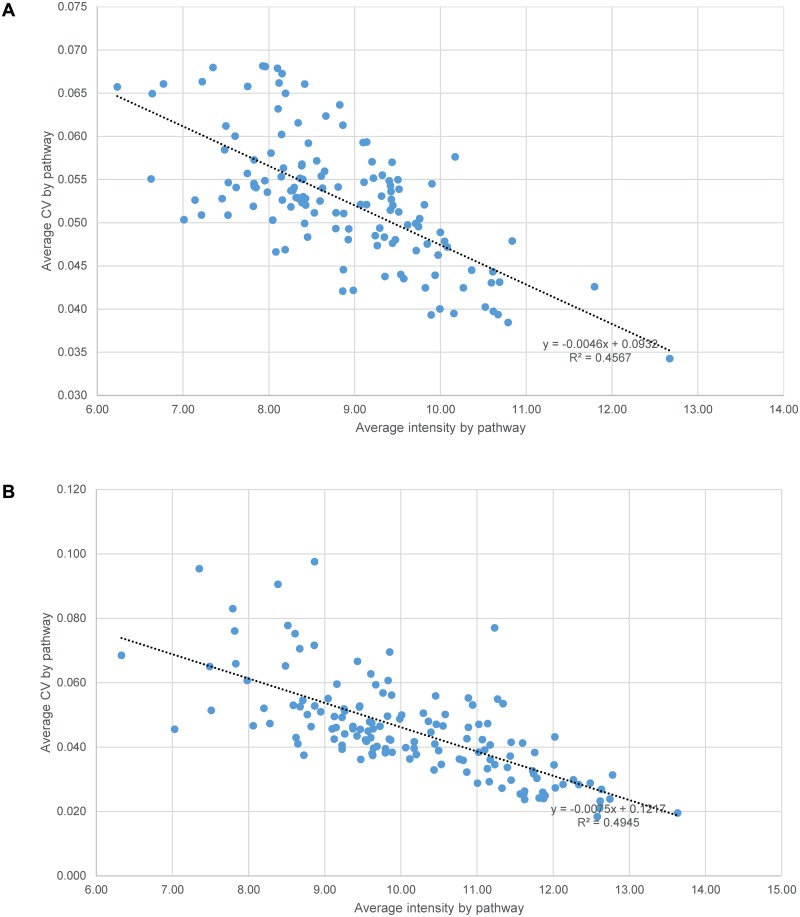
Intraspecific correlation between the average CV and average intensity by KEGG pathways. A total of 136 pathways (eight outliers excluded) were included for fathead minnow (A) and 144 pathways for zebrafish (B). The CCs were -0.68 and -0.70 respectively, with both p-values = 0. The p-values of normality test of error distribution for linear regressions were 0.094 (no significant departure from normality) and 0 (significant non-normality) respectively.

**Fig 3:**
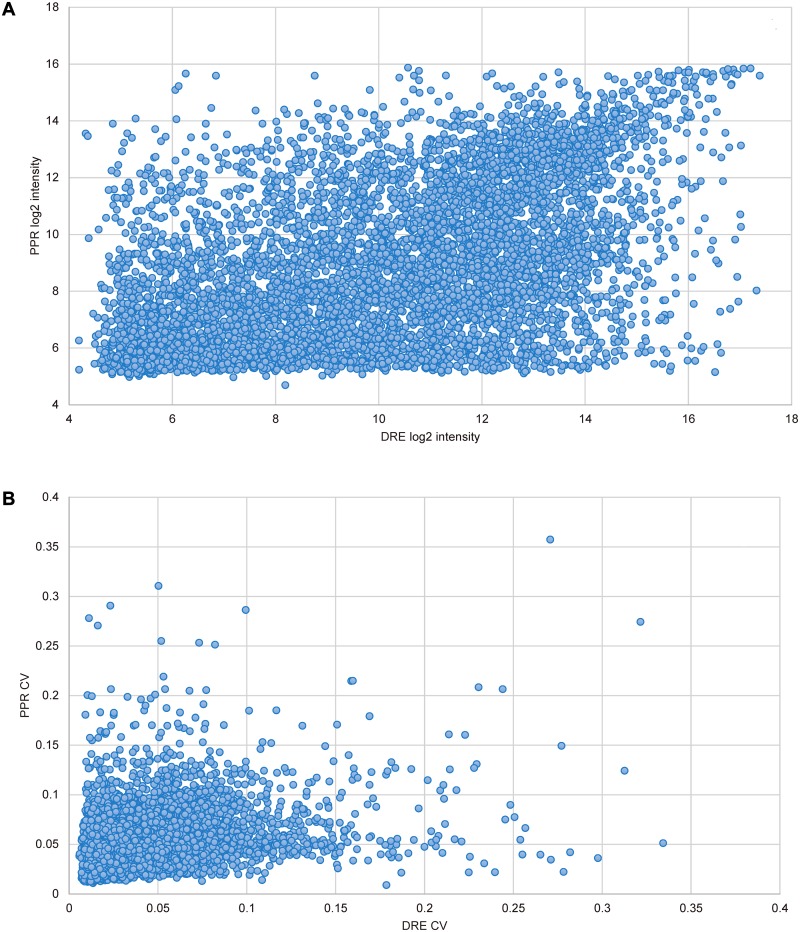
Interspecific correlation by within-batch intensity and coefficient of variation of orthologs. The within-batch intensities (A) and coefficients of variation (CV; B) were based on 6617 orthologous genes. The orthologs were represented by 9311 zebrafish (DRE) and 6950 fathead minnow (PPR) probes. The intensity and CV of an ortholog with duplicated probes were probe means. The correlation coefficients over the orthologs for the two metrics were 0.49 and 0.33 respectively, with the both p-values = 0.

**Fig 4:**
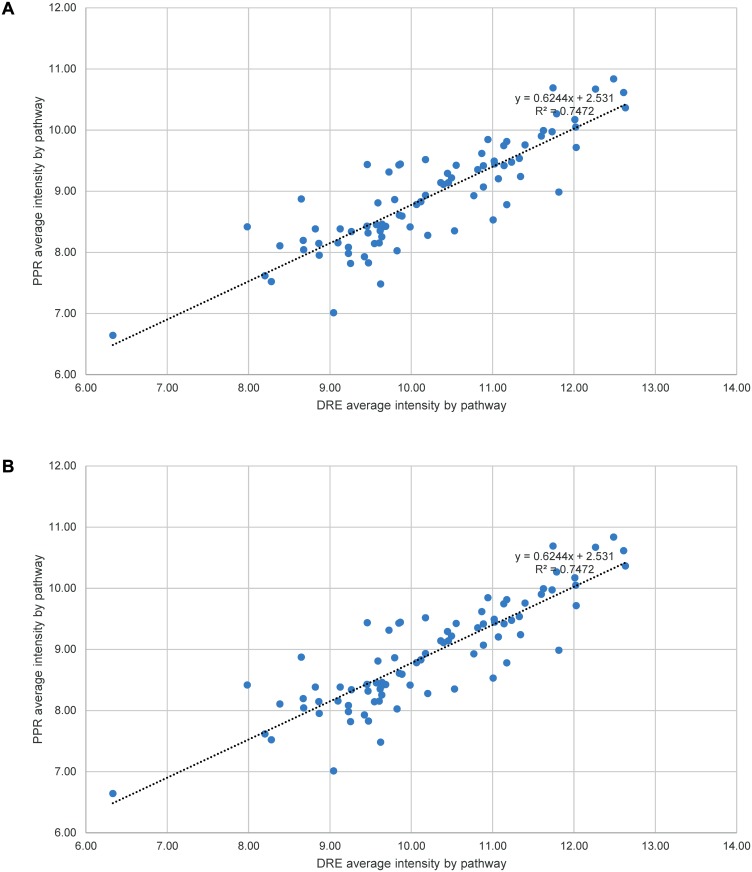
Interspecific correlation by average intensity and average coefficient of variation of individual pathways. A total of 84 (three outliers excluded) KEGG pathways were calculated for their average intensities (A), and 53 (two outliers excluded) pathways for their average CVs (B), based on a combined total of 6617 orthologous genes. To be included, each pathway must have at least five orthologs and a p-value of ≤0.1 for the correlation of the intensities or CVs of its member genes as estimated within a batch. The CCs were 0.86 and 0.80 for the average intensity and average CV by pathway respectively, with the both p-values = 0. The p-values of normality test of error distribution for linear regressions were 0.045 and 0.585 respectively.

## References

[1] Wang R-L, Bencic DC, Garcia-Reyero N, Perkins EJ, Villeneuve DL, Ankley GT, et al (2014) Natural Variation in Fish Transcriptomes: Comparative Analysis of the Fathead Minnow (*Pimephales promelas)* and Zebrafish (*Danio rerio*). PLoS ONE 9(12): e114178 doi: 10.1371/journal.pone.0114178 25493933 10.1371/journal.pone.0114178 PMC4262388

