# Supplementary material for: Natural Variation in Fish Transcriptomes: Comparative Analysis of the Fathead Minnow (Pimephales promelas) and Zebrafish (Danio rerio)
Source: PLoS One. 2014 Dec 10;9(12):e114178. doi: 10.1371/journal.pone.0114178 (PMC4262388; doi:10.1371/journal.pone.0114178)
Supplement: S2 Table — The DAVID analysis of fathead minnow DEGs. The top 100 DEGs were combined from the between-batch variation under each factor for possible enrichment in biological pathways. (PDF) [file pone.0114178.s002.pdf]

DAVID Bioinformatics Resources 6.7
National Institute of Allergy and Infectious Diseases (NIAID), NIH

Functional Annotation Clustering

Help and Manual

Current Gene List: BetweenBatch_FHM_InVivoTop100byFpvalue.combined
Current Background: Danio rerio
258 DAVID IDs

Options **Classification Stringency** Medium

Rerun using options Create Sublist

28 Cluster(s)

Download File

				Count	P_Value	Benjamini
Annotation Cluster 1		Enrichment Score: 1.99	G	Count	P_Value	Benjamini
KEGG_PATHWAY	Spliceosome	RT		7	3.8E-3	1.7E-1
KEGG_PATHWAY	RNA degradation	RT		5	6.2E-3	1.4E-1
INTERPRO	Like-Sm ribonucleoprotein, eukaryotic and archaea-type, core	RT		3	1.5E-2	9.5E-1
INTERPRO	Like-Sm ribonucleoprotein, core	RT		3	1.7E-2	8.9E-1
SMART	Sm	RT		3	1.9E-2	5.9E-1
Annotation Cluster 2		Enrichment Score: 1.51	G	Count	P_Value	Benjamini
INTERPRO	Zinc finger, PHD-finger	RT		5	1.2E-2	9.9E-1
INTERPRO	Zinc finger, PHD-type	RT		5	1.2E-2	9.9E-1
SMART	PHD	RT		5	1.8E-2	8.1E-1
INTERPRO	Zinc finger, PHD-type, conserved site	RT		4	3.9E-2	9.5E-1
SP_PIR_KEYWORDS	zinc	RT		13	3.0E-1	9.4E-1
Annotation Cluster 3		Enrichment Score: 1.12	G	Count	P_Value	Benjamini
GOTERM_MF_FAT	translation factor activity, nucleic acid binding	RT		5	2.4E-2	8.4E-1
SP_PIR_KEYWORDS	Initiation factor	RT		3	1.1E-1	9.1E-1
GOTERM_MF_FAT	translation initiation factor activity	RT		3	1.6E-1	9.9E-1
Annotation Cluster 4		Enrichment Score: 1.1	G	Count	P_Value	Benjamini
INTERPRO	DNA/RNA helicase, C-terminal	RT		4	6.1E-2	9.8E-1
INTERPRO	DEAD-like helicase, N-terminal	RT		4	6.3E-2	9.7E-1
INTERPRO	Helicase, superfamily 1 and 2, ATP-binding	RT		4	6.6E-2	9.6E-1
SMART	HELICc	RT		4	8.1E-2	9.3E-1
SMART	DEXDc	RT		4	8.3E-2	8.7E-1
GOTERM_MF_FAT	helicase activity	RT		4	1.4E-1	1.0E0
Annotation Cluster 5		Enrichment Score: 1.02	G	Count	P_Value	Benjamini
GOTERM_MF_FAT	translation factor activity, nucleic acid binding	RT		5	2.4E-2	8.4E-1
SP_PIR_KEYWORDS	protein biosynthesis	RT		3	1.4E-1	8.9E-1
GOTERM_BP_FAT	translation	RT		6	2.7E-1	1.0E0
Annotation Cluster 6		Enrichment Score: 1	G	Count	P_Value	Benjamini
UP_SEQ_FEATURE	DNA-binding region:Homeobox	RT		6	1.7E-2	8.5E-1
GOTERM_BP_FAT	regulation of transcription	RT		23	3.0E-2	1.0E0
GOTERM_BP_FAT	transcription	RT		13	4.1E-2	1.0E0
SP_PIR_KEYWORDS	Transcription	RT		12	4.5E-2	9.9E-1
SP_PIR_KEYWORDS	transcription regulation	RT		12	4.8E-2	9.2E-1
INTERPRO	Homeobox, conserved site	RT		7	7.0E-2	9.5E-1
SP_PIR_KEYWORDS	developmental protein	RT		9	7.9E-2	9.4E-1
GOTERM_MF_FAT	DNA binding	RT		21	1.1E-1	1.0E0
SP_PIR_KEYWORDS	Homeobox	RT		7	1.1E-1	8.7E-1
INTERPRO	Homeobox	RT		7	1.2E-1	9.8E-1
GOTERM_BP_FAT	regulation of transcription, DNA-[illegible]	RT		16	1.3E-1	1.0E0

	dependent				
INTERPRO	Homeodomain-related	RT	7	1.3E-1	9.8E-1
GOTERM_BP_FAT	regulation of RNA metabolic process	RT	16	1.4E-1	1.0E0
SP_PIR_KEYWORDS	dna-binding	RT	13	1.4E-1	8.7E-1
GOTERM_MF_FAT	transcription factor activity	RT	13	1.5E-1	9.9E-1
SMART	HOX	RT	7	1.8E-1	9.5E-1
GOTERM_MF_FAT	transcription regulator activity	RT	15	2.5E-1	9.9E-1
SP_PIR_KEYWORDS	nucleus	RT	18	3.2E-1	9.4E-1
GOTERM_MF_FAT	sequence-specific DNA binding	RT	9	3.7E-1	9.8E-1
Annotation Cluster 7	Enrichment Score: 0.96	G	Count	P_Value	Benjamini
GOTERM_BP_FAT	cell part morphogenesis	RT	5	3.9E-2	1.0E0
GOTERM_BP_FAT	cellular component morphogenesis	RT	6	5.3E-2	9.9E-1
GOTERM_BP_FAT	cell morphogenesis	RT	5	8.7E-2	1.0E0
GOTERM_BP_FAT	cell projection morphogenesis	RT	4	1.3E-1	1.0E0
GOTERM_BP_FAT	cell projection organization	RT	4	2.0E-1	1.0E0
GOTERM_BP_FAT	cell motion	RT	4	3.9E-1	1.0E0
Annotation Cluster 8	Enrichment Score: 0.93	G	Count	P_Value	Benjamini
GOTERM_BP_FAT	determination of bilateral symmetry	RT	4	9.3E-2	1.0E0
GOTERM_BP_FAT	determination of symmetry	RT	4	9.3E-2	1.0E0
GOTERM_BP_FAT	pattern specification process	RT	7	1.1E-1	1.0E0
GOTERM_BP_FAT	determination of left/right symmetry	RT	3	2.1E-1	1.0E0
Annotation Cluster 9	Enrichment Score: 0.64	G	Count	P_Value	Benjamini
GOTERM_CC_FAT	integral to organelle membrane	RT	3	1.1E-1	1.0E0
GOTERM_CC_FAT	intrinsic to organelle membrane	RT	3	1.5E-1	1.0E0
GOTERM_CC_FAT	endomembrane system	RT	4	3.0E-1	1.0E0
GOTERM_CC_FAT	organelle membrane	RT	4	5.8E-1	1.0E0
Annotation Cluster 10	Enrichment Score: 0.6	G	Count	P_Value	Benjamini
INTERPRO	Src homology-3 domain	RT	5	1.6E-1	9.9E-1
SMART	SH3	RT	5	2.2E-1	9.6E-1
SP_PIR_KEYWORDS	sh3 domain	RT	3	4.4E-1	9.7E-1
Annotation Cluster 11	Enrichment Score: 0.58	G	Count	P_Value	Benjamini
GOTERM_BP_FAT	pattern specification process	RT	7	1.1E-1	1.0E0
GOTERM_BP_FAT	somitogenesis	RT	3	2.4E-1	1.0E0
GOTERM_BP_FAT	embryonic development ending in birth or egg hatching	RT	4	2.6E-1	1.0E0
GOTERM_BP_FAT	chordate embryonic development	RT	4	2.6E-1	1.0E0
GOTERM_BP_FAT	segmentation	RT	3	2.7E-1	1.0E0
GOTERM_BP_FAT	regionalization	RT	4	4.4E-1	1.0E0
GOTERM_BP_FAT	anterior/posterior pattern formation	RT	3	4.6E-1	1.0E0
Annotation Cluster 12	Enrichment Score: 0.52	G	Count	P_Value	Benjamini
SP_PIR_KEYWORDS	transit peptide	RT	3	2.0E-1	9.2E-1
GOTERM_BP_FAT	translation	RT	6	2.7E-1	1.0E0
UP_SEQ_FEATURE	transit peptide:Mitochondrion	RT	3	2.8E-1	1.0E0
SP_PIR_KEYWORDS	mitochondrion	RT	3	5.6E-1	9.8E-1
Annotation Cluster 13	Enrichment Score: 0.49	G	Count	P_Value	Benjamini
INTERPRO	Small GTP-binding protein	RT	5	2.1E-1	1.0E0
GOTERM_MF_FAT	GTPase activity	RT	3	2.2E-1	1.0E0
INTERPRO	Ras	RT	4	2.6E-1	1.0E0
SP_PIR_KEYWORDS	gtp-binding	RT	6	2.7E-1	9.5E-1
INTERPRO	Ras GTPase	RT	4	3.1E-1	1.0E0
GOTERM_MF_FAT	GTP binding	RT	7	3.2E-1	9.9E-1
GOTERM_MF_FAT	guanyl ribonucleotide binding	RT	7	3.3E-1	9.9E-1
GOTERM_MF_FAT	guanyl nucleotide binding	RT	7	3.3E-1	9.9E-1
SP_PIR_KEYWORDS	nucleotide-binding	RT	14	4.6E-1	9.6E-1

GOTERM_BP_FAT	intracellular signaling cascade	RT	7	4.7E-1	1.0E0
GOTERM_BP_FAT	small GTPase mediated signal transduction	RT	4	5.1E-1	1.0E0
Annotation Cluster 14	Enrichment Score: 0.42	G	Count	P_Value	Benjamini
GOTERM_BP_FAT	embryonic organ morphogenesis	RT	3	3.0E-1	1.0E0
GOTERM_BP_FAT	embryonic organ development	RT	4	3.1E-1	1.0E0
GOTERM_BP_FAT	embryonic morphogenesis	RT	4	5.8E-1	1.0E0
Annotation Cluster 15	Enrichment Score: 0.39	G	Count	P_Value	Benjamini
SP_PIR_KEYWORDS	zinc	RT	13	3.0E-1	9.4E-1
SP_PIR_KEYWORDS	zinc-finger	RT	7	3.7E-1	9.6E-1
INTERPRO	Zinc finger, RING-type	RT	5	4.2E-1	1.0E0
INTERPRO	Zinc finger, C3HC4 RING-type	RT	4	4.7E-1	1.0E0
SMART	RING	RT	5	5.1E-1	1.0E0
Annotation Cluster 16	Enrichment Score: 0.39	G	Count	P_Value	Benjamini
GOTERM_CC_FAT	nucleoplasm part	RT	3	3.5E-1	1.0E0
GOTERM_CC_FAT	intracellular organelle lumen	RT	4	3.8E-1	1.0E0
GOTERM_CC_FAT	organelle lumen	RT	4	3.8E-1	1.0E0
GOTERM_CC_FAT	nucleoplasm	RT	3	4.0E-1	9.9E-1
GOTERM_CC_FAT	membrane-enclosed lumen	RT	4	4.1E-1	9.9E-1
GOTERM_CC_FAT	nuclear lumen	RT	3	5.7E-1	1.0E0
Annotation Cluster 17	Enrichment Score: 0.34	G	Count	P_Value	Benjamini
GOTERM_BP_FAT	translation	RT	6	2.7E-1	1.0E0
GOTERM_CC_FAT	ribonucleoprotein complex	RT	5	2.8E-1	1.0E0
SP_PIR_KEYWORDS	ribosomal protein	RT	3	4.8E-1	9.6E-1
GOTERM_CC_FAT	ribosome	RT	3	5.2E-1	1.0E0
GOTERM_MF_FAT	structural constituent of ribosome	RT	3	5.5E-1	9.9E-1
GOTERM_MF_FAT	structural molecule activity	RT	3	9.2E-1	1.0E0
Annotation Cluster 18	Enrichment Score: 0.32	G	Count	P_Value	Benjamini
INTERPRO	Zinc finger, C2H2-type	RT	8	3.6E-1	1.0E0
INTERPRO	Zinc finger, C2H2-like	RT	7	4.9E-1	1.0E0
SMART	ZnF_C2H2	RT	7	6.1E-1	1.0E0
Annotation Cluster 19	Enrichment Score: 0.3	G	Count	P_Value	Benjamini
INTERPRO	Serine/threonine protein kinase	RT	5	2.6E-1	1.0E0
GOTERM_MF_FAT	purine ribonucleotide binding	RT	21	3.0E-1	9.9E-1
GOTERM_MF_FAT	ribonucleotide binding	RT	21	3.0E-1	9.9E-1
GOTERM_MF_FAT	nucleotide binding	RT	25	3.3E-1	9.8E-1
SMART	S_TKc	RT	5	3.3E-1	9.9E-1
GOTERM_MF_FAT	purine nucleotide binding	RT	21	3.7E-1	9.9E-1
INTERPRO	Serine/threonine protein kinase-related	RT	6	4.3E-1	1.0E0
INTERPRO	Serine/threonine protein kinase, active site	RT	5	4.5E-1	1.0E0
SP_PIR_KEYWORDS	nucleotide-binding	RT	14	4.6E-1	9.6E-1
GOTERM_MF_FAT	ATP binding	RT	15	4.7E-1	9.9E-1
GOTERM_MF_FAT	adenyl ribonucleotide binding	RT	15	4.7E-1	9.9E-1
GOTERM_MF_FAT	adenyl nucleotide binding	RT	15	5.4E-1	9.9E-1
GOTERM_MF_FAT	purine nucleoside binding	RT	15	5.5E-1	9.9E-1
GOTERM_MF_FAT	protein kinase activity	RT	7	5.5E-1	9.9E-1
GOTERM_MF_FAT	nucleoside binding	RT	15	5.6E-1	9.9E-1
GOTERM_BP_FAT	phosphorylation	RT	8	5.7E-1	1.0E0
GOTERM_BP_FAT	protein amino acid phosphorylation	RT	7	5.8E-1	1.0E0
GOTERM_MF_FAT	protein serine/threonine kinase activity	RT	5	5.8E-1	9.9E-1
INTERPRO	Protein kinase, core	RT	6	6.2E-1	1.0E0
GOTERM_BP_FAT	phosphorus metabolic process	RT	9	6.5E-1	1.0E0
GOTERM_BP_FAT	phosphate metabolic process	RT	9	6.5E-1	1.0E0
SP_PIR_KEYWORDS	atp-binding	RT	[illegible]	[illegible]	[illegible]

[illegible]	atp-binding	RT	8	8.0E-1	1.0E0
SP_PIR_KEYWORDS	serine/threonine-protein kinase	RT	3	8.2E-1	1.0E0
INTERPRO	Protein kinase, ATP binding site	RT	3	9.4E-1	1.0E0
Annotation Cluster 20	Enrichment Score: 0.29	G	Count	P_Value	Benjamini
GOTERM_BP_FAT	ubiquitin-dependent protein catabolic process	RT	3	1.9E-1	1.0E0
GOTERM_BP_FAT	cellular macromolecule catabolic process	RT	5	2.9E-1	1.0E0
GOTERM_BP_FAT	macromolecule catabolic process	RT	5	3.9E-1	1.0E0
GOTERM_BP_FAT	modification-dependent protein catabolic process	RT	3	6.6E-1	1.0E0
GOTERM_BP_FAT	modification-dependent macromolecule catabolic process	RT	3	6.6E-1	1.0E0
GOTERM_BP_FAT	proteolysis involved in cellular protein catabolic process	RT	3	7.1E-1	1.0E0
GOTERM_BP_FAT	cellular protein catabolic process	RT	3	7.1E-1	1.0E0
GOTERM_BP_FAT	proteolysis	RT	7	7.5E-1	1.0E0
GOTERM_BP_FAT	protein catabolic process	RT	3	7.5E-1	1.0E0
Annotation Cluster 21	Enrichment Score: 0.28	G	Count	P_Value	Benjamini
SP_PIR_KEYWORDS	egf-like domain	RT	3	4.4E-1	9.7E-1
INTERPRO	EGF-like region, conserved site	RT	4	4.5E-1	1.0E0
INTERPRO	EGF-like, type 3	RT	3	4.8E-1	1.0E0
INTERPRO	EGF-like	RT	3	5.2E-1	1.0E0
SMART	EGF	RT	3	5.9E-1	1.0E0
GOTERM_CC_FAT	extracellular region	RT	5	6.9E-1	1.0E0
Annotation Cluster 22	Enrichment Score: 0.26	G	Count	P_Value	Benjamini
INTERPRO	EF-HAND 2	RT	4	4.4E-1	1.0E0
INTERPRO	EF-Hand type	RT	4	4.7E-1	1.0E0
INTERPRO	Calcium-binding EF-hand	RT	3	5.4E-1	1.0E0
SMART	EFh	RT	3	6.0E-1	1.0E0
GOTERM_MF_FAT	calcium ion binding	RT	6	7.2E-1	1.0E0
Annotation Cluster 23	Enrichment Score: 0.26	G	Count	P_Value	Benjamini
SP_PIR_KEYWORDS	zinc	RT	13	3.0E-1	9.4E-1
GOTERM_MF_FAT	zinc ion binding	RT	21	4.4E-1	9.9E-1
SP_PIR_KEYWORDS	metal-binding	RT	15	4.6E-1	9.6E-1
GOTERM_MF_FAT	transition metal ion binding	RT	23	6.4E-1	1.0E0
GOTERM_MF_FAT	metal ion binding	RT	30	7.3E-1	1.0E0
GOTERM_MF_FAT	cation binding	RT	30	7.6E-1	1.0E0
GOTERM_MF_FAT	ion binding	RT	30	7.6E-1	1.0E0
Annotation Cluster 24	Enrichment Score: 0.25	G	Count	P_Value	Benjamini
GOTERM_BP_FAT	sensory organ development	RT	4	5.3E-1	1.0E0
GOTERM_BP_FAT	eye development	RT	3	5.8E-1	1.0E0
GOTERM_BP_FAT	embryonic morphogenesis	RT	4	5.8E-1	1.0E0
Annotation Cluster 25	Enrichment Score: 0.22	G	Count	P_Value	Benjamini
INTERPRO	WD40 repeat, conserved site	RT	4	2.9E-1	1.0E0
SP_PIR_KEYWORDS	wd repeat	RT	3	6.5E-1	9.9E-1
INTERPRO	WD40 repeat	RT	3	7.1E-1	1.0E0
INTERPRO	WD40/YVTN repeat-like	RT	3	7.7E-1	1.0E0
SMART	WD40	RT	3	7.8E-1	1.0E0
Annotation Cluster 26	Enrichment Score: 0.21	G	Count	P_Value	Benjamini
GOTERM_BP_FAT	protein localization	RT	6	4.1E-1	1.0E0
GOTERM_BP_FAT	establishment of protein localization	RT	4	7.6E-1	1.0E0
GOTERM_BP_FAT	protein transport	RT	4	7.6E-1	1.0E0
Annotation Cluster 27	Enrichment Score: 0.11	G	Count	P_Value	Benjamini
GOTERM_MF_FAT	endopeptidase activity	RT	4	7.4E-1	1.0E0
GOTERM_BP_FAT	proteolysis	RT	7	7.5E-1	1.0E0
GOTERM_MF_FAT	peptidase activity, acting on L-amino acid peptides	RT	5	8.0E-1	1.0E0
GOTERM_MF_FAT	[illegible]				

GOTERM_MF_FAT	peptidase activity	RT		5	8.3E-1	1.0E0
Annotation Cluster 28	Enrichment Score: 0.05	G		Count	P_Value	Benjamini
SP_PIR_KEYWORDS	glycoprotein	RT		5	5.8E-1	9.8E-1
UP_SEQ_FEATURE	glycosylation site:N-linked (GlcNAc...)	RT		5	7.6E-1	1.0E0
SP_PIR_KEYWORDS	membrane	RT		15	9.3E-1	1.0E0
UP_SEQ_FEATURE	topological domain:Cytoplasmic	RT		3	9.4E-1	1.0E0
UP_SEQ_FEATURE	transmembrane region	RT		6	9.7E-1	1.0E0
GOTERM_CC_FAT	intrinsic to membrane	RT		16	9.9E-1	1.0E0
GOTERM_CC_FAT	integral to membrane	RT		15	1.0E0	1.0E0
SP_PIR_KEYWORDS	transmembrane	RT		10	1.0E0	1.0E0

59 terms were not clustered.

Please cite *Nature Protocols 2009; 4(1):44 & Genome Biology 2003; 4(5):P3* within any publication that makes use of any methods inspired by **DAVID**.

Term of Service | Contact Us | Site Map
