# Supplementary material for: Natural Variation in Fish Transcriptomes: Comparative Analysis of the Fathead Minnow (Pimephales promelas) and Zebrafish (Danio rerio)
Source: PLoS One. 2014 Dec 10;9(12):e114178. doi: 10.1371/journal.pone.0114178 (PMC4262388; doi:10.1371/journal.pone.0114178)
Supplement: S3 Table — The DAVID analysis of zebrafish DEGs. The top 100 DEGs were combined from the between-batch variation under each factor for possible enrichment in biological pathways. (PDF) [file pone.0114178.s003.pdf]

DAVID Bioinformatics Resources 6.7

National Institute of Allergy and Infectious Diseases (NIAID), NIH

Functional Annotation Clustering

Help and Manual

Current Gene List: BetweenBatch_DREtop100byFpvalueCombined.uniq_015064_D_AA_20131128.NCBIids

Current Background: Danio rerio

102 DAVID IDs

Options **Classification Stringency** Medium

Rerun using options | Create Sublist

7 Cluster(s)

Download File

	Category	Term		Count	P_Value	Benjamini
	Annotation Cluster 1	Enrichment Score: 2.52	G	Count	P_Value	Benjamini
☐	INTERPRO	Nucleotide-binding, alpha-beta plait	RT	7	7.6E-4	1.5E-1
☐	SMART	RRM	RT	6	2.1E-3	8.4E-2
☐	INTERPRO	RNA recognition motif, RNP-1	RT	6	4.9E-3	4.0E-1
☐	KEGG_PATHWAY	Spliceosome	RT	5	1.1E-2	3.0E-1
	Annotation Cluster 2	Enrichment Score: 1.25	G	Count	P_Value	Benjamini
☐	SP_PIR_KEYWORDS	ribonucleoprotein	RT	6	1.2E-3	8.6E-2
☐	GOTERM_CC_FAT	ribonucleoprotein complex	RT	7	1.4E-2	7.1E-1
☐	SP_PIR_KEYWORDS	viral nucleoprotein	RT	3	2.2E-2	4.2E-1
☐	GOTERM_BP_FAT	translation	RT	5	4.5E-2	9.9E-1
☐	SP_PIR_KEYWORDS	ribosomal protein	RT	4	5.6E-2	6.6E-1
☐	GOTERM_MF_FAT	structural molecule activity	RT	6	7.2E-2	9.9E-1
☐	GOTERM_MF_FAT	structural constituent of ribosome	RT	4	7.7E-2	9.6E-1
☐	GOTERM_CC_FAT	non-membrane-bounded organelle	RT	9	1.3E-1	1.0E0
☐	GOTERM_CC_FAT	intracellular non-membrane-bounded organelle	RT	9	1.3E-1	1.0E0
☐	KEGG_PATHWAY	Ribosome	RT	3	1.3E-1	7.8E-1
☐	GOTERM_CC_FAT	ribosome	RT	4	1.4E-1	9.9E-1

	GOTERM_MF_FAT	RNA binding	RT		3	5.6E-1	1.0E0
	Annotation Cluster 3	Enrichment Score: 0.45	G		Count	P_Value	Benjamini
	GOTERM_MF_FAT	nucleotide binding	RT		19	3.6E-2	9.9E-1
	SP_PIR_KEYWORDS	nucleotide-binding	RT		9	3.4E-1	1.0E0
	SP_PIR_KEYWORDS	atp-binding	RT		7	3.5E-1	9.9E-1
	GOTERM_MF_FAT	ATP binding	RT		9	4.3E-1	1.0E0
	GOTERM_MF_FAT	adenyl ribonucleotide binding	RT		9	4.4E-1	1.0E0
	GOTERM_MF_FAT	purine ribonucleotide binding	RT		11	4.9E-1	1.0E0
	GOTERM_MF_FAT	ribonucleotide binding	RT		11	4.9E-1	1.0E0
	GOTERM_MF_FAT	adenyl nucleotide binding	RT		9	4.9E-1	1.0E0
	GOTERM_MF_FAT	purine nucleoside binding	RT		9	5.0E-1	1.0E0
	GOTERM_MF_FAT	nucleoside binding	RT		9	5.0E-1	1.0E0
	GOTERM_MF_FAT	purine nucleotide binding	RT		11	5.4E-1	1.0E0
	Annotation Cluster 4	Enrichment Score: 0.18	G		Count	P_Value	Benjamini
	SMART	RING	RT		3	3.6E-1	1.0E0
	INTERPRO	Zinc finger, RING-type	RT		3	4.4E-1	1.0E0
	SP_PIR_KEYWORDS	zinc	RT		7	4.6E-1	9.9E-1
	SP_PIR_KEYWORDS	metal-binding	RT		7	7.4E-1	1.0E0
	SP_PIR_KEYWORDS	zinc-finger	RT		3	7.5E-1	1.0E0
	GOTERM_MF_FAT	cation binding	RT		16	7.8E-1	1.0E0
	GOTERM_MF_FAT	ion binding	RT		16	7.8E-1	1.0E0
	GOTERM_MF_FAT	transition metal ion binding	RT		11	8.3E-1	1.0E0
	GOTERM_MF_FAT	zinc ion binding	RT		9	8.3E-1	1.0E0
	GOTERM_MF_FAT	metal ion binding	RT		15	8.4E-1	1.0E0
	Annotation Cluster 5	Enrichment Score: 0.17	G		Count	P_Value	Benjamini
	SP_PIR_KEYWORDS	gtp-binding	RT		3	5.6E-1	1.0E0
	GOTERM_MF_FAT	GTP binding	RT		3	7.2E-1	1.0E0
	GOTERM_MF_FAT	guanyl ribonucleotide binding	RT		3	7.3E-1	1.0E0
	GOTERM_MF_FAT	guanyl nucleotide binding	RT		3	7.3E-1	1.0E0
	Annotation Cluster 6	Enrichment Score: 0.13	G		Count	P_Value	Benjamini

						Count	P_Value	Benjamini
☐	UP_SEQ_FEATURE	transmembrane region	RT			7	3.3E-1	1.0E0
☐	SP_PIR_KEYWORDS	membrane	RT			10	7.3E-1	1.0E0
☐	SP_PIR_KEYWORDS	transmembrane	RT			9	9.2E-1	1.0E0
☐	GOTERM_CC_FAT	intrinsic to membrane	RT			11	1.0E0	1.0E0
☐	GOTERM_CC_FAT	integral to membrane	RT			10	1.0E0	1.0E0
	Annotation Cluster 7	Enrichment Score: 0.08	G			Count	P_Value	Benjamini
☐	SP_PIR_KEYWORDS	nucleus	RT			8	7.1E-1	1.0E0
☐	GOTERM_BP_FAT	transcription	RT			3	7.6E-1	1.0E0
☐	GOTERM_BP_FAT	regulation of transcription, DNA-dependent	RT			4	8.2E-1	1.0E0
☐	GOTERM_BP_FAT	regulation of RNA metabolic process	RT			4	8.2E-1	1.0E0
☐	GOTERM_BP_FAT	regulation of transcription	RT			5	8.4E-1	1.0E0
☐	SP_PIR_KEYWORDS	dna-binding	RT			4	8.5E-1	1.0E0
☐	GOTERM_MF_FAT	transcription regulator activity	RT			5	8.8E-1	1.0E0
☐	GOTERM_MF_FAT	DNA binding	RT			6	9.3E-1	1.0E0
☐	GOTERM_MF_FAT	transcription factor activity	RT			3	9.5E-1	1.0E0

24 terms were not clustered.

Please cite *Nature Protocols 2009; 4(1):44 & Genome Biology 2003; 4(5):P3* within any publication that makes use of any methods inspired by **DAVID**.

SAIC SAIC Frederick A Division of Science Applications International Corporation An Employee-Owned Company NIAID ABCC NCI-Frederick National Institutes of Health FIRSTGOV Your First Click to the U.S. Government Department of Health and Human Services

Term of Service | Contact Us | Site Map
