# Supplementary material for: Natural Variation in Fish Transcriptomes: Comparative Analysis of the Fathead Minnow (Pimephales promelas) and Zebrafish (Danio rerio)
Source: PLoS One. 2014 Dec 10;9(12):e114178. doi: 10.1371/journal.pone.0114178 (PMC4262388; doi:10.1371/journal.pone.0114178)
Supplement: S5 Table — The top 50 most variable fathead minnow (PPR) genes based on average within-batch coefficient of variation (CV) under the Experiment factor. (DOCX) [file pone.0114178.s005.docx]

Table S5. The top 50 most variable fathead minnow (PPR) genes based on average within-batch coefficient of variation (CV) under the Experiment factor.

| PPR probe | intensity | CV | NCBI accession | annotation |
| --- | --- | --- | --- | --- |
| UF_Ppr_AF_100811 | 5.75 | 0.379 | XP_001334154 | PREDICTED: uncharacterized protein LOC796591 [Danio rerio] |
| UF_Ppr_AF_112725 | 7.65 | 0.370 | XP_002666796 | PREDICTED: nuclear mitotic apparatus protein 1-like, partial [Danio rerio] |
| UF_Ppr_AF_119024 | 4.69 | 0.357 | AAH62869 | Serpina1 protein, partial [Danio rerio] |
| UF_Ppr_AF_109653 | 7.02 | 0.356 | NP_001134796 | Gem-associated protein 6 [Salmo salar] |
| UF_Ppr_AF_102654 | 7.23 | 0.337 | ABG73405 | 14 kDa apolipoprotein [Pimephales promelas] |
| UF_Ppr_AF_106383 | 6.67 | 0.334 | ACI15889 | apolipoprotein A-I-1 [Hemibarbus mylodon] |
| UF_Ppr_AF_115272 | 7.99 | 0.334 | AAD23878 | vitellogenin precursor [Pimephales promelas] |
| UF_Ppr_AF_101666 | 9.48 | 0.329 | XP_002666953 | PREDICTED: dual oxidase maturation factor 2-like [Danio rerio] |
| UF_Ppr_AF_105571 | 6.56 | 0.319 | XP_001338767 | Zebrafish DNA sequence from clone DKEY-30I24 in linkage group 23, complete sequence |
| UF_Ppr_AF_103127 | 4.53 | 0.319 | XP_004542646 | Danio rerio muscleblind-like protein 1D (mbnl1d) mRNA, complete cds |
| UF_Ppr_AF_101059 | 10.14 | 0.311 | NP_001068583 | RWD domain-containing protein 2B [Danio rerio] |
| UF_Ppr_AF_110810 | 7.24 | 0.307 | XP_694694 | PREDICTED: NXPE family member 3-like [Danio rerio] |
| UF_Ppr_AF_117272 | 6.41 | 0.302 | XP_004555758 | PREDICTED: nucleoporin GLE1-like isoform X1 [Maylandia zebra] |
| UF_Ppr_AF_114060 | 8.40 | 0.297 | NP_001122235 | uncharacterized protein LOC571824 precursor [Danio rerio] |
| UF_Ppr_AF_106237 | 8.85 | 0.291 | NP_001012514 | mitochondrial-processing peptidase subunit beta [Danio rerio] |
| UF_Ppr_AF_101877 | 6.52 | 0.286 | NP_570995 | retinol-binding protein 4 precursor [Danio rerio] |
| UF_Ppr_AF_107384 | 9.24 | 0.284 | CAI11513 | Danio rerio small integral membrane protein 8 (smim8), transcript variant 1, mRNA |
| UF_Ppr_AF_103711 | 8.10 | 0.278 | NP_956139 | glutamate--cysteine ligase regulatory subunit [Danio rerio] |
| UF_Ppr_AF_108259 | 8.22 | 0.276 | NP_001138261 | DDB1- and CUL4-associated factor 17 [Danio rerio] |
| UF_Ppr_AF_115478 | 7.34 | 0.274 | AAG30407 | vitellogenin 3 precursor, partial [Danio rerio] |
| UF_Ppr_AF_105833 | 9.25 | 0.271 | NP_001074122 | 2-oxoisovalerate dehydrogenase subunit beta, mitochondrial [Danio rerio] |
| UF_Ppr_AF_104300 | 9.78 | 0.270 | NP_001133932 | Kynurenine--oxoglutarate transaminase 3 [Salmo salar] |
| UF_Ppr_AF_117336 | 6.65 | 0.257 | XP_003200815 | PREDICTED: meiosis inhibitor protein 1-like [Danio rerio] |
| UF_Ppr_AF_118265 | 7.17 | 0.255 | AAM28949 | band 3 anion exchange protein [Danio rerio] |
| UF_Ppr_AF_111076 | 7.56 | 0.253 | NP_001002516 | nucleoside diphosphate kinase homolog 5 [Danio rerio] |
| UF_Ppr_AF_106169 | 9.76 | 0.251 | XP_003199893 | PREDICTED: ES1 protein homolog, mitochondrial isoform 1 [Danio rerio] |
| UF_Ppr_AF_117725 | 7.57 | 0.247 | XP_002591736 | hypothetical protein BRAFLDRAFT_58953 [Branchiostoma floridae] |
| UF_Ppr_AF_102309 | 6.23 | 0.244 | Q32LQ4 | Danio rerio betaine-homocysteine methyltransferase, mRNA |
| UF_Ppr_AF_113291 | 8.87 | 0.242 | AAH63960 | Sepp1a protein [Danio rerio] |
| UF_Ppr_AF_105225 | 7.10 | 0.239 | NP_001153301 | proton myo-inositol cotransporter [Danio rerio] |
| UF_Ppr_AF_110121 | 6.06 | 0.238 | AEM60431 | warm-temperature-acclimation-associated 65-kDa protein [Misgurnus mizolepis] |
| UF_Ppr_AF_103938 | 6.58 | 0.236 | NP_001077337 | interleukin-10 receptor subunit beta precursor [Danio rerio] |
| UF_Ppr_AF_109272 | 6.80 | 0.235 | AAH95829 | LOC553487 protein, partial [Danio rerio] |
| UF_Ppr_AF_110598 | 8.53 | 0.234 | AAR20998 | neurotoxin/C59/Ly-6-like protein [Ctenopharyngodon idella] |
| UF_Ppr_AF_108178 | 7.90 | 0.230 | CAA71473 | Pimephales promelas isolate KU T7991 cytochrome b (cytb) gene, partial cds; mitochondrial |
| UF_Ppr_AF_103323 | 8.74 | 0.228 | NP_001231965 | uncharacterized protein LOC571696 [Danio rerio] |
| UF_Ppr_AF_108524 | 6.47 | 0.227 | XP_003199751 | Zebrafish DNA sequence from clone DKEY-22O22 in linkage group 16, complete sequence |
| UF_Ppr_AF_108512 | 10.03 | 0.227 | CAF90035 | PREDICTED: Oryzias latipes patatin-like phospholipase domain-containing protein 7-like (LOC101172993), mRNA |
| UF_Ppr_AF_106177 | 11.40 | 0.227 | XP_001334671 | PREDICTED: Danio rerio ornithine carbamoyltransferase (otc), mRNA |
| UF_Ppr_AF_106875 | 8.35 | 0.226 | XP_001338088 | PREDICTED: tumor necrosis factor receptor superfamily member 14-like isoform X1 [Danio rerio] |
| UF_Ppr_AF_111408 | 4.57 | 0.225 | NP_571970 | glutamate receptor, ionotropic, AMPA 2b precursor [Danio rerio] |
| UF_Ppr_AF_103380 | 6.20 | 0.224 | XP_696970 | PREDICTED: probable polypeptide N-acetylgalactosaminyltransferase 8 [Danio rerio] |
| UF_Ppr_AF_103963 | 8.41 | 0.223 | XP_004084377 | PREDICTED: uncharacterized protein LOC101162891 [Oryzias latipes] |
| UF_Ppr_AF_118788 | 11.42 | 0.222 | XP_003201020 | PREDICTED: uncharacterized protein LOC100034566 [Danio rerio] |
| UF_Ppr_AF_101602 | 6.22 | 0.222 | NP_001071011 | tRNA wybutosine-synthesizing protein 5 [Danio rerio] |
| UF_Ppr_AF_104344 | 8.03 | 0.222 | ACI34223 | Succinate-semialdehyde dehydrogenase, mitochondrial precursor [Salmo salar] |
| UF_Ppr_AF_115428 | 10.28 | 0.221 | XP_003201606 | NA |
| UF_Ppr_AF_104962 | 7.88 | 0.221 | XP_003455661 | Danio rerio protein tyrosine kinase 2 beta, b (ptk2bb), mRNA |
| UF_Ppr_AF_116834 | 6.16 | 0.220 | BX950855 | Zebrafish sequence from clone DKEY-166K12 in linkage group 19 |
| UF_Ppr_AF_110307 | 7.60 | 0.219 | NP_957116 | Danio rerio G protein-coupled receptor 143 (gpr143), mRNA |
