# Supplementary material for: Natural Variation in Fish Transcriptomes: Comparative Analysis of the Fathead Minnow (Pimephales promelas) and Zebrafish (Danio rerio)
Source: PLoS One. 2014 Dec 10;9(12):e114178. doi: 10.1371/journal.pone.0114178 (PMC4262388; doi:10.1371/journal.pone.0114178)
Supplement: S6 Table — The top 50 most variable zebrafish (DRE) genes based on average within-batch coefficient of variation (CV) under the Experiment factor. (DOCX) [file pone.0114178.s006.docx]

Table S6. The top 50 most variable zebrafish (DRE) genes based on average within-batch coefficient of variation (CV) under the Experiment factor.

| DRE probe | intensity | CV | NCBI accession | annotation |
| --- | --- | --- | --- | --- |
| A_15_P106744 | 9.06 | 0.443 | BC044517 | ctsba cathepsin B, a [ Danio rerio] |
| A_15_P100684 | 6.85 | 0.411 | zgc:162235 | dnai2a dynein, axonemal, intermediate chain 2a [ Danio rerio] |
| A_15_P103566 | 7.65 | 0.390 |  | NA |
| A_15_P104395 | 12.20 | 0.389 | NM_001017593 | Danio rerio epithelial cell adhesion molecule (epcam), mRNA |
| A_15_P104853 | 9.99 | 0.365 | NM_001122610 | Danio rerio vitellogenin 6 (vtg6), mRNA |
| A_15_P104537 | 9.42 | 0.356 | NM_001044913 | Danio rerio vitellogenin 2 (vtg2), transcript variant 1, mRNA |
| A_15_P103918 | 10.07 | 0.354 |  | NA |
| A_15_P104585 | 7.03 | 0.354 |  | NA |
| A_15_P117945 | 9.87 | 0.349 |  | Vitellogenin 1 - Danio rerio, partial (28%) [TC423222] |
| A_15_P110757 | 8.05 | 0.349 | NM_001045294 | Danio rerio vitellogenin 4 (vtg4), mRNA |
| A_15_P117501 | 7.54 | 0.347 |  | vitellogenin 6 [Source:ZFIN;Acc:ZDB-GENE-001201-5] [ENSDART00000078231] |
| A_15_P100969 | 9.80 | 0.347 |  | Vitellogenin 1 - Danio rerio, partial (13%) [TC435210] |
| A_15_P121286 | 9.32 | 0.345 |  | NA |
| A_15_P102713 | 10.88 | 0.340 | NM_001122610 | Danio rerio vitellogenin 6 (vtg6), mRNA |
| A_15_P108258 | 7.29 | 0.338 |  | NA |
| A_15_P101703 | 8.42 | 0.334 | BC066516 | Danio rerio zgc:77041, mRNA (cDNA clone MGC:77041 IMAGE:6959764) |
| A_15_P102894 | 7.34 | 0.332 |  | PREDICTED: vitellogenin 3, phosvitinless - Danio rerio, complete [TC366015] |
| A_15_P116918 | 10.49 | 0.328 |  | NA |
| A_15_P100702 | 11.28 | 0.328 |  | NA |
| A_15_P117632 | 10.60 | 0.325 | NM_001025189 | Danio rerio vitellogenin 5 (vtg5), mRNA |
| A_15_P109536 | 9.50 | 0.322 | AF254638 | Danio rerio vitellogenin 3 precursor (vg3) mRNA, partial cds. |
| A_15_P101102 | 6.85 | 0.322 | AF527755 | Danio rerio Cyp11a1 mRNA, complete cds. |
| A_15_P119933 | 8.44 | 0.317 | Zgc:114012 | vtg5 vitellogenin 5 [ Danio rerio (zebrafish) ] |
| A_15_P111277 | 10.70 | 0.317 |  | NA |
| A_15_P116927 | 11.04 | 0.317 |  | NA |
| A_15_P104663 | 10.31 | 0.316 |  | Vitellogenin 1 - Danio rerio, partial (10%) [TC456802] |
| A_15_P114997 | 11.19 | 0.316 | NM_001044913 | Danio rerio vitellogenin 2 (vtg2), transcript variant 1, mRNA |
| A_15_P109260 | 9.98 | 0.314 |  | Vitellogenin 1 - Danio rerio, partial (15%) [TC407817] |
| A_15_P108378 | 9.40 | 0.313 | NM_200504 | Danio rerio PRP31 pre-mRNA processing factor 31 homolog (yeast) (prpf31), mRNA |
| A_15_P102452 | 10.79 | 0.307 |  | NA |
| A_15_P103847 | 11.53 | 0.307 | NM_001045294 | Danio rerio vitellogenin 4 (vtg4), mRNA |
| A_15_P109183 | 7.46 | 0.307 | BC134930 | Danio rerio zgc:162235, mRNA (cDNA clone MGC:162235 IMAGE:8732839), complete cds |
| A_15_P114868 | 10.76 | 0.307 |  | NA |
| A_15_P119670 | 7.00 | 0.303 | NM_152953 | Danio rerio cytochrome P450, subfamily XIA, polypeptide 1 (cyp11a1), mRNA |
| A_15_P100599 | 11.04 | 0.301 | Zgc:136383 | vtg4 vitellogenin 4 [ Danio rerio] |
| A_15_P110799 | 7.96 | 0.299 | NM_001130586 | Danio rerio apolipoprotein A-II (apoa2), mRNA |
| A_15_P109735 | 8.91 | 0.298 | NM_213301 | Danio rerio aldehyde dehydrogenase 2, tandem duplicate 2 (aldh2.2), mRNA |
| A_15_P119861 | 6.58 | 0.298 | BC108037 | ribonucleotide reductase M2 b [Source:ZFIN;Acc:ZDB-GENE-030616-614] [ENSDART00000050750] |
| A_15_P116003 | 8.91 | 0.297 |  | glutaredoxin (thioltransferase) [Source:ZFIN;Acc:ZDB-GENE-041010-11] [ENSDART00000134469] |
| A_15_P119630 | 11.86 | 0.296 | NM_001122610 | Danio rerio vitellogenin 6 (vtg6), mRNA [NM_001122610] |
| A_15_P100658 | 7.46 | 0.294 |  | Vitellogenin 1 - Danio rerio, partial (24%) [TC393044] |
| A_15_P106680 | 12.11 | 0.293 | NM_001044897 | Danio rerio vitellogenin 1 (vtg1), mRNA [NM_001044897] |
| A_15_P109234 | 6.41 | 0.292 | NM_001037236 | Danio rerio complement component c3c (c3c), mRNA [NM_001037236] |
| A_15_P106836 | 11.29 | 0.291 |  | NA |
| A_15_P110416 | 12.18 | 0.290 | NM_001044913 | Danio rerio vitellogenin 2 (vtg2), transcript variant 1, mRNA |
| A_15_P120732 | 7.08 | 0.290 |  | NA |
| A_15_P120675 | 6.47 | 0.289 | NM_001005390 | Danio rerio alkB, alkylation repair homolog 6 (E. coli) (alkbh6), mRNA |
| A_15_P108848 | 11.78 | 0.289 |  | Vitellogenin 1 - Danio rerio, partial (28%) [TC435753] |
| A_15_P109445 | 11.14 | 0.285 |  | Serpina1 protein - Danio rerio, partial (32%) [TC452102] |
| A_15_P109600 | 10.99 | 0.283 | BC100054 | complement component 7b [Source:HGNC Symbol;Acc:1346] [ENSDART00000132741] |
