# Supplementary figures and images for: Natural Variation in Fish Transcriptomes: Comparative Analysis of the Fathead Minnow (Pimephales promelas) and Zebrafish (Danio rerio)
Source: PLoS One. 2014 Dec 10;9(12):e114178. doi: 10.1371/journal.pone.0114178 (PMC4262388; doi:10.1371/journal.pone.0114178)

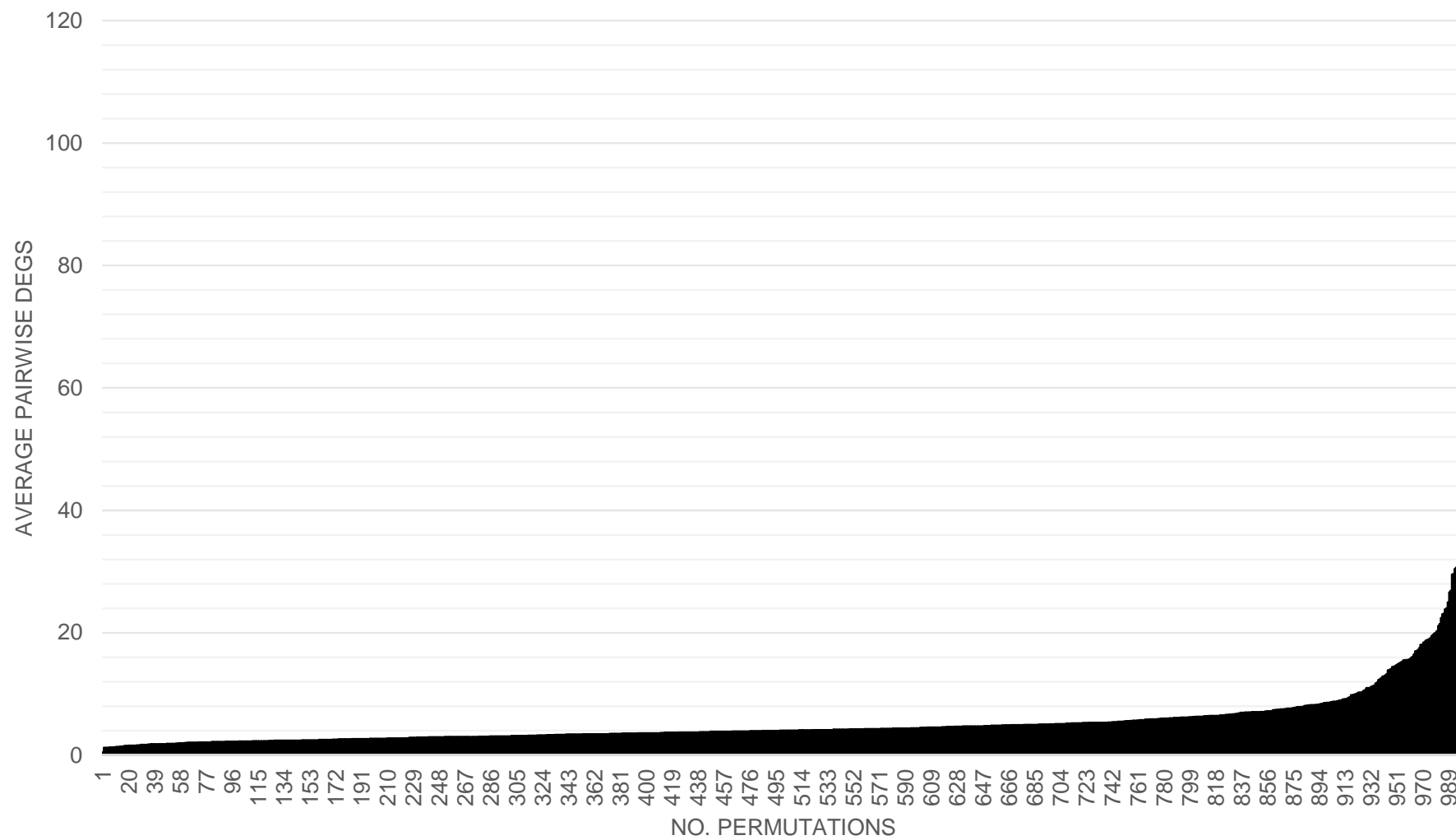

Figure S1A

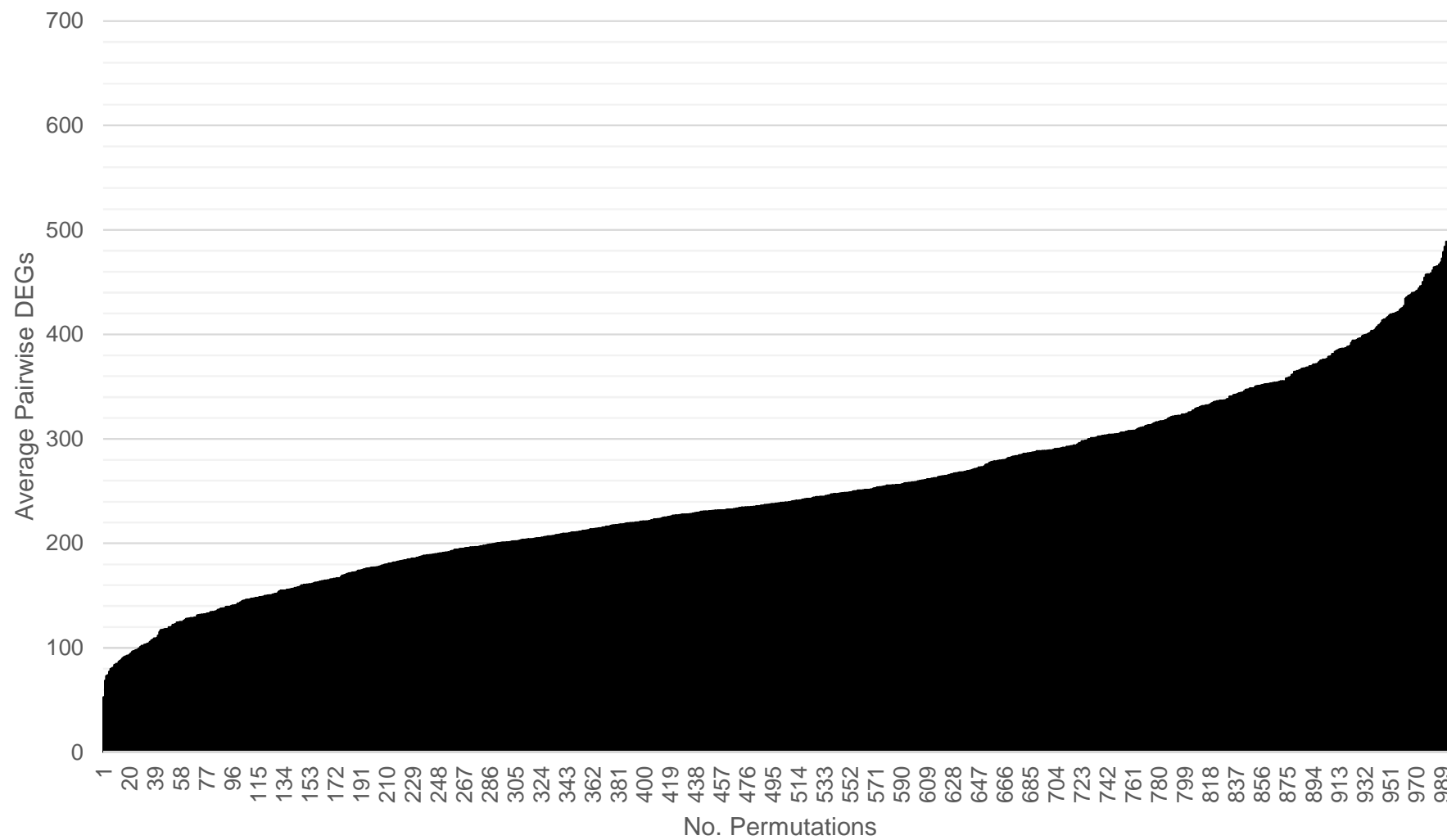

Figure S1B

Supplement: S1 File — Average number of DEGs per pair of between-batch comparison in 1000 permutations. Fathead minnow (Figure S1A) critical cutoffs were: 5%, 15; 1%, 27; 0.1%, 104. Zebrafish (Figure S1B) critical cutoffs were: 5%, 418; 1%, 479; 0.1%, 594. Samples were grouped by the factor Experiment. (PDF) [file pone.0114178.s007.pdf]

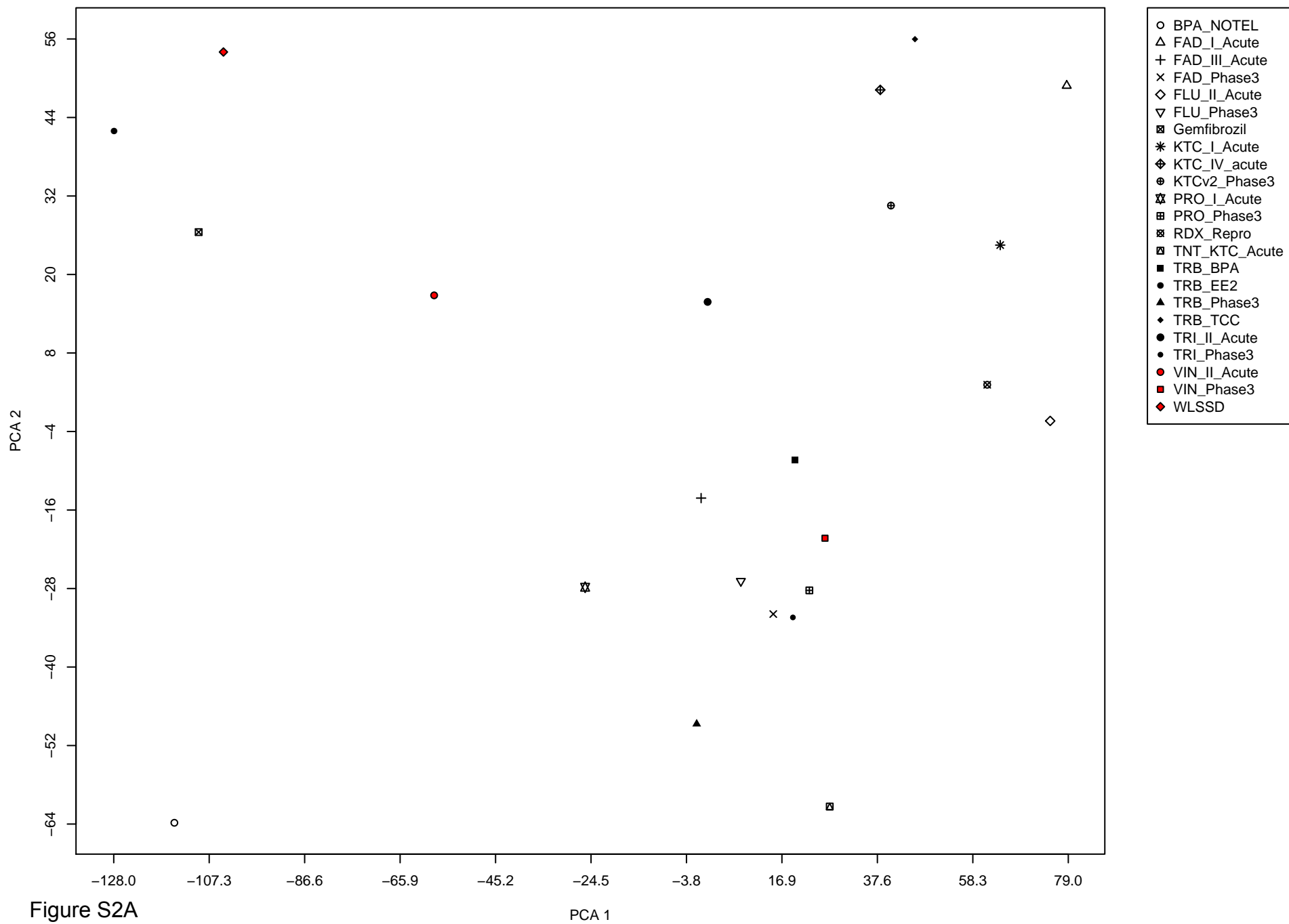

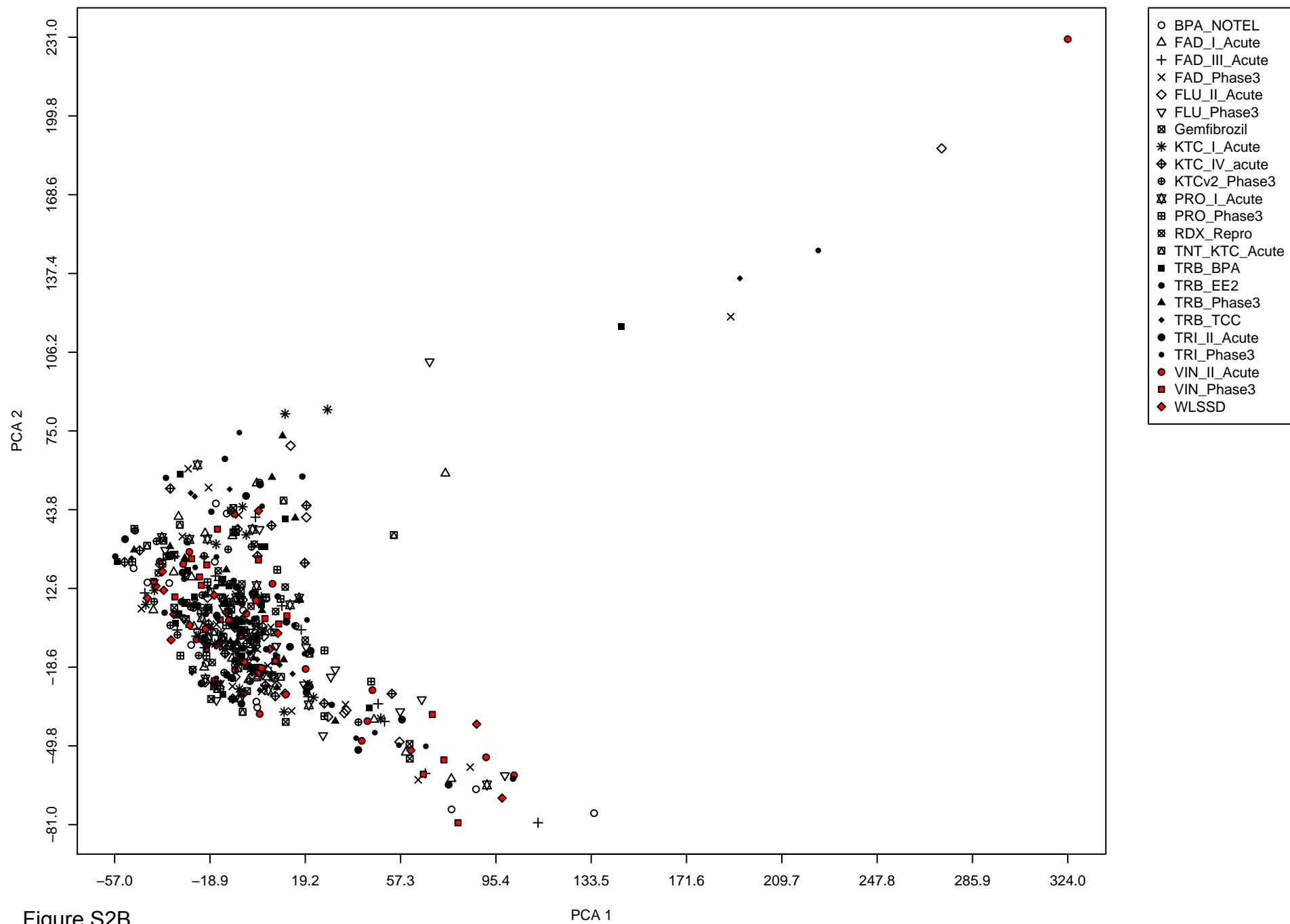

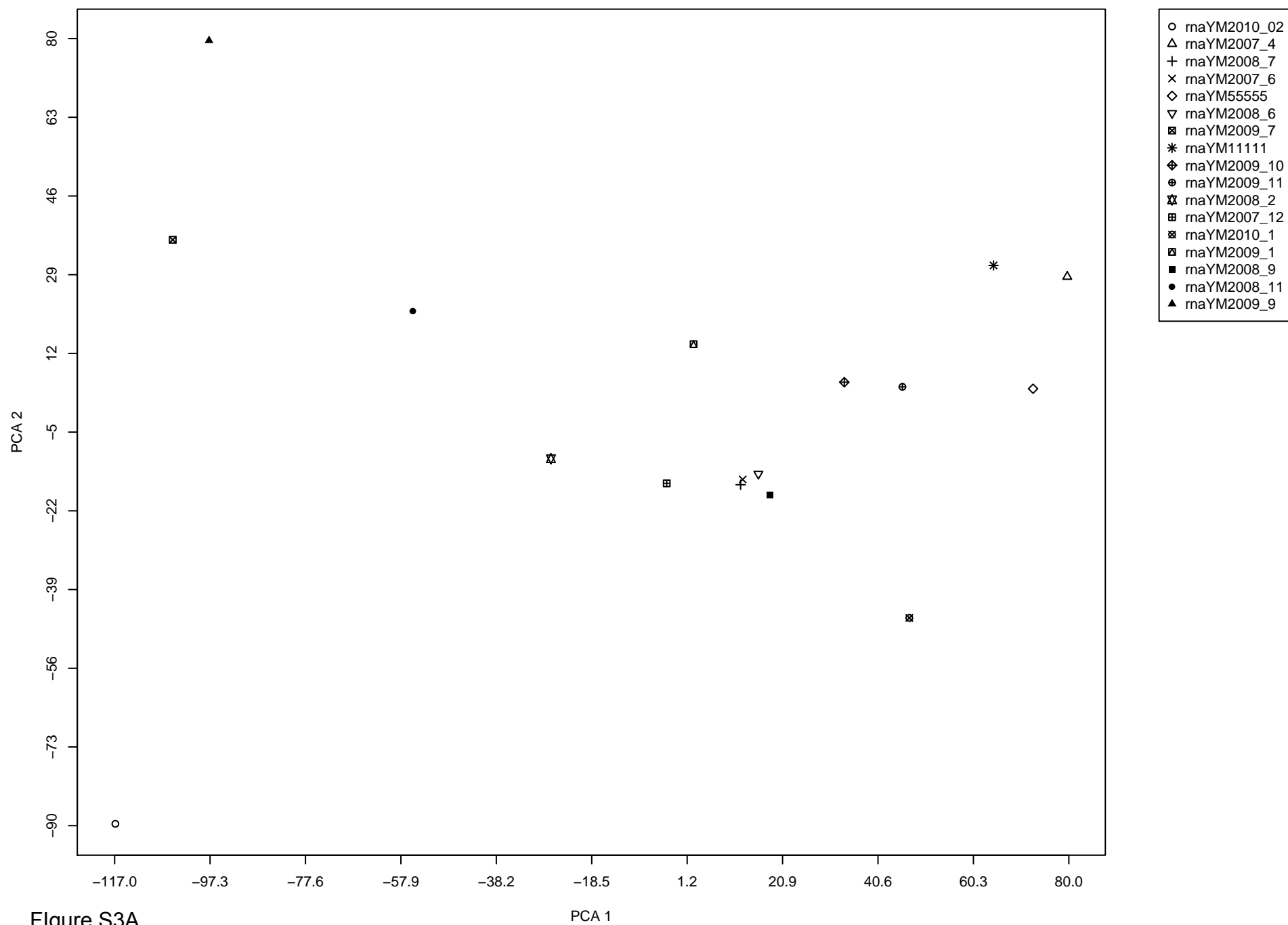

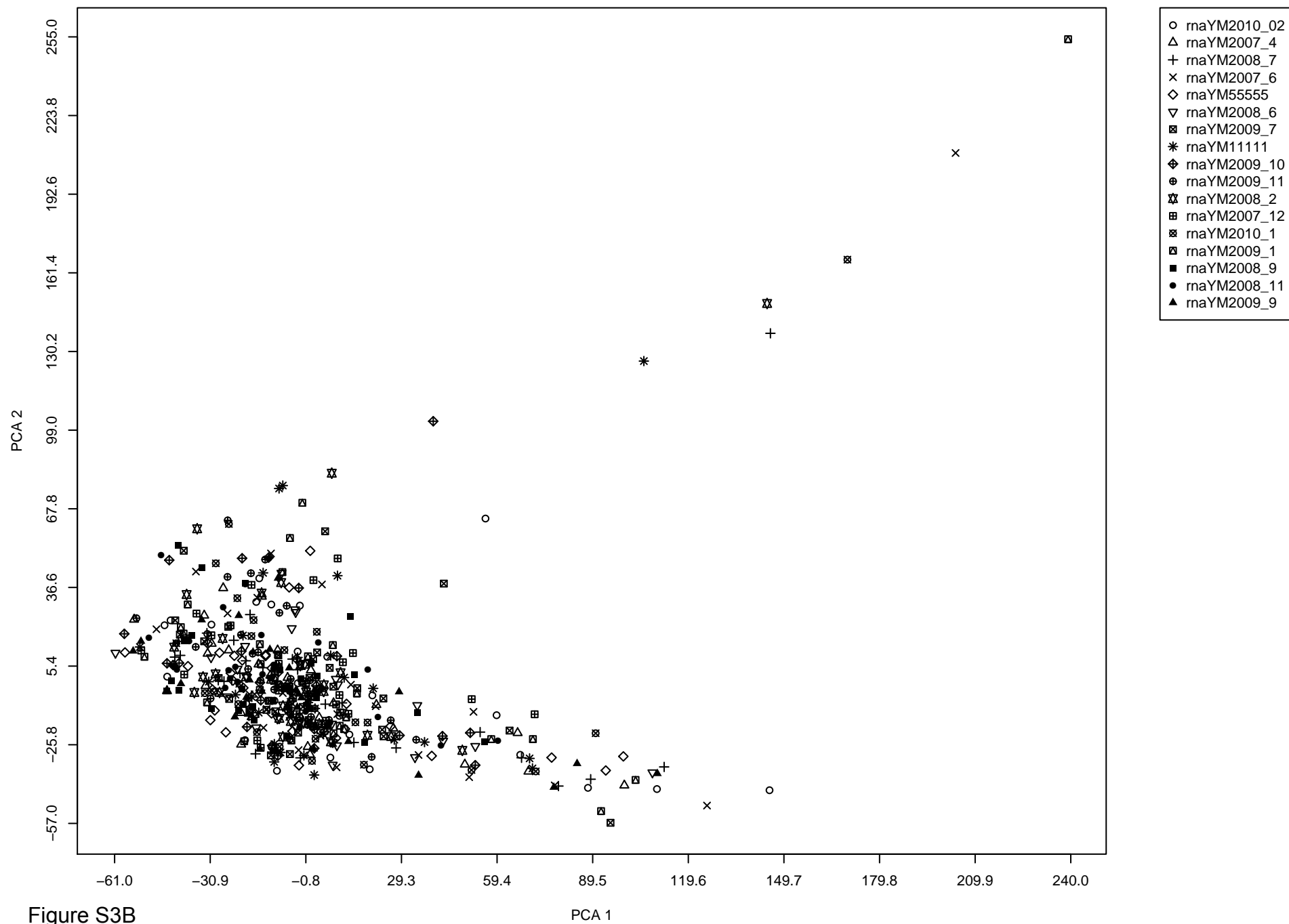

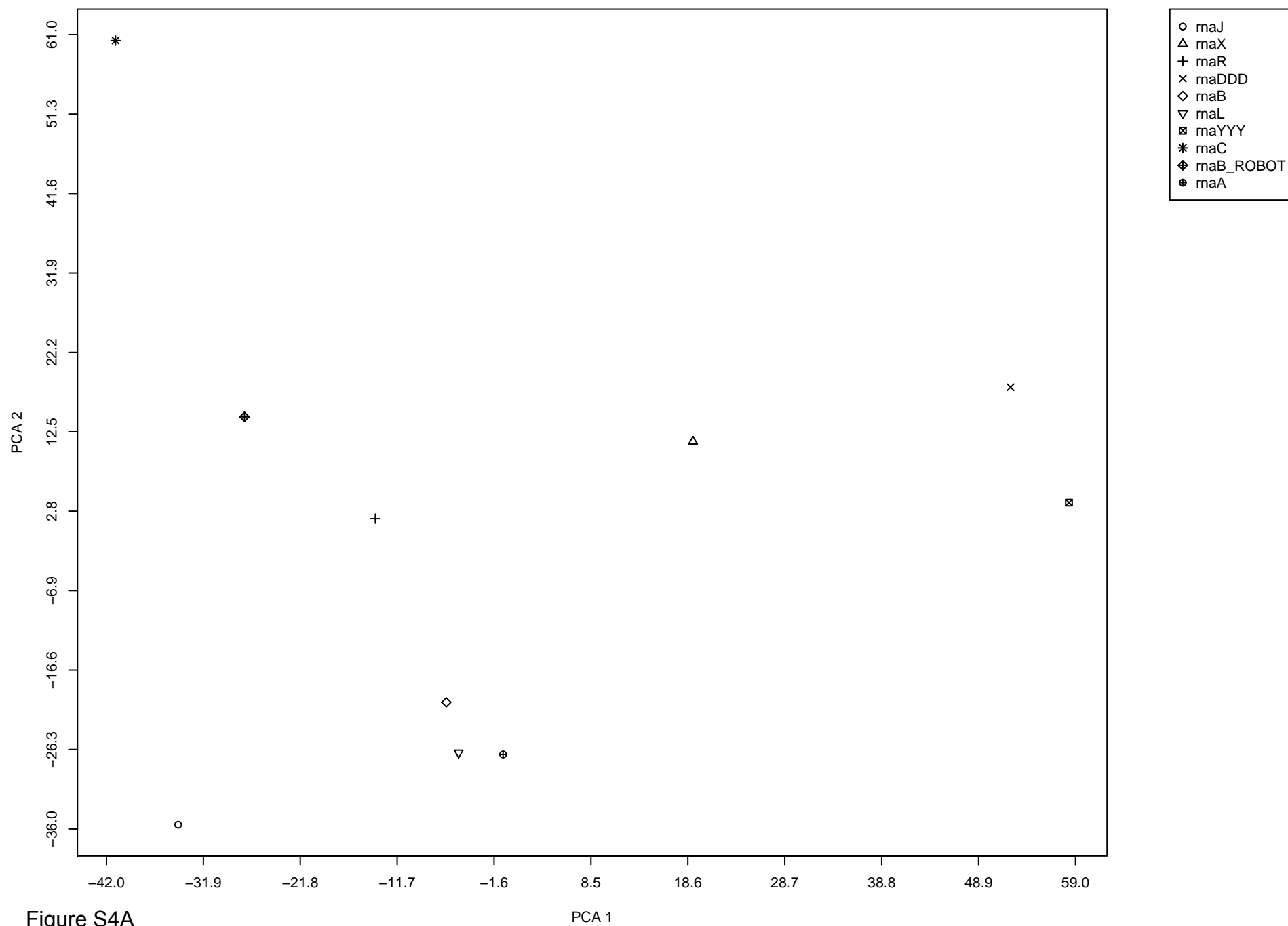

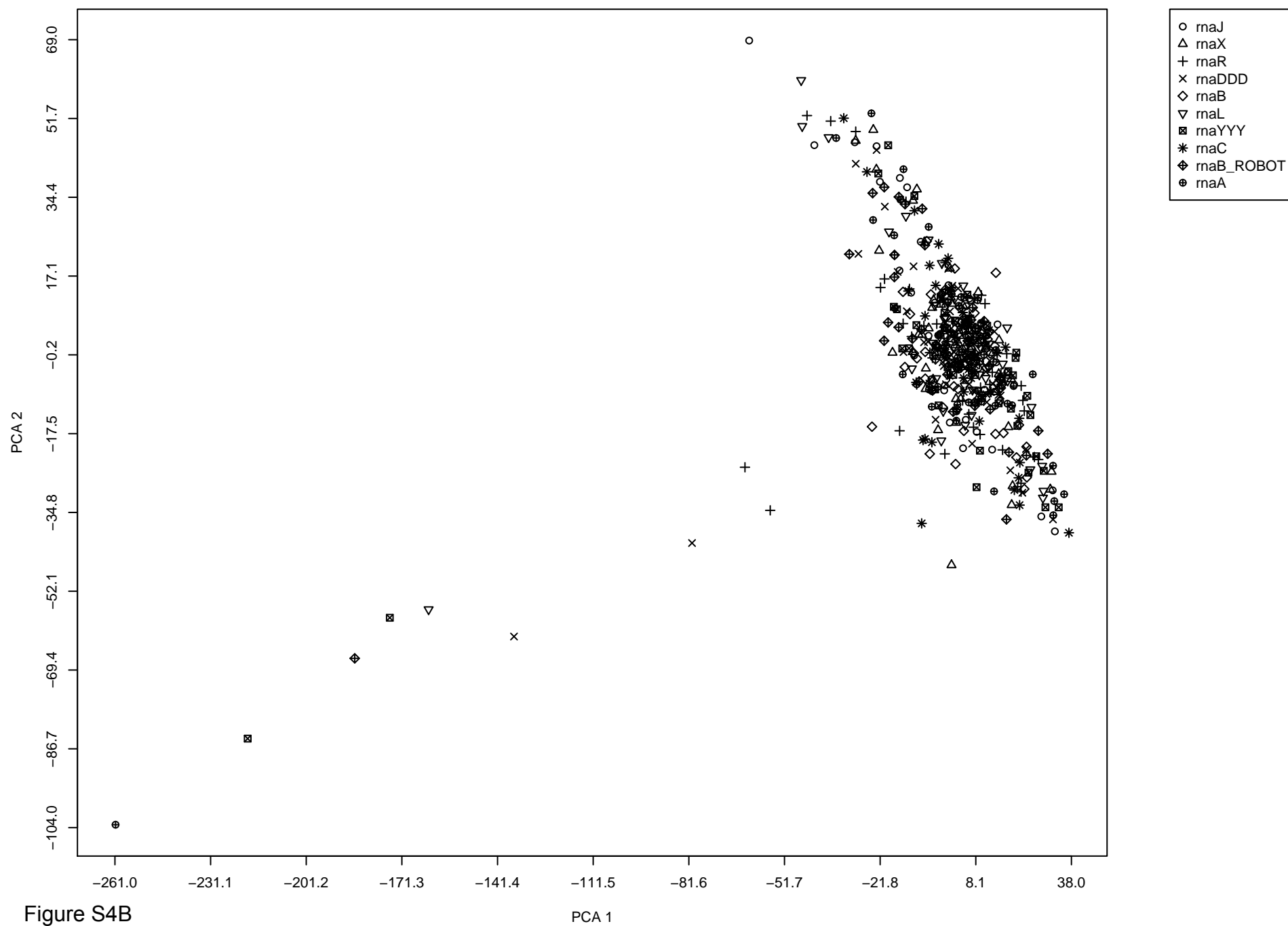

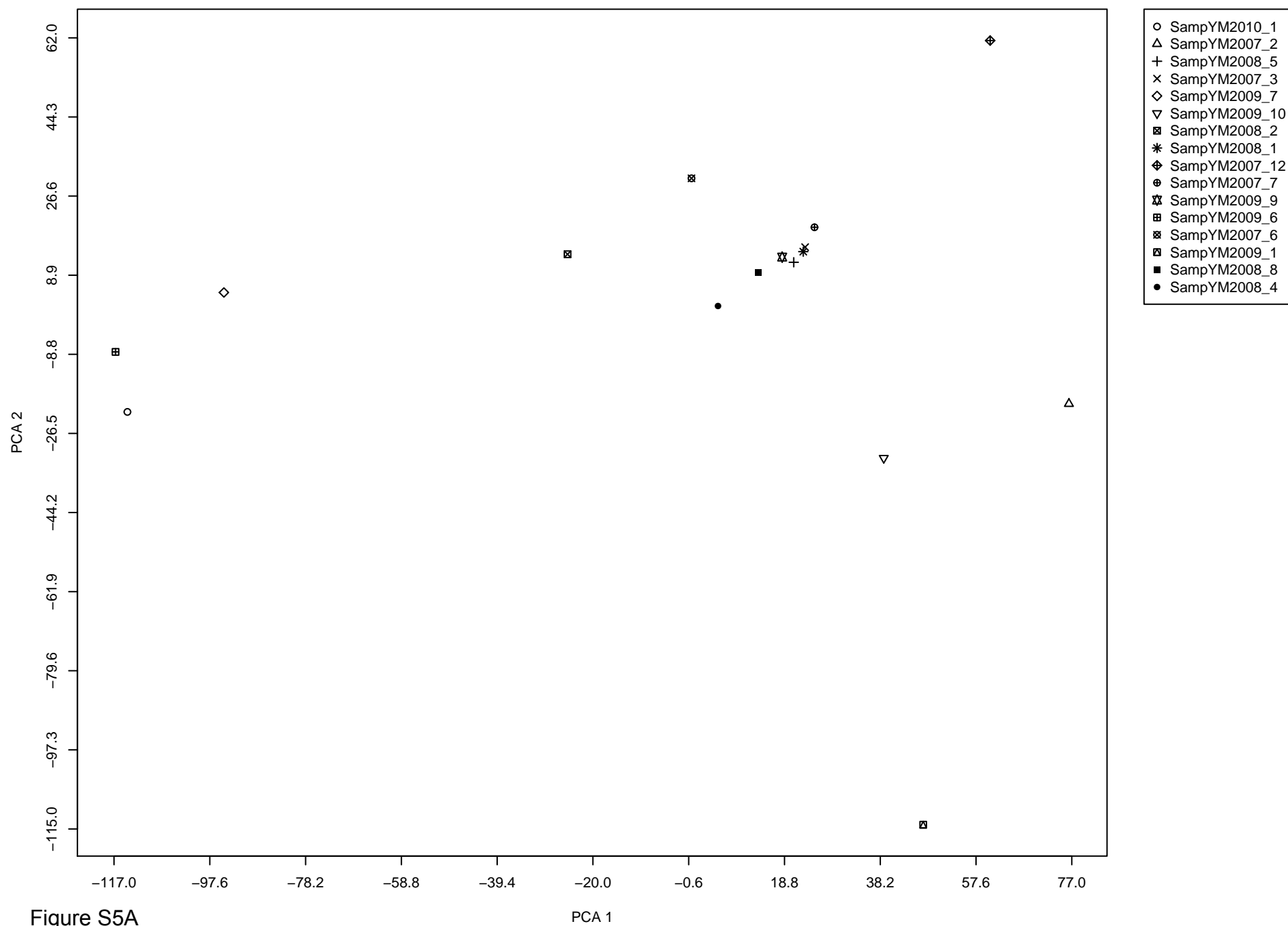

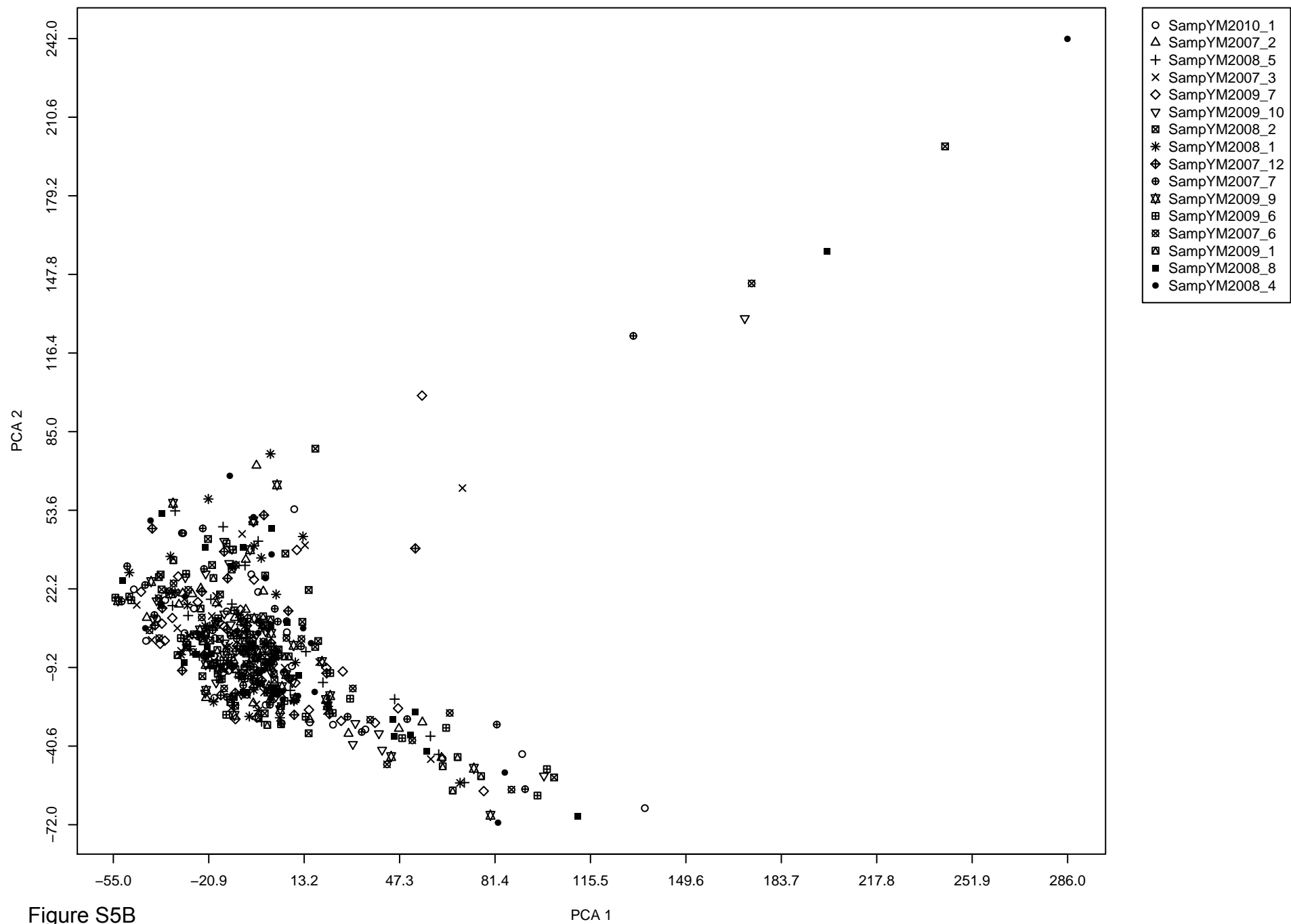

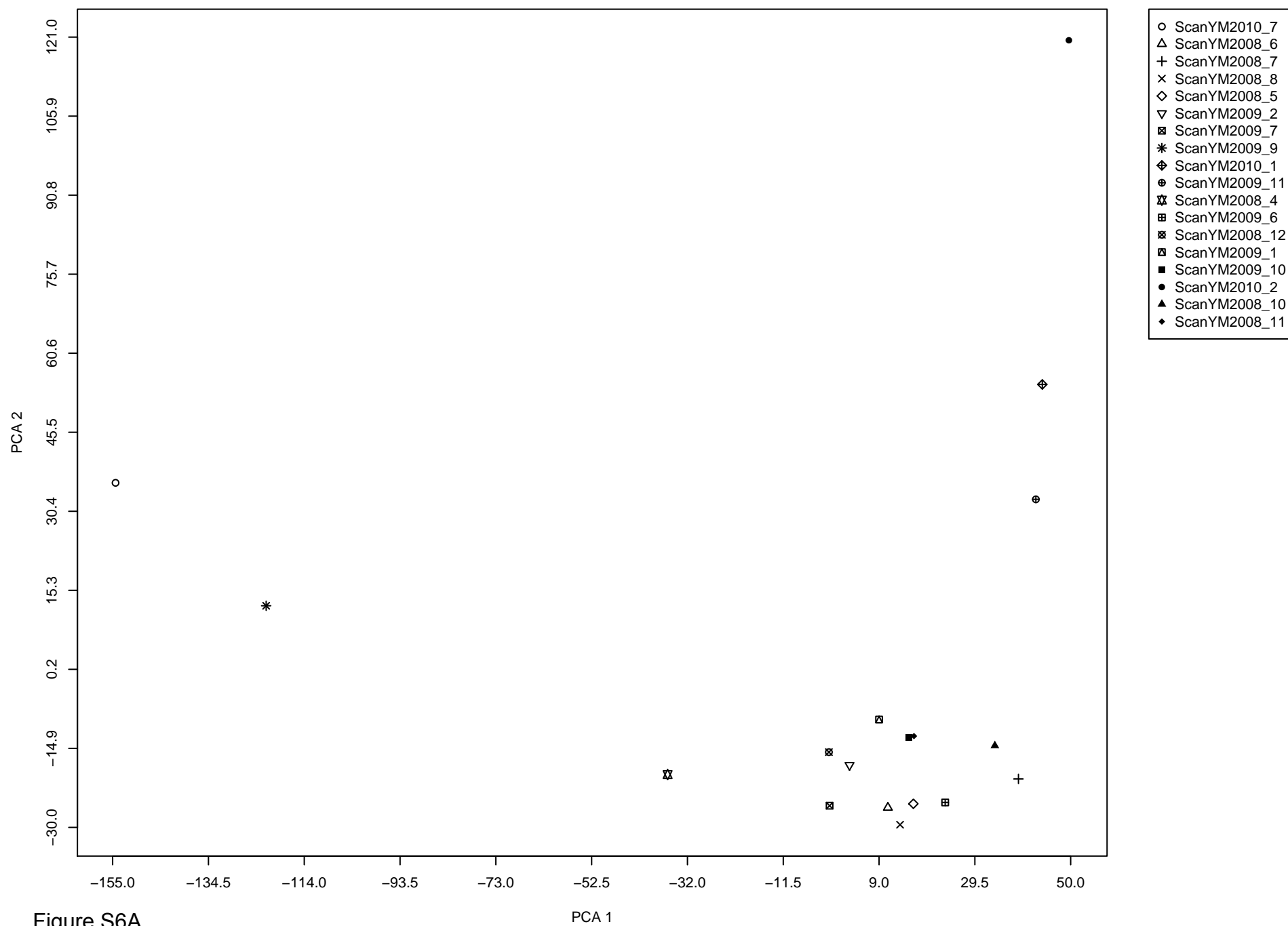

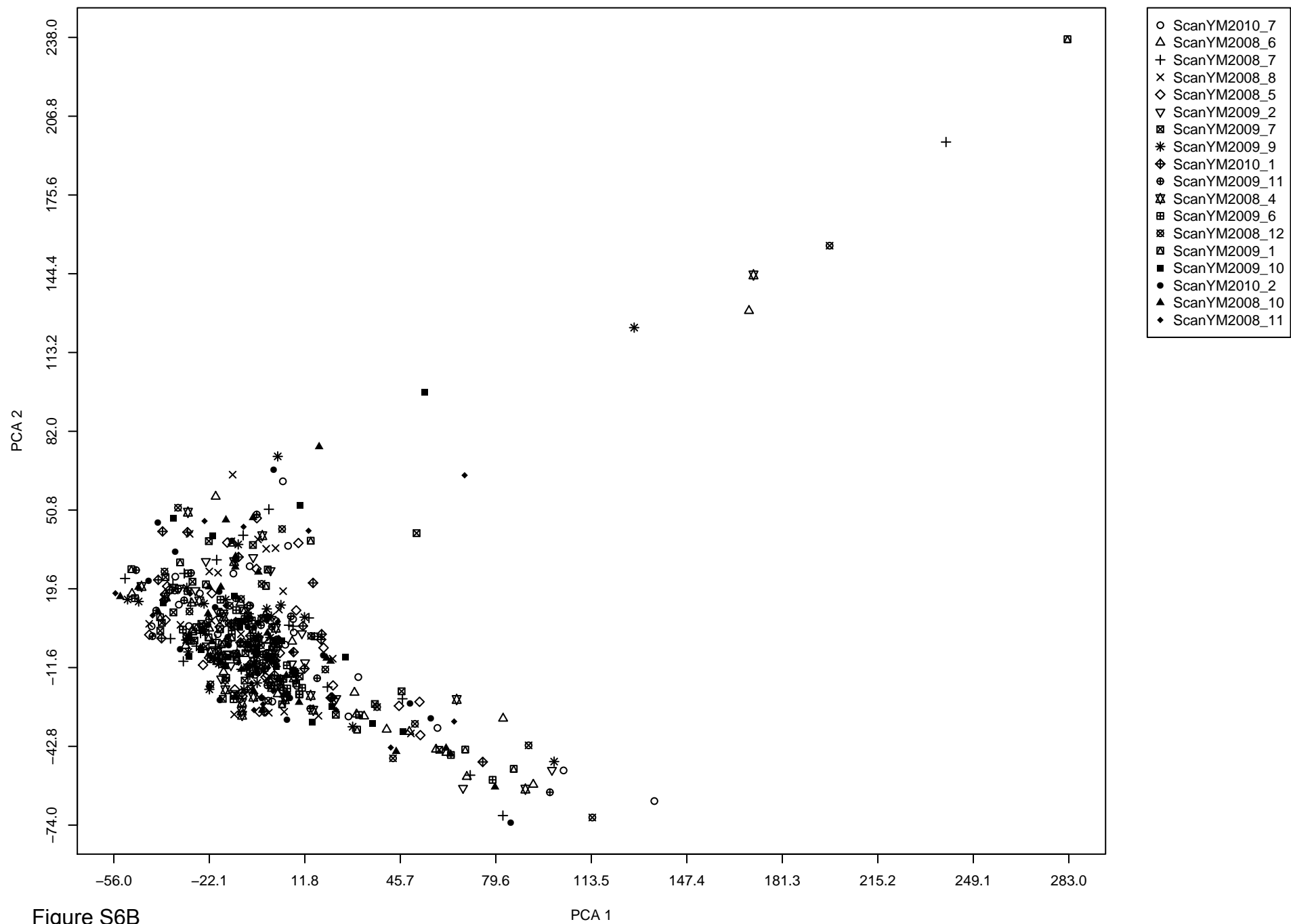

Supplement: S2 File — The PCA plots of fathead minnow samples based on all the DEGs identified as between-batch variation. Samples were grouped by Experiment (Figure S2A, S2B), RNA Date (Figure S3A, S3B), RNA Person (Figure S4A, S4B), Sampling Date (Figure S5A, S5B), and Scan Date (Figure S6A, S6B). Each figure was based on either the average gene intensity by individual batches (A) or the gene intensity of individual samples (B). DEGs were based on the simulated reference method. (PDF) [file pone.0114178.s008.pdf]

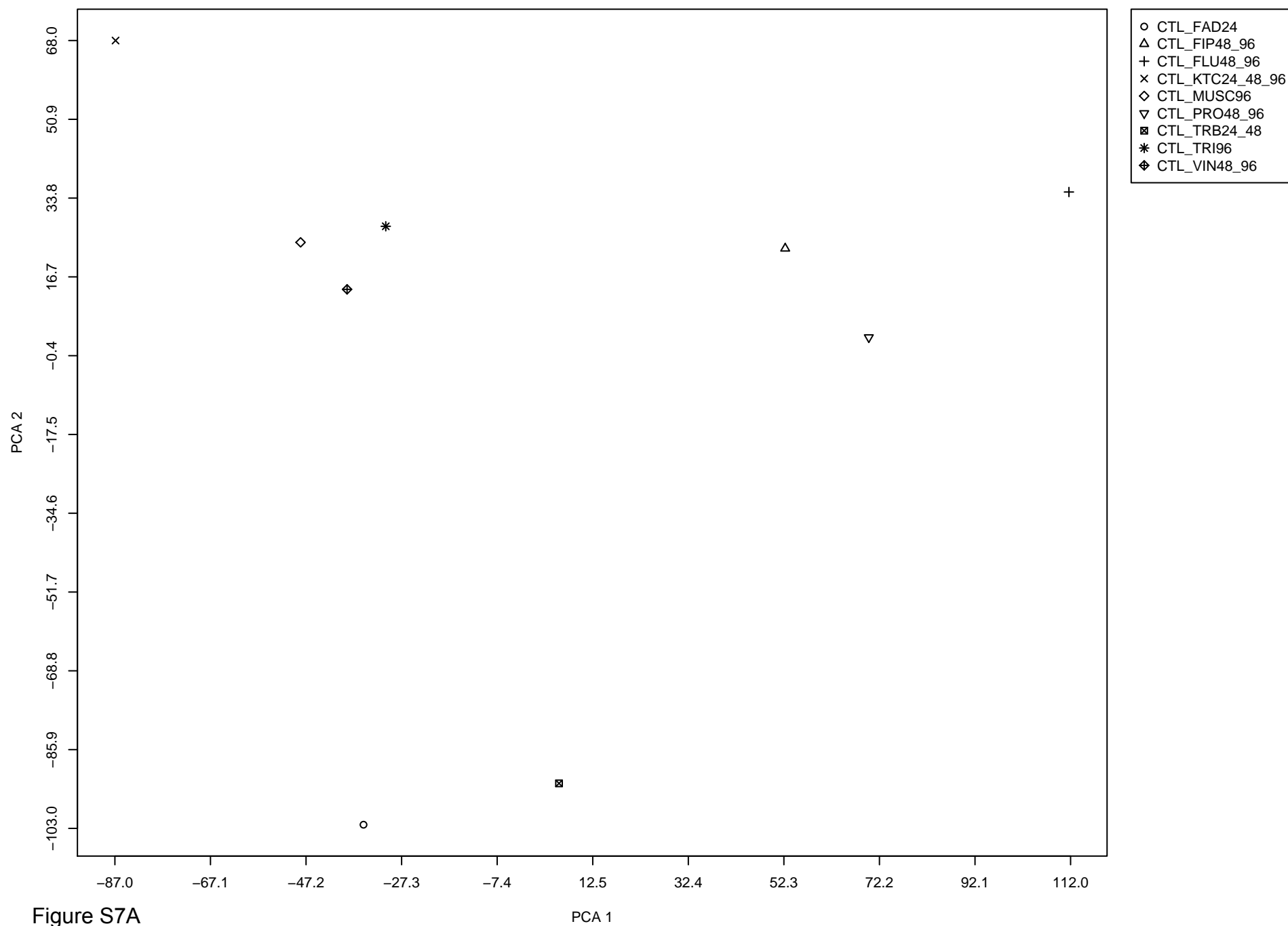

Figure S7A

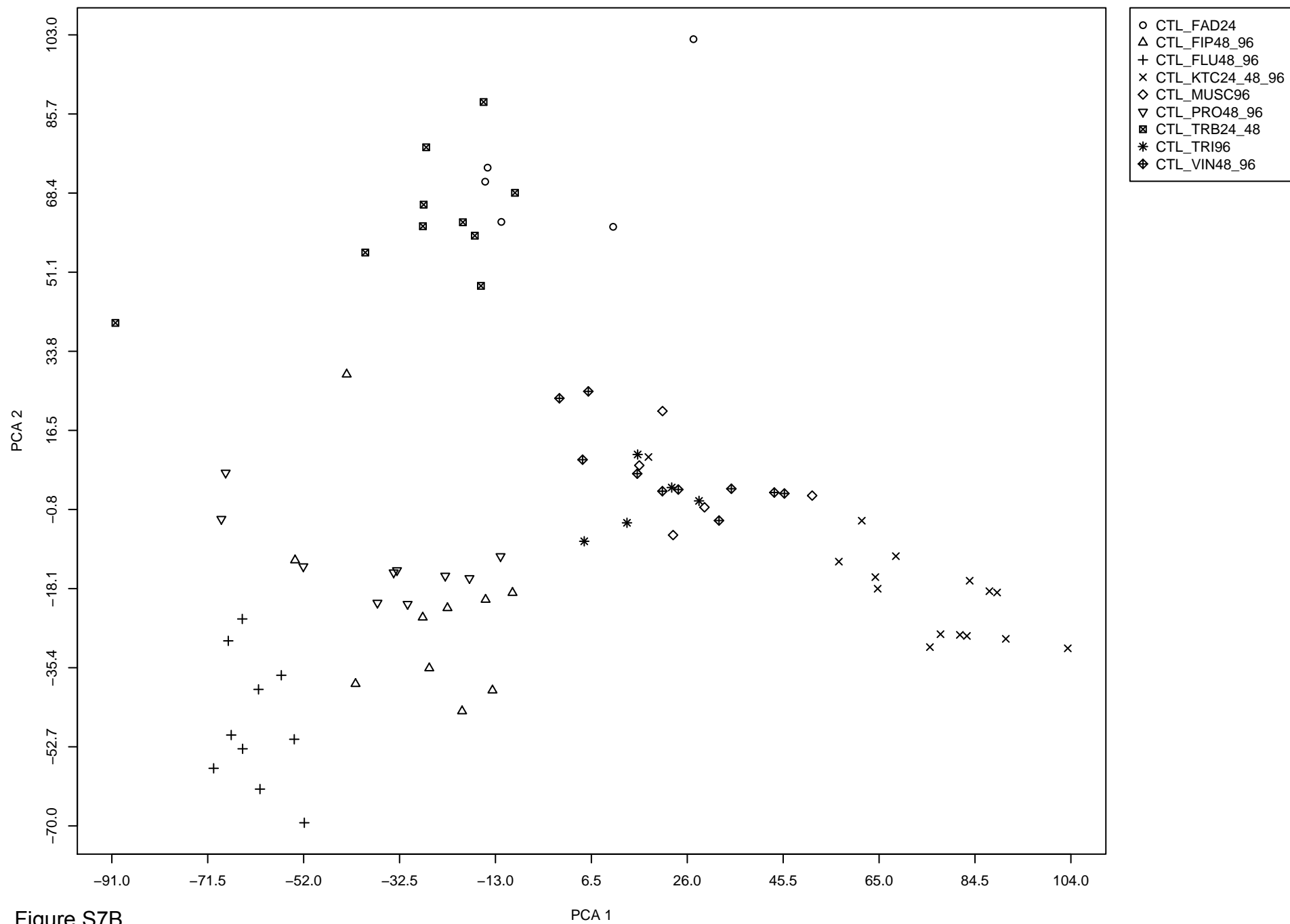

Figure S7B

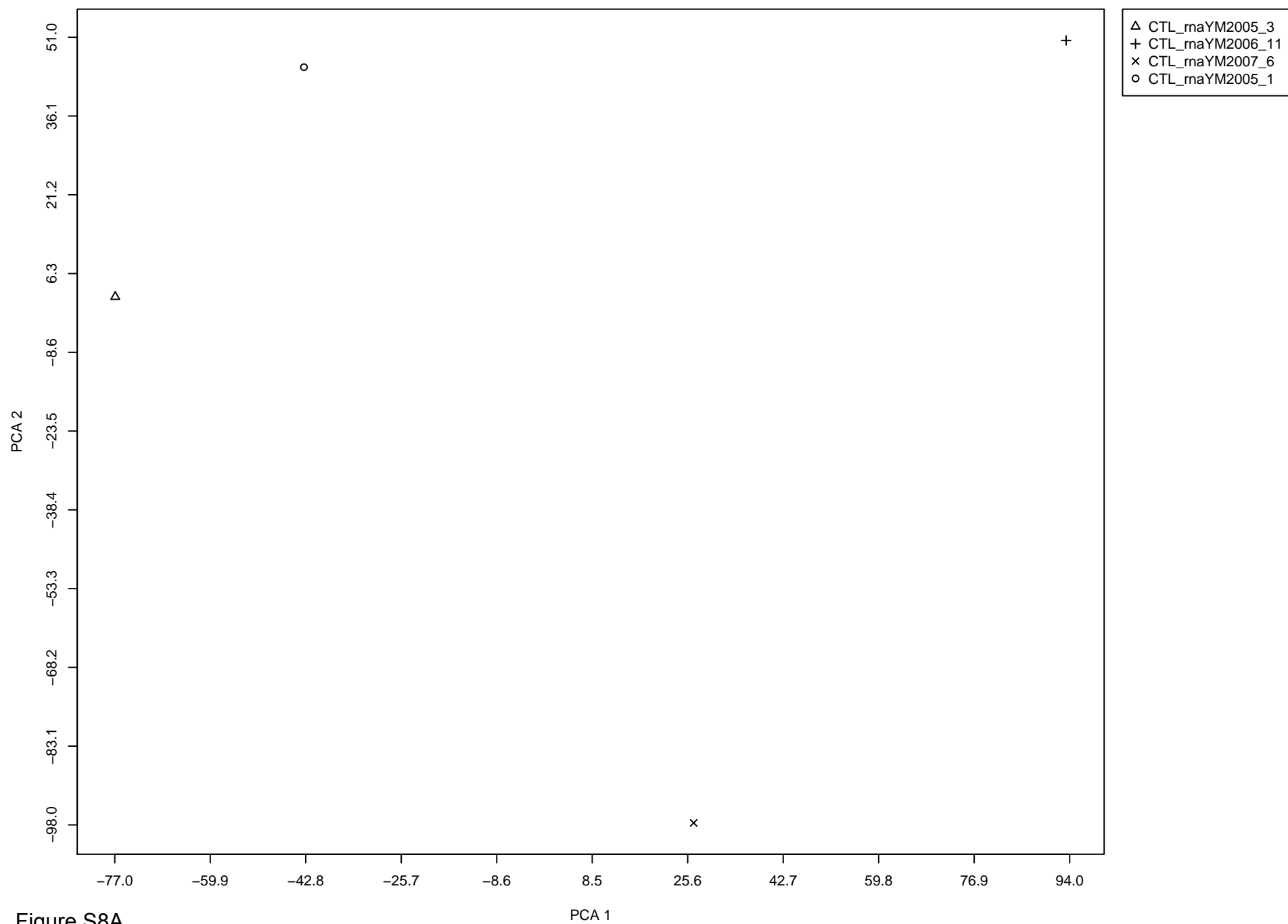

Figure S8A

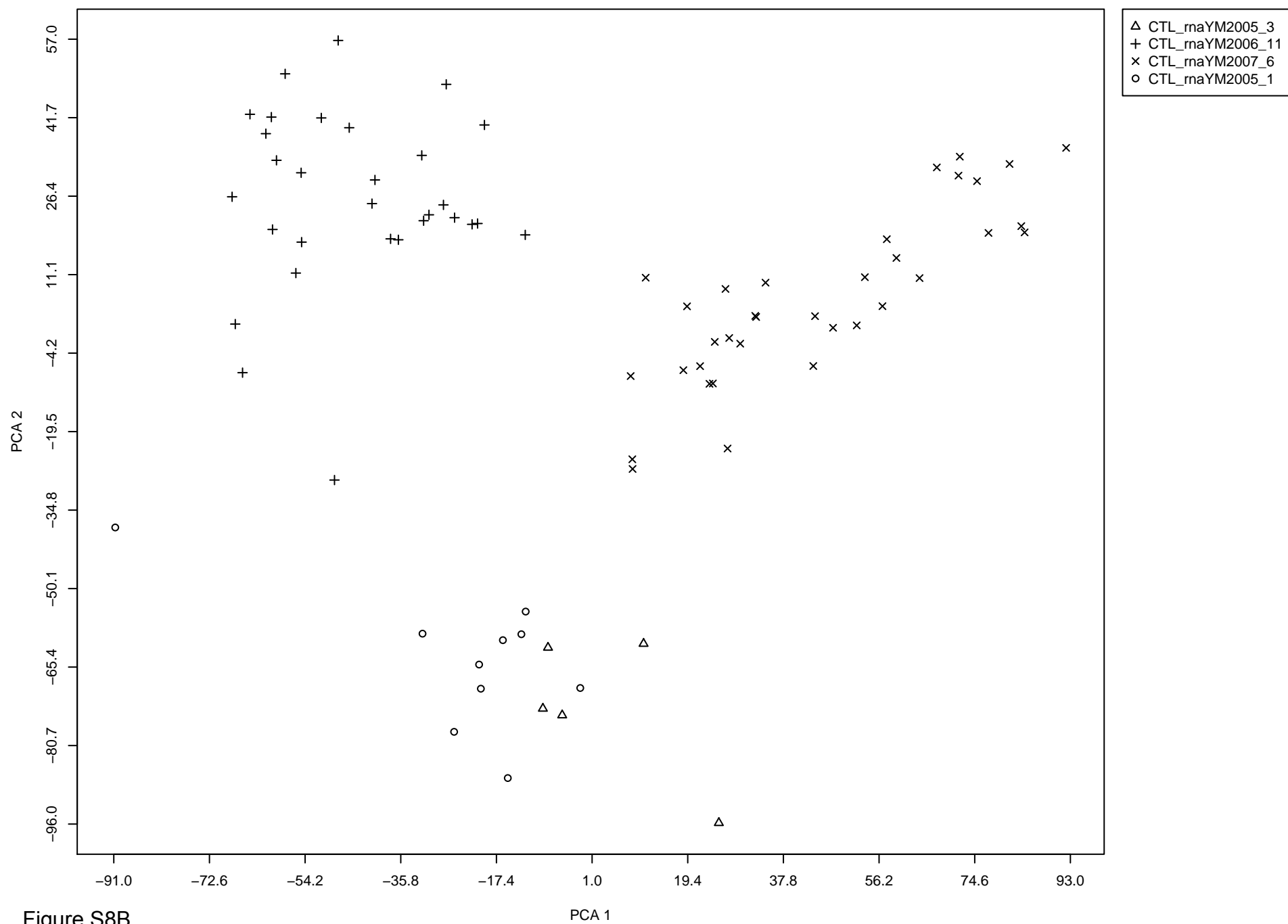

Figure S8B

PCA 2

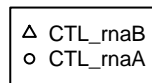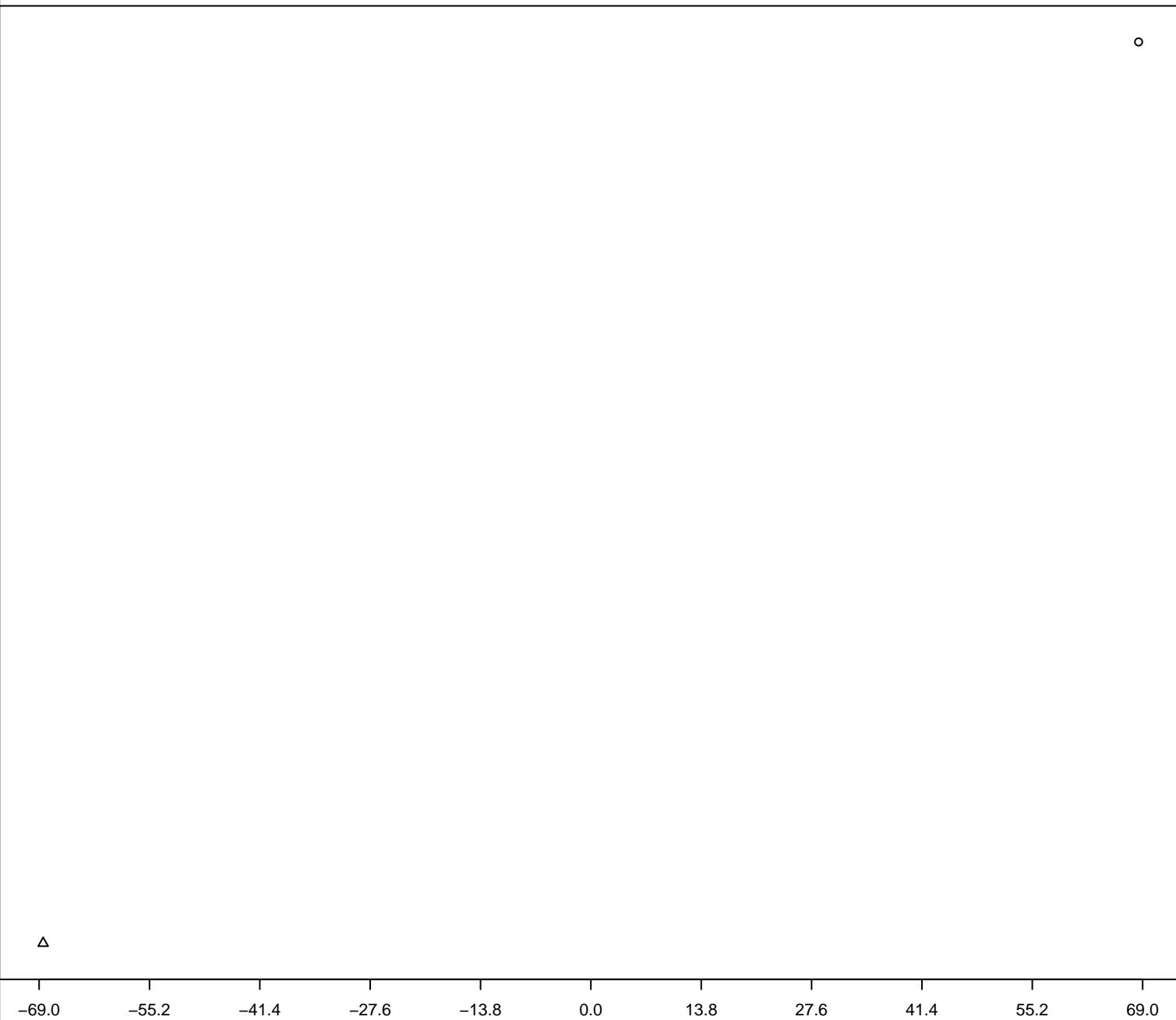

Figure S9A

PCA 1

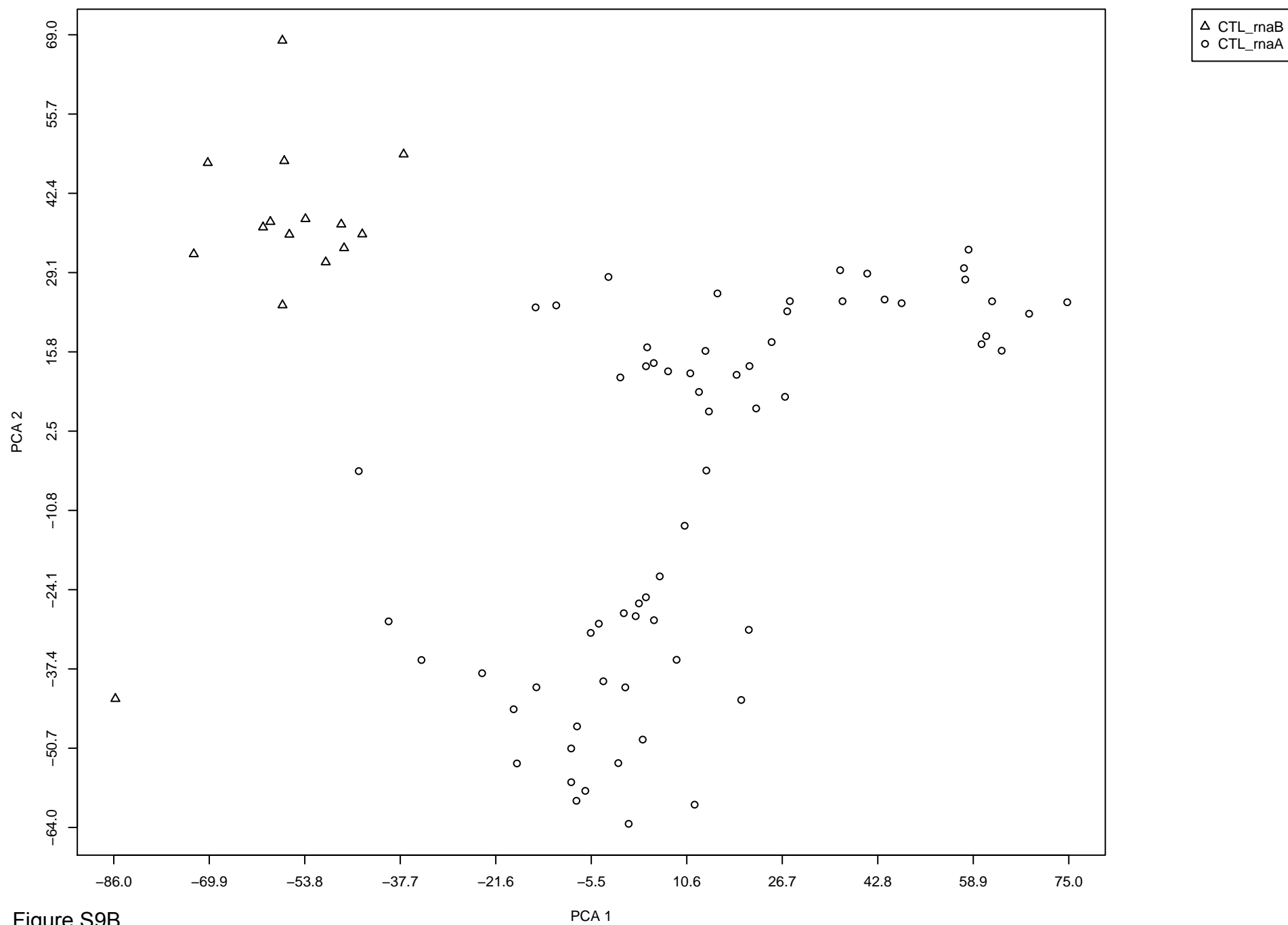

Figure S9B

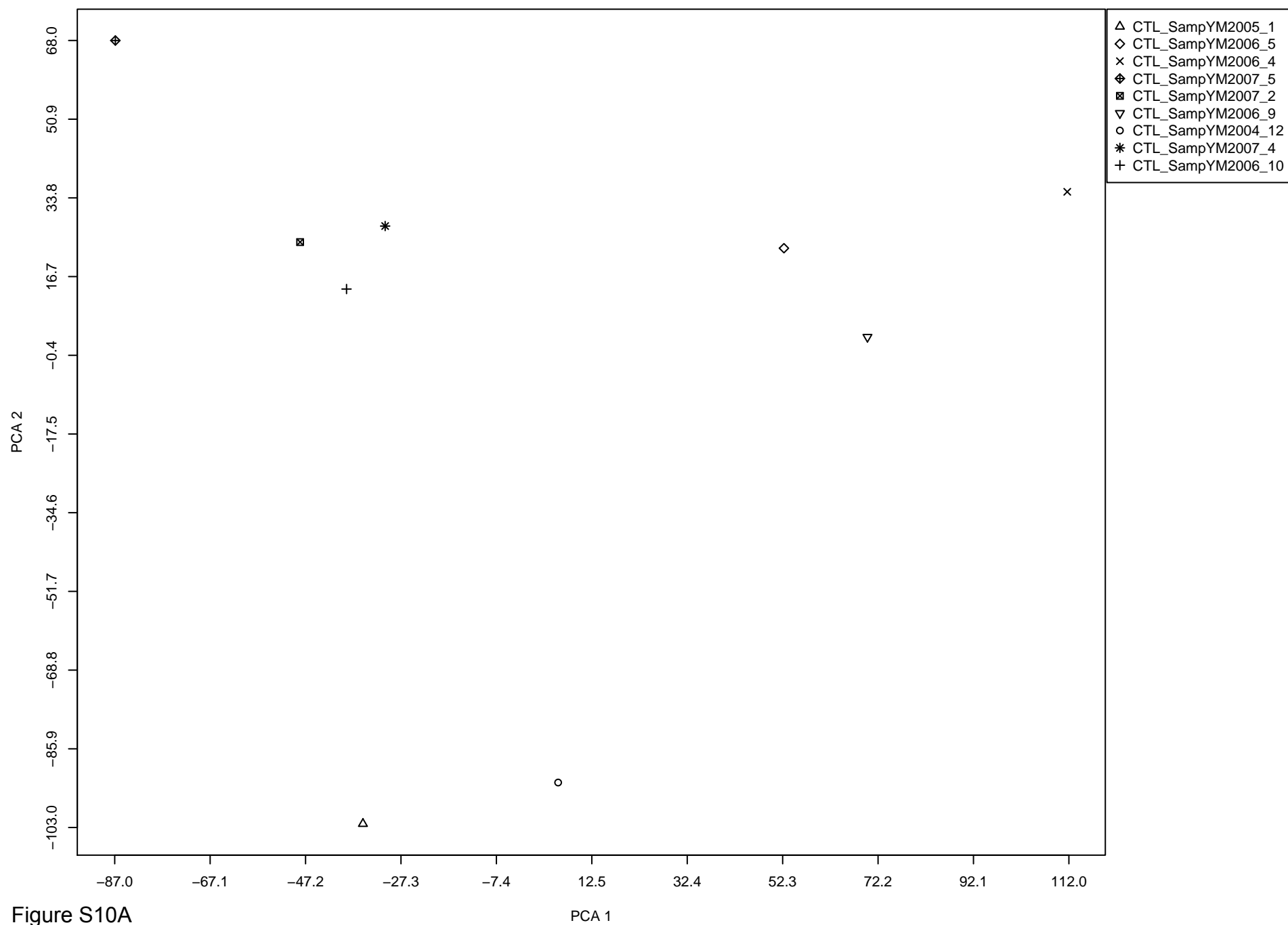

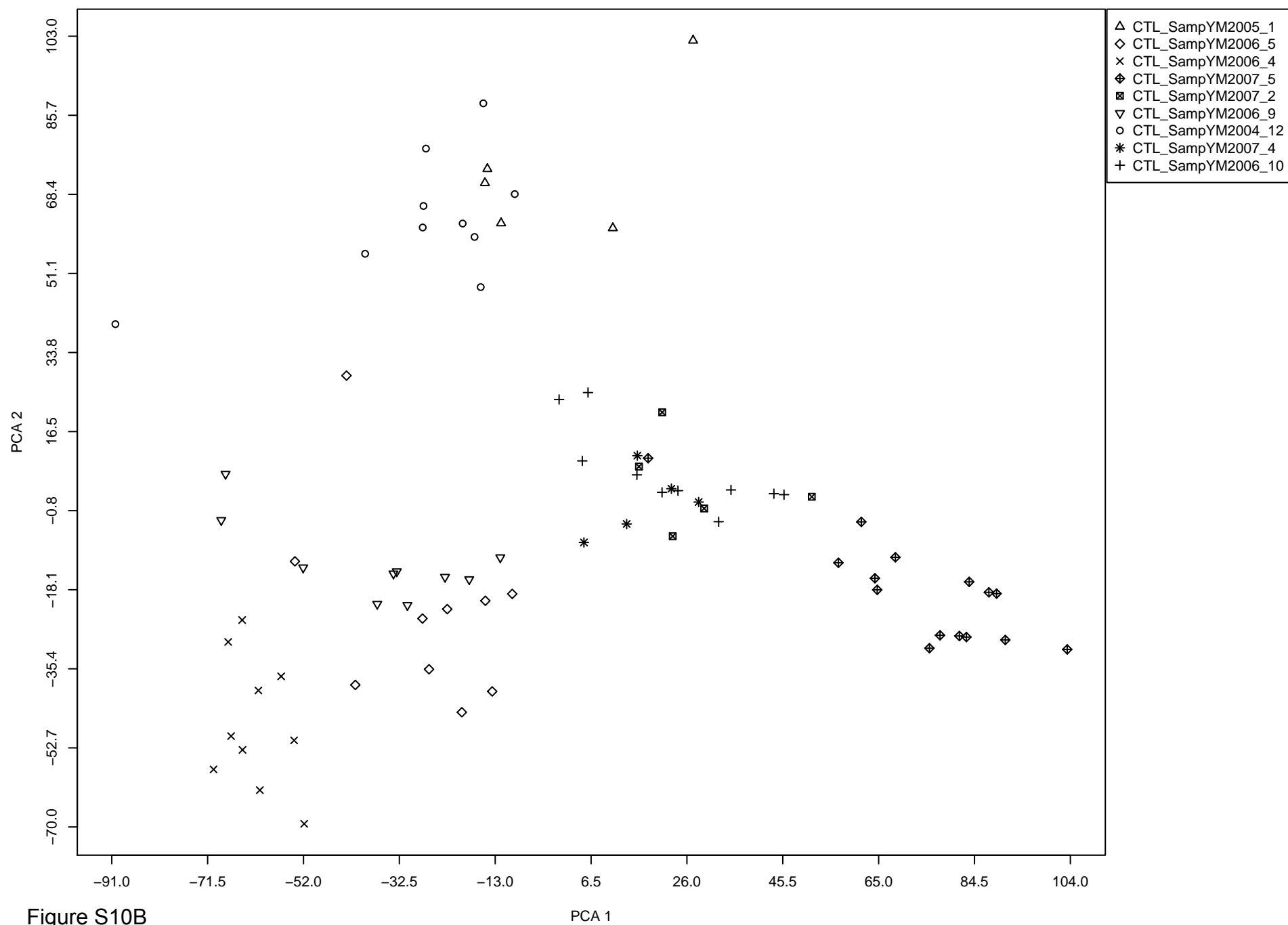

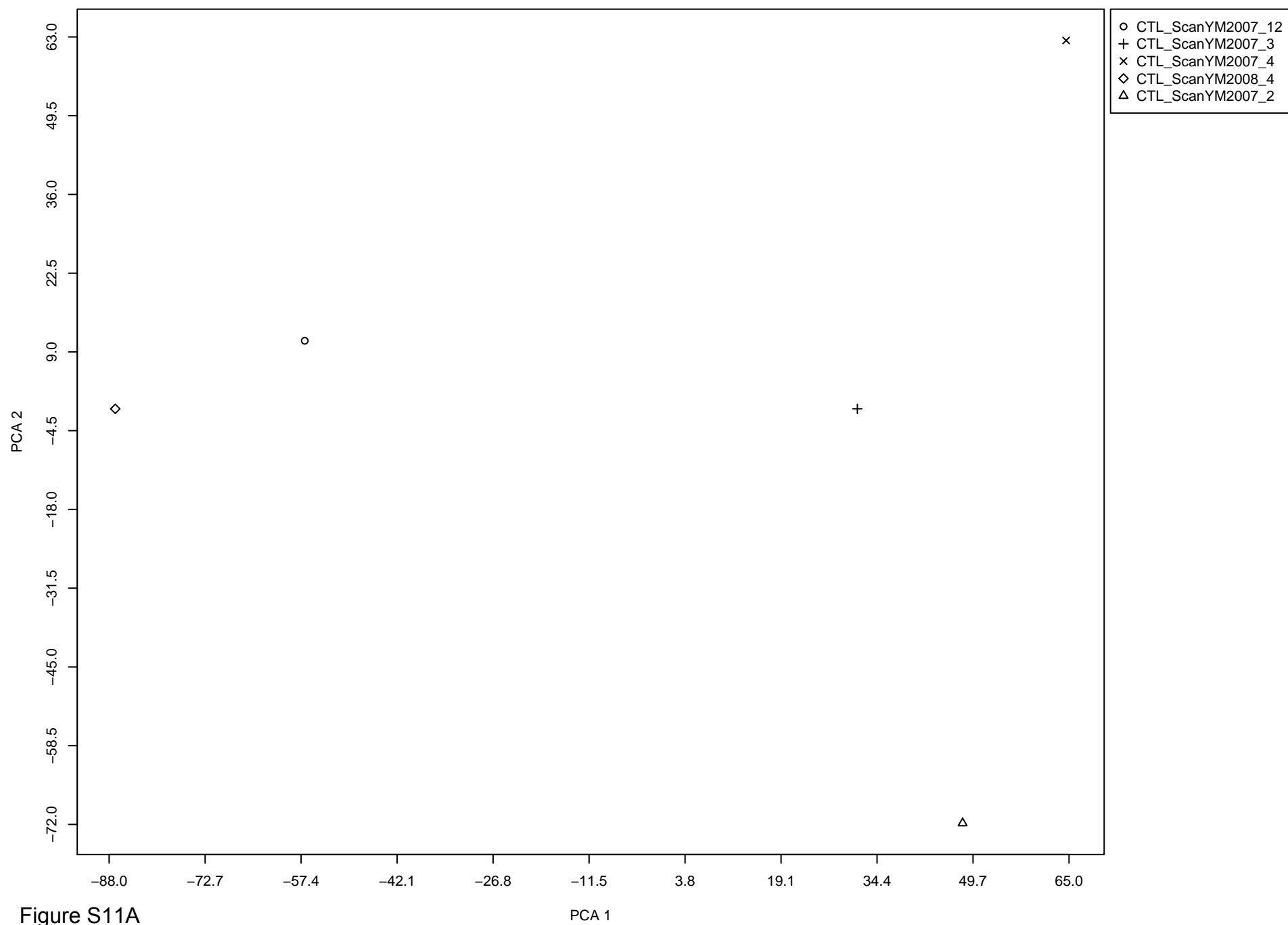

Figure S11A

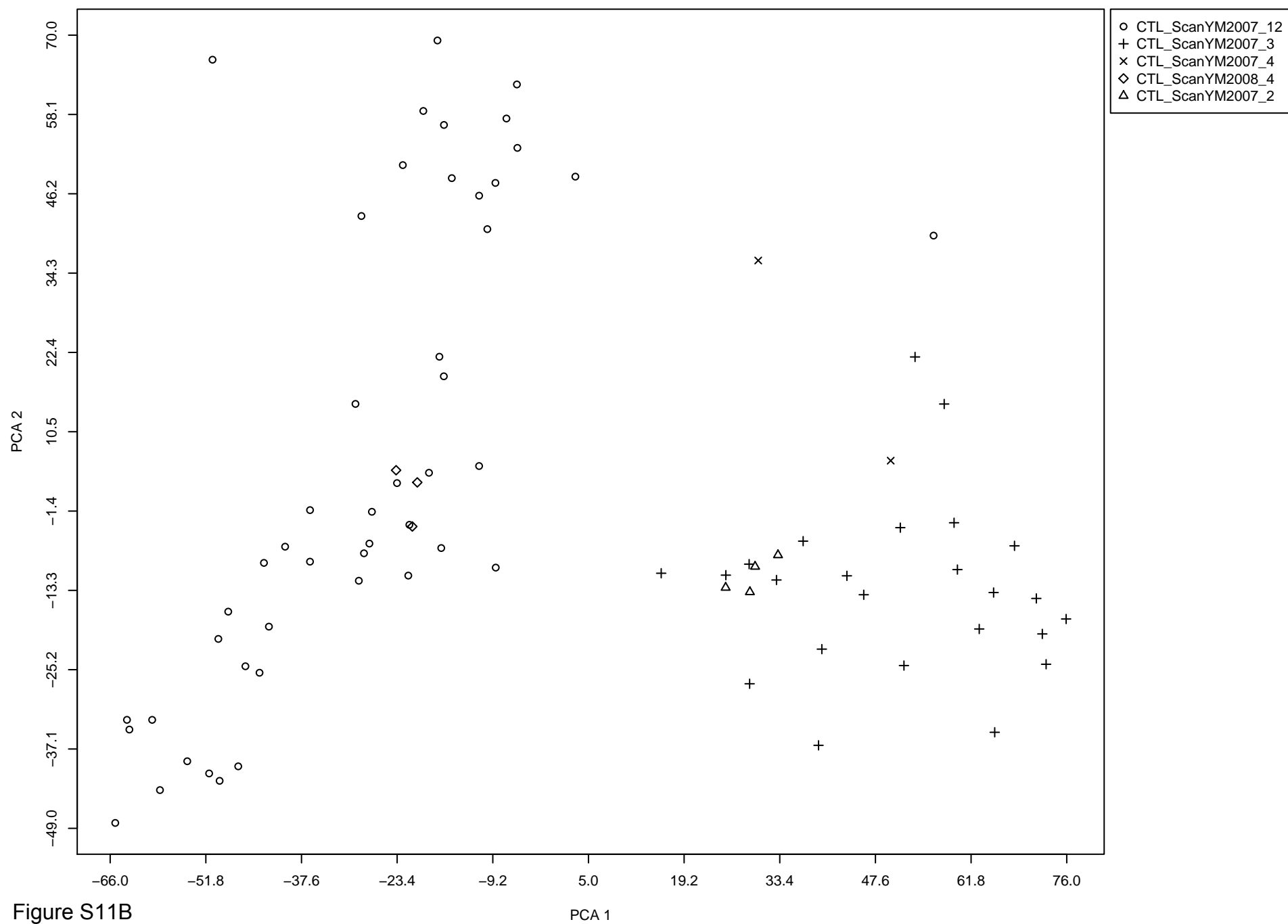

Figure S11B

Supplement: S3 File — The PCA plots of zebrafish samples based on all the DEGs identified as between-batch variation. Samples were grouped by Experiment (Figure S7A, S7B), RNA Date (Figure S8A, S8B), RNA Person (Figure S9A, S9B), Sampling Date (Figure S10A, S10B), and Scan Date (Figure S11A, S11B). Each figure was based on either the average gene intensity by individual batches (A) or the gene intensity of individual samples (B). DEGs were based on the simulated reference method. (PDF) [file pone.0114178.s009.pdf]

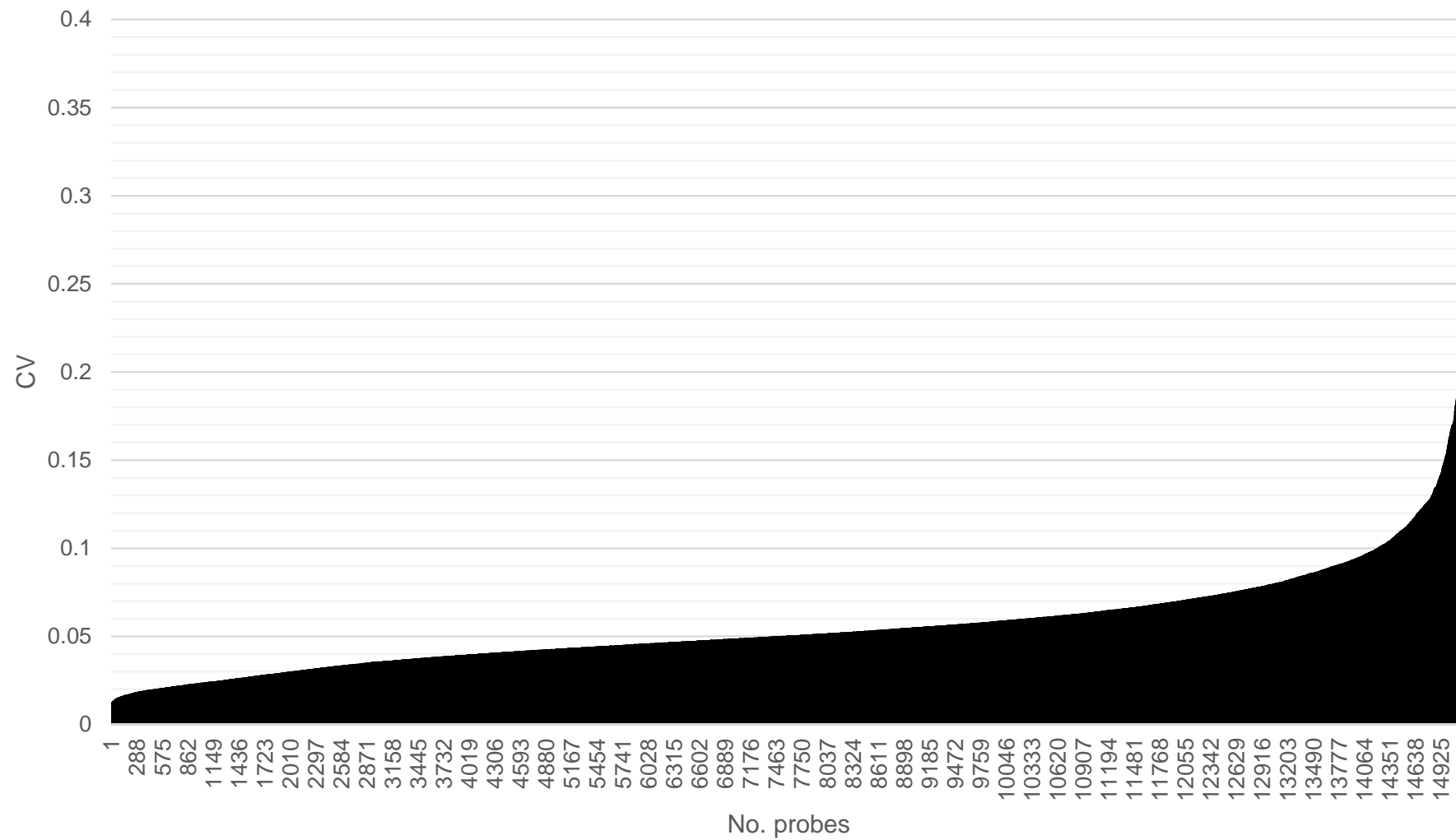

Figure S22A

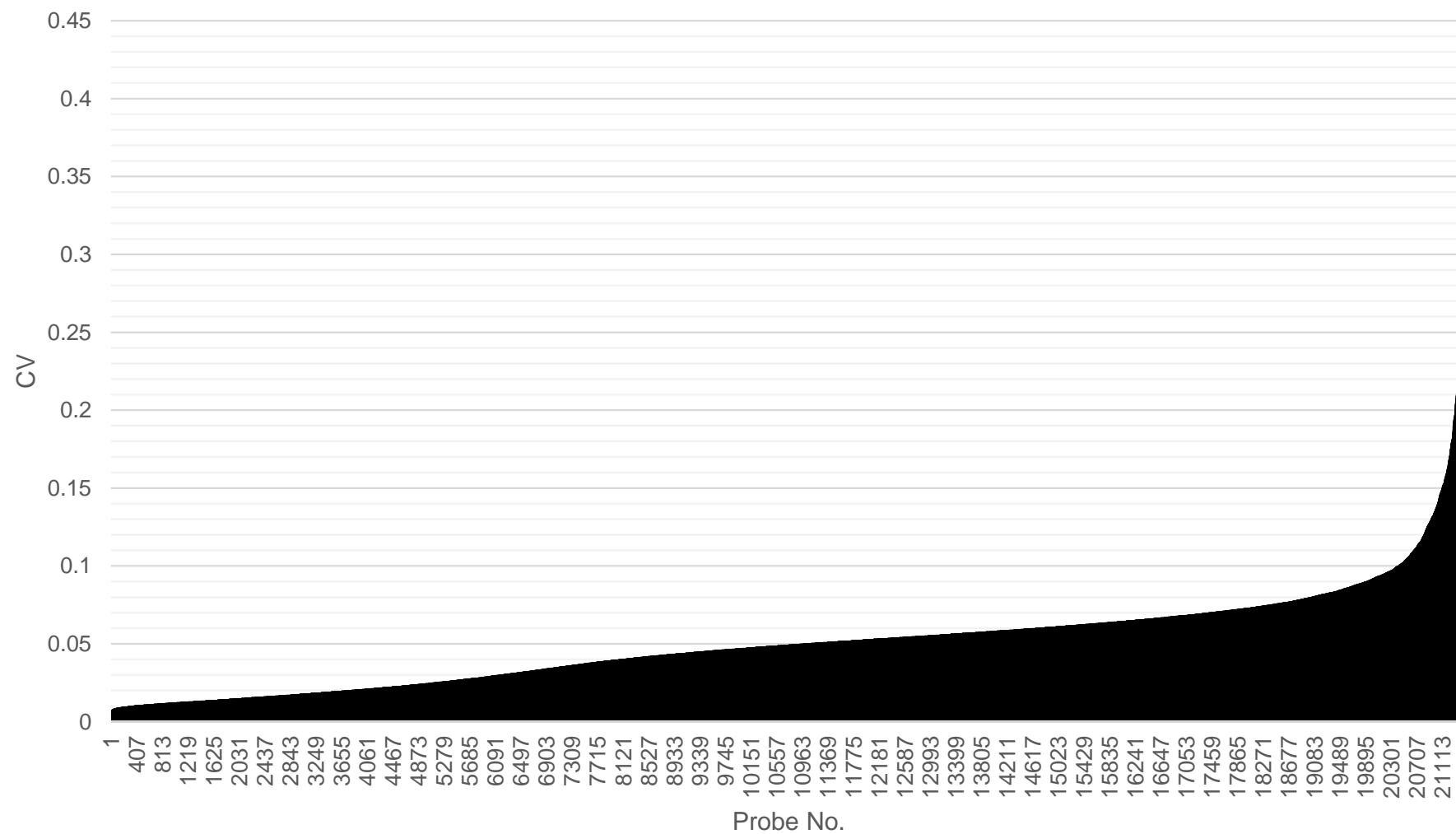

Figure S22B

Supplement: S6 File — The distribution of within-batch coefficients of variation (CV) of probe intensities. Fathead minnow (Figure S22A) CVs were based on 15208 probes, and zebrafish (Figure S22B) CVs were based on 21495 probes. (PDF) [file pone.0114178.s012.pdf]
