# Supplementary material for: Natural Variation in Fish Transcriptomes: Comparative Analysis of the Fathead Minnow (Pimephales promelas) and Zebrafish (Danio rerio)
Source: PLoS One. 2014 Dec 10;9(12):e114178. doi: 10.1371/journal.pone.0114178 (PMC4262388; doi:10.1371/journal.pone.0114178)
Supplement: S4 File — The dendrograms from resampling clustering of fathead minnow samples based on all the DEGs identified as between-batch variation. Samples were grouped by Experiment (Figure S12A, S12B), RNA Date (Figure S13A, S13B), RNA Person (Figure S14A, S14B), Sampling Date (Figure S15A, S15B), and Scan Date (Figure S16A, S16B). Each figure was based on either the average gene intensity by individual batches (A) or the gene intensity of individual samples (B). DEGs were based on the simulated reference method. (PDF) [file pone.0114178.s010.pdf]

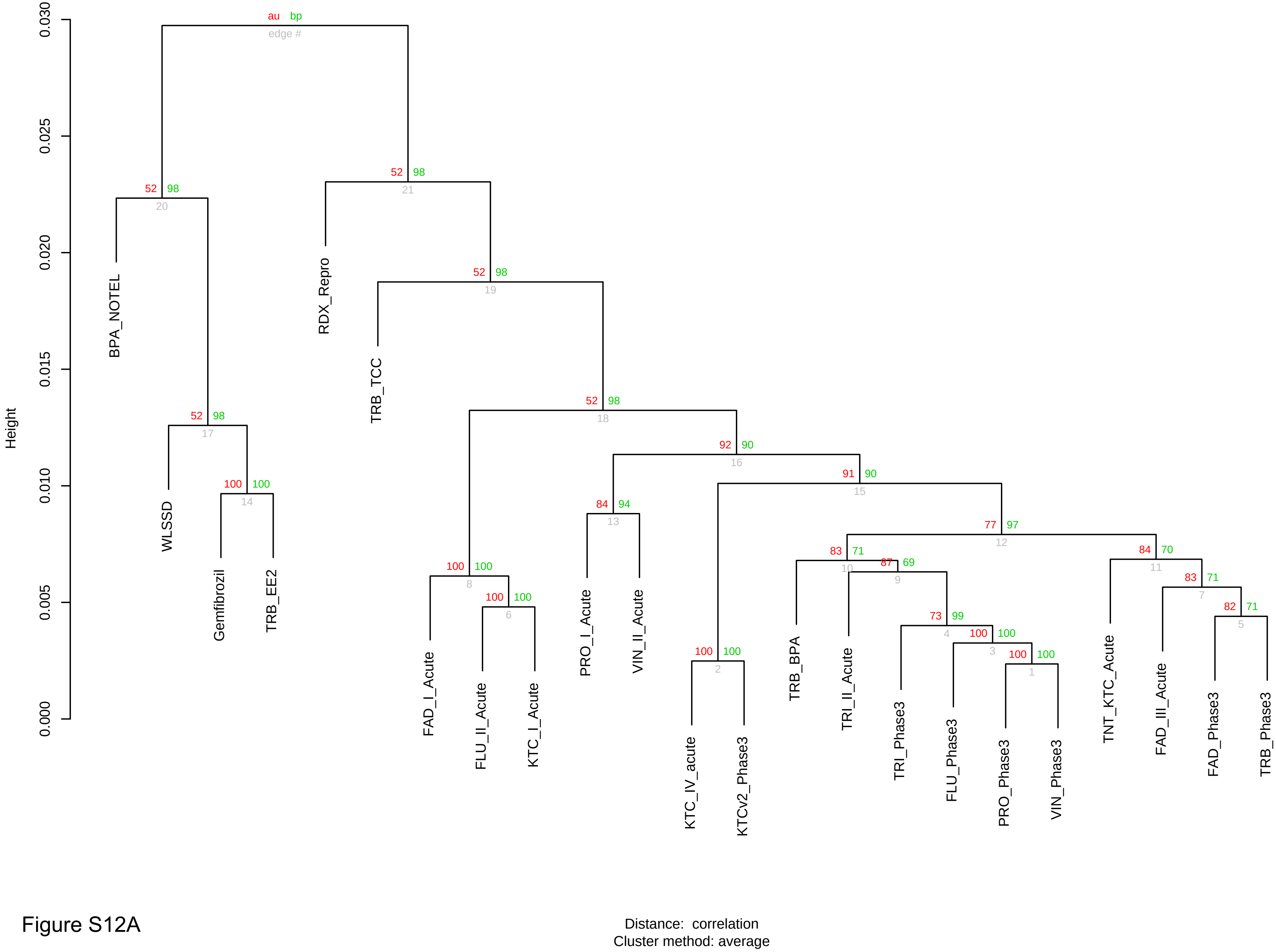

Height
0.030
0.025
0.020
0.015
0.010
0.005
0.000
au
bp
edge #
BPA_NOTEL
WLSSD
Gemfibrozil
TRB_EE2
RDX_Repro
TRB_TCC
FAD_I_Acute
FLU_II_Acute
KTC_I_Acute
PRO_I_Acute
VIN_II_Acute
KTC_IV_acute
KTCv2_Phase3
TRB_BPA
TRI_II_Acute
TRI_Phase3
FLU_Phase3
PRO_Phase3
VIN_Phase3
TNT_KTC_Acute
FAD_III_Acute
FAD_Phase3
TRB_Phase3
Distance: correlation
Cluster method: average

Figure S12A

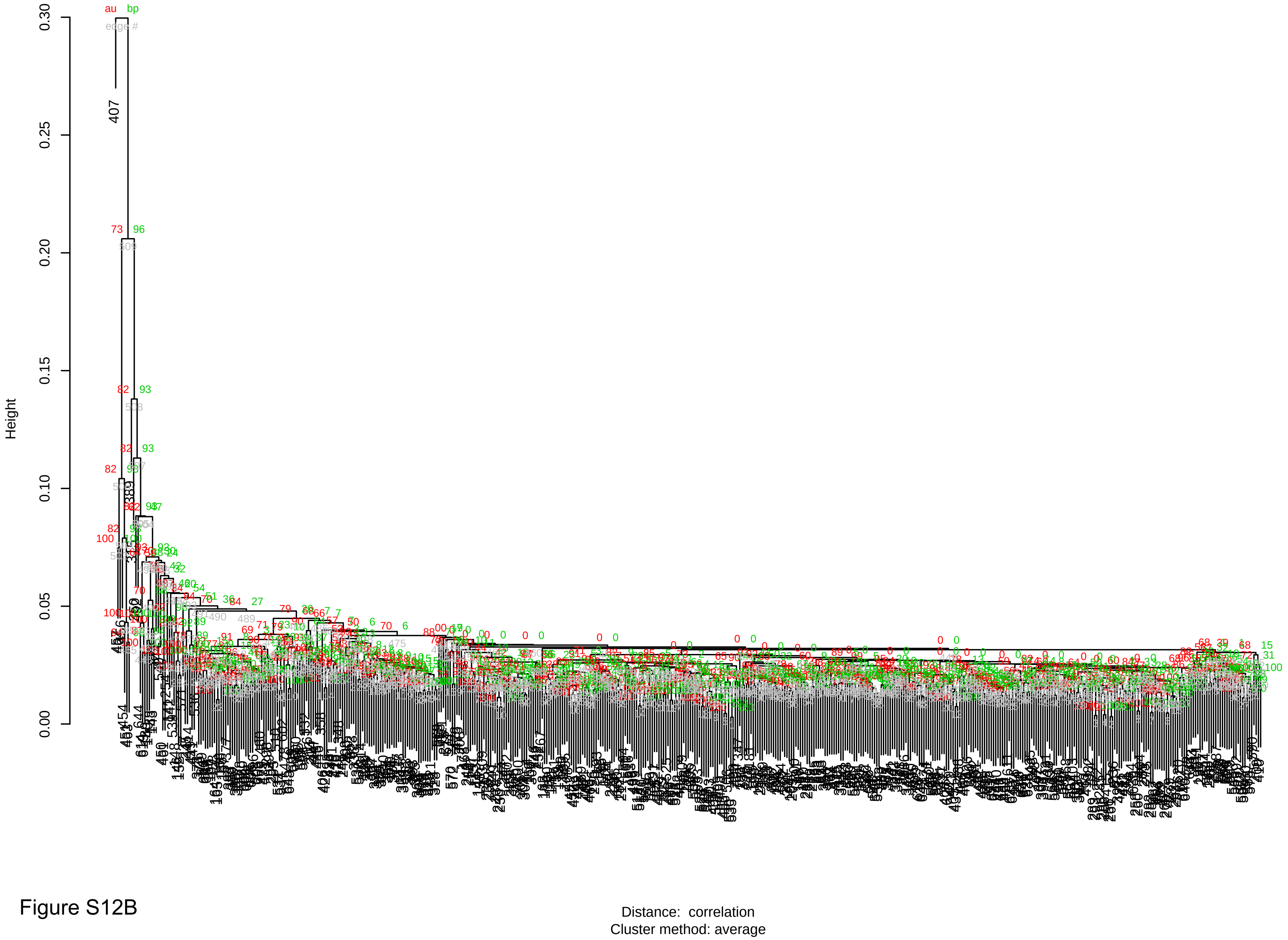

au
bp
edge #
Height
Distance: correlation
Cluster method: average

Figure S12B

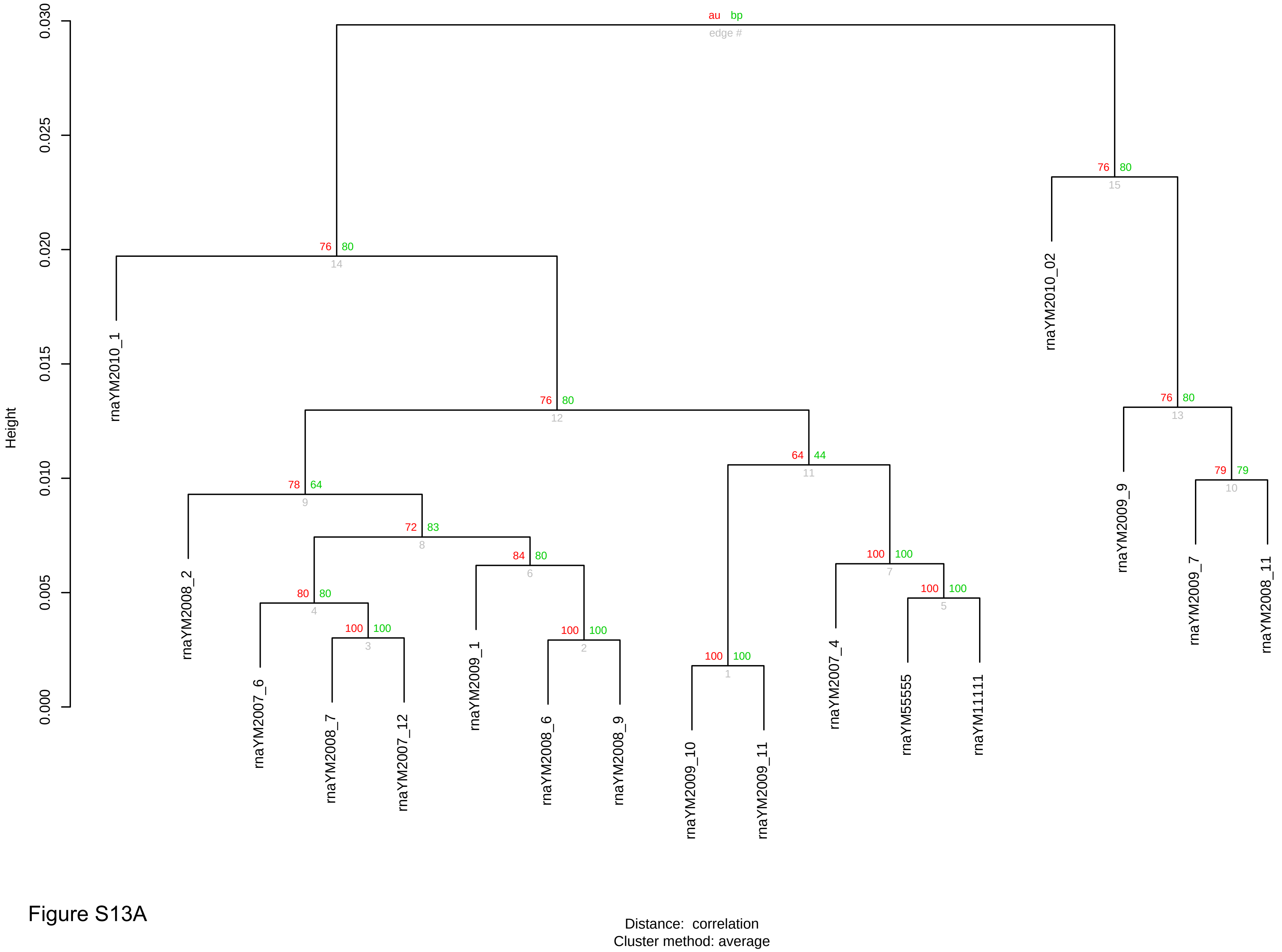

au bp
edge #
Height
0.000
0.005
0.010
0.015
0.020
0.025
0.030
rnaYM2010_1
rnaYM2008_2
rnaYM2007_6
rnaYM2008_7
rnaYM2007_12
rnaYM2009_1
rnaYM2008_6
rnaYM2008_9
rnaYM2009_10
rnaYM2009_11
rnaYM2007_4
rnaYM55555
rnaYM11111
rnaYM2010_02
rnaYM2009_9
rnaYM2009_7
rnaYM2008_11
Distance: correlation
Cluster method: average

Figure S13A

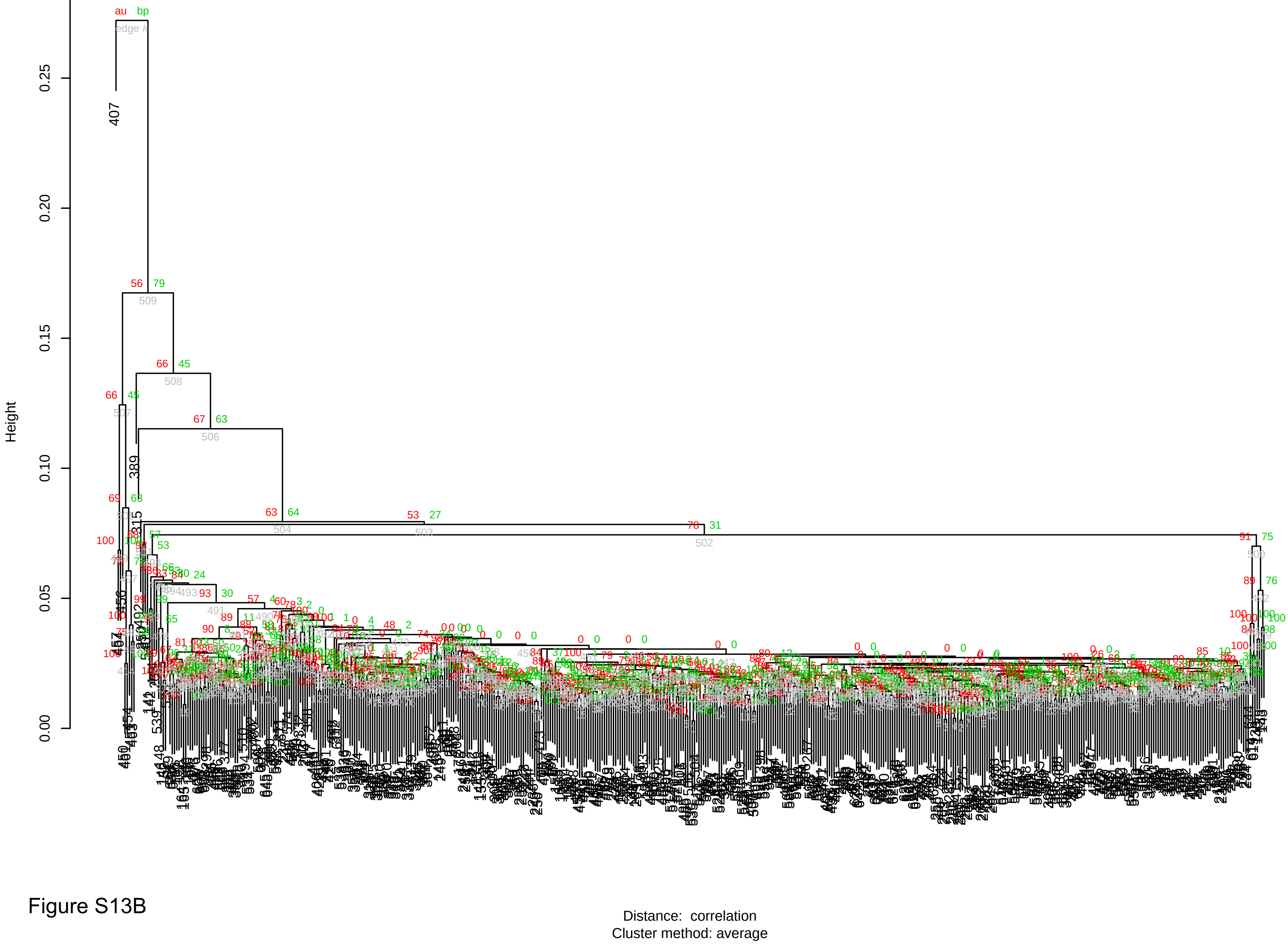
Height
0.00
0.05
0.10
0.15
0.20
0.25
au
bp
edge #
Distance: correlation
Cluster method: average

Figure S13B

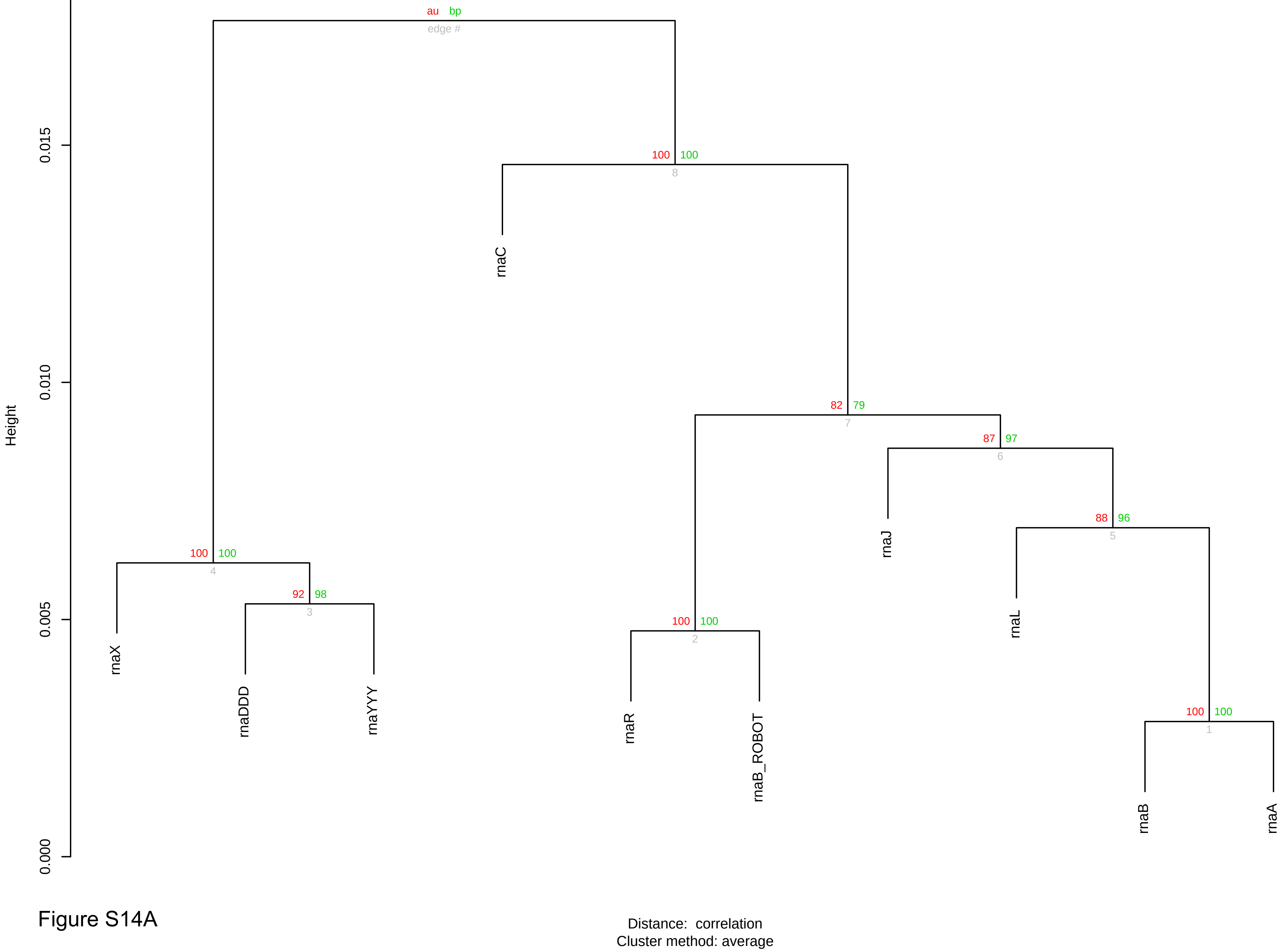

au
bp
edge #
Height
0.015
0.010
0.005
0.000
100
100
8
rnaC
82
79
7
87
97
6
88
96
5
100
100
4
92
98
3
100
100
2
100
100
1
rnaX
rnaDDD
rnaYYY
rnaR
rnaB_ROBOT
rnaJ
rnaL
rnaB
rnaA
Distance: correlation
Cluster method: average

Figure S14A

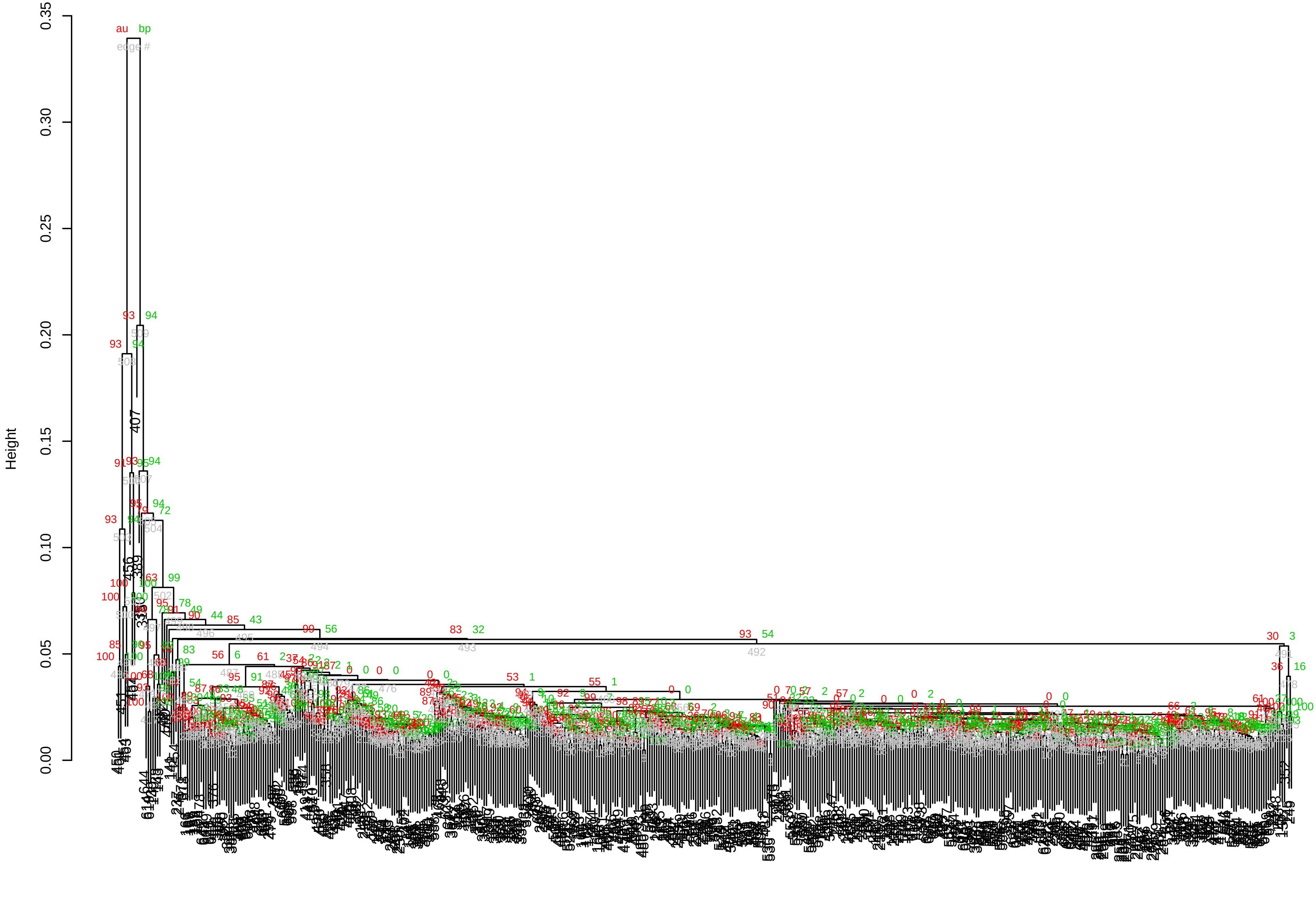

Figure S14B

Distance: correlation
Cluster method: average

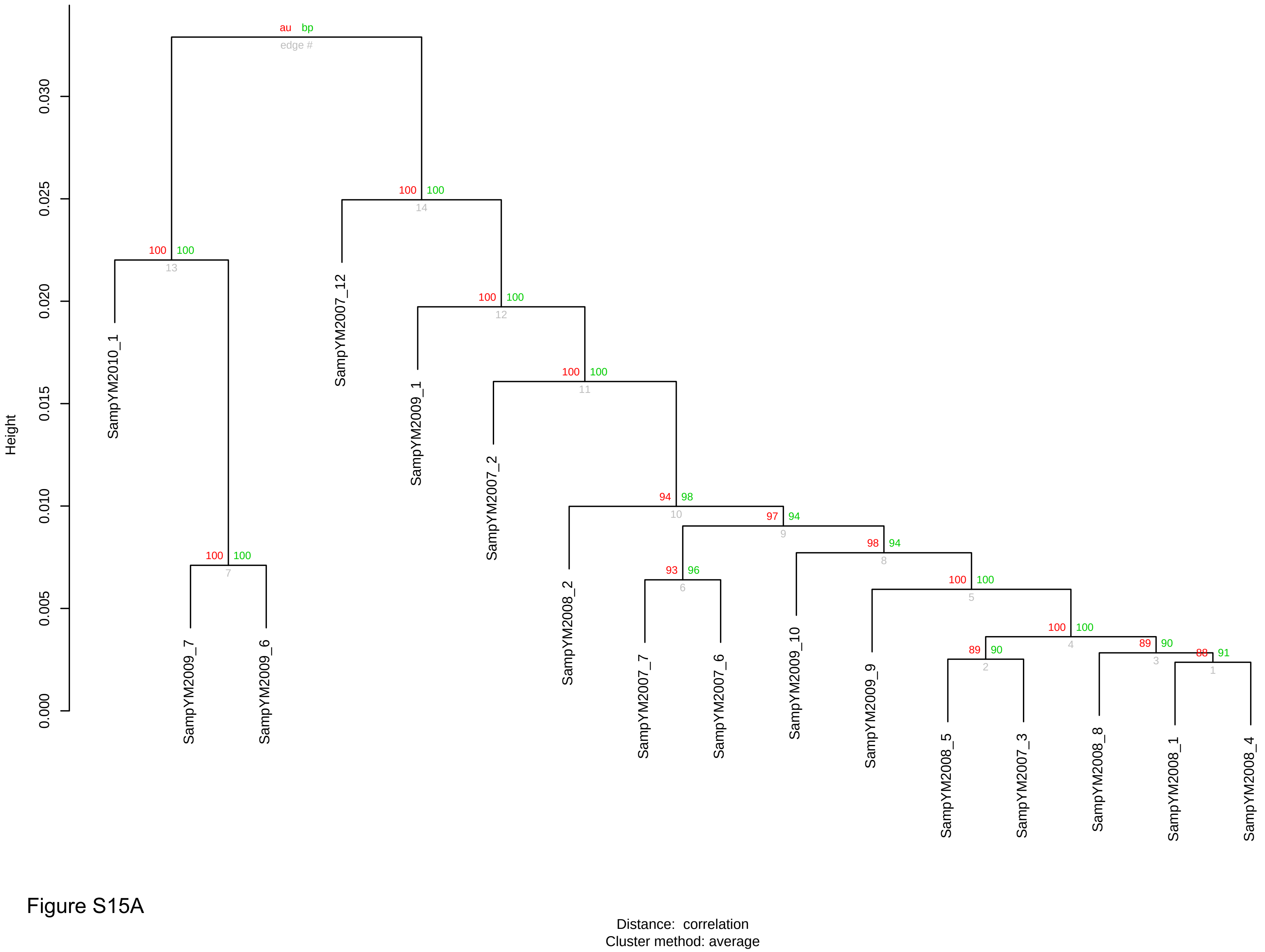
au bp
edge #
Height
0.000
0.005
0.010
0.015
0.020
0.025
0.030
SampYM2010_1
SampYM2009_7
SampYM2009_6
SampYM2007_12
SampYM2009_1
SampYM2007_2
SampYM2008_2
SampYM2007_7
SampYM2007_6
SampYM2009_10
SampYM2009_9
SampYM2008_5
SampYM2007_3
SampYM2008_8
SampYM2008_1
SampYM2008_4
Distance: correlation
Cluster method: average

Figure S15A

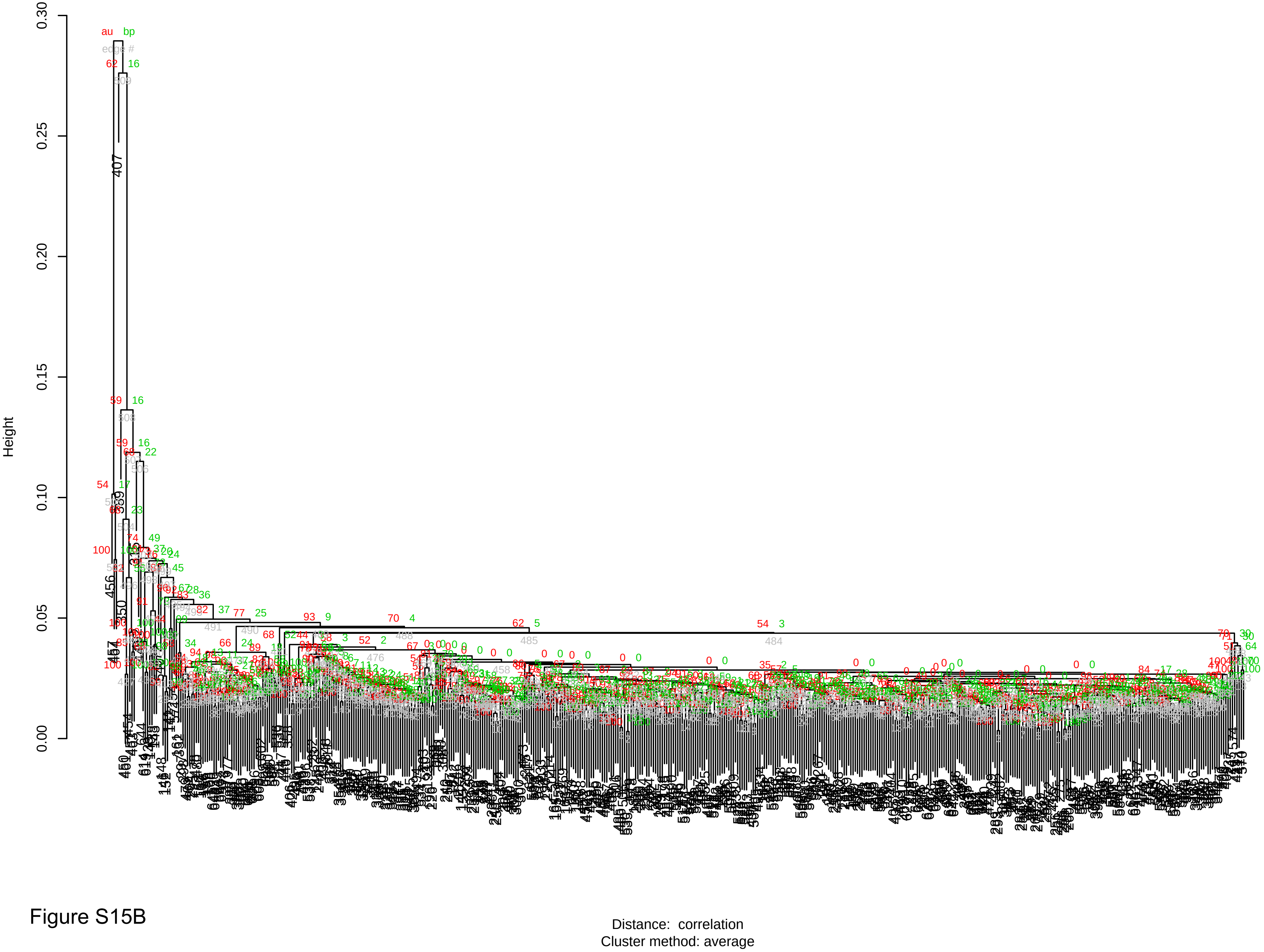
au
bp
edge #
Height
0.30
0.25
0.20
0.15
0.10
0.05
0.00
Distance: correlation
Cluster method: average

Figure S15B

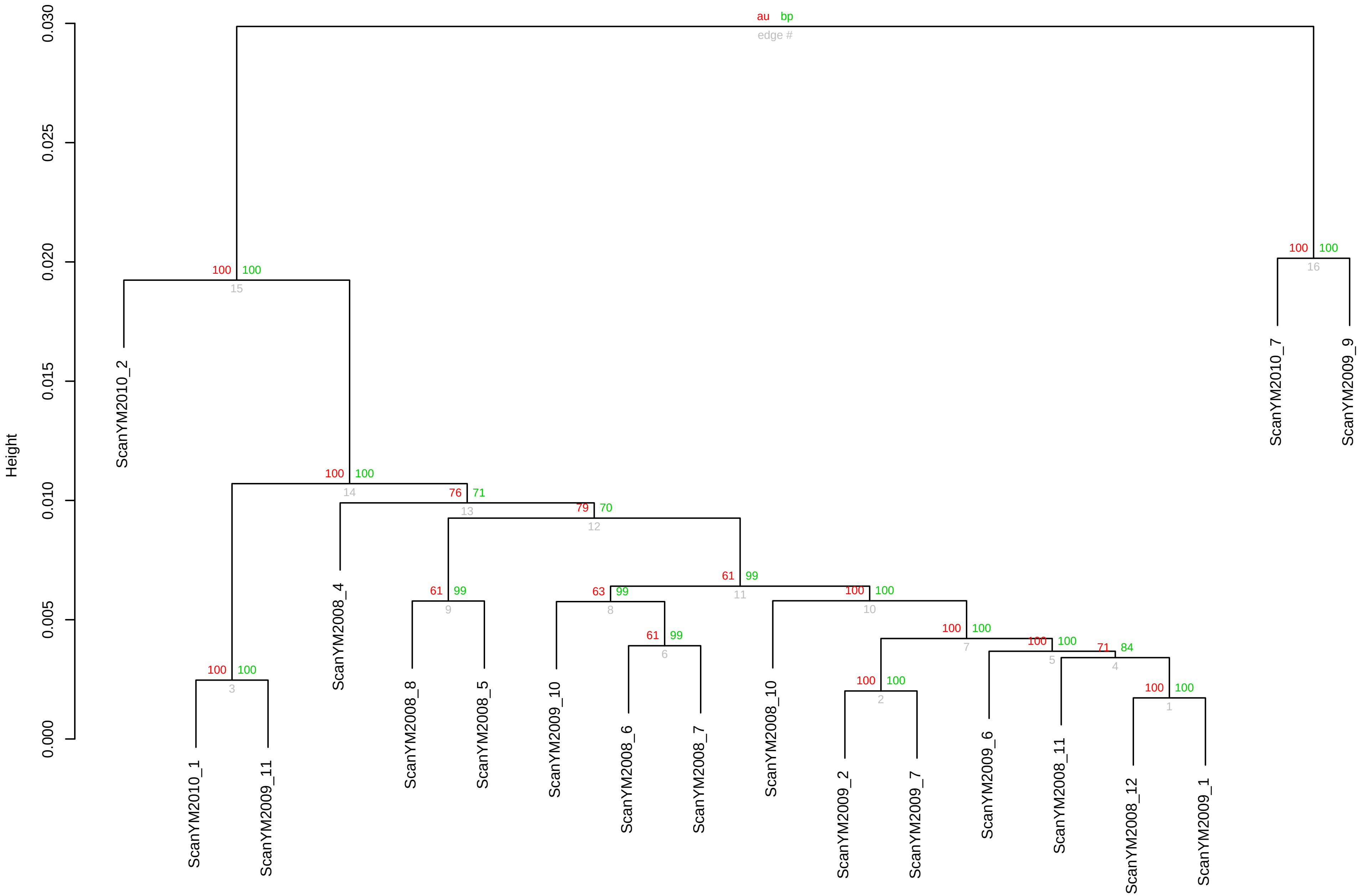

Figure S16A

Distance: correlation
Cluster method: average

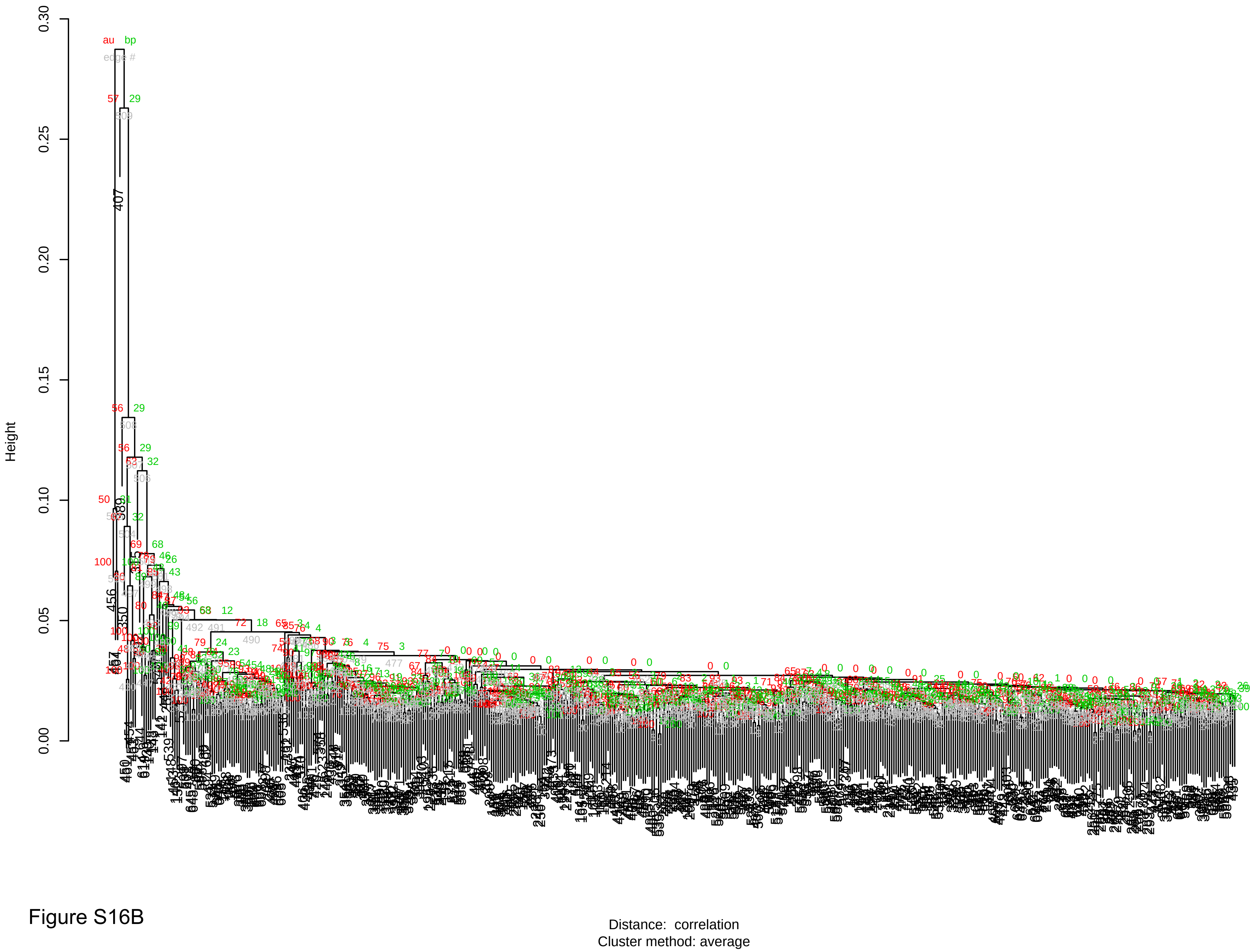
au
bp
edge #
Height
Distance: correlation
Cluster method: average

Figure S16B
