# Supplementary material for: Natural Variation in Fish Transcriptomes: Comparative Analysis of the Fathead Minnow (Pimephales promelas) and Zebrafish (Danio rerio)
Source: PLoS One. 2014 Dec 10;9(12):e114178. doi: 10.1371/journal.pone.0114178 (PMC4262388; doi:10.1371/journal.pone.0114178)
Supplement: S5 File — The dendrogram from resampling clustering of zebrafish samples based on all the DEGs identified as between-batch variation. Samples were grouped by Experiment (Figure S17A, S17B), RNA Date (Figure S18A, S18B), RNA Person (Figure S19A, S19B), Sampling Date (Figure S20A, S20B), and Scan Date (Figure S21A, S21B). Each figure was based on either the average gene intensity by individual batches (A) or the gene intensity of individual samples (B). DEGs were based on the simulated reference method. (PDF) [file pone.0114178.s011.pdf]

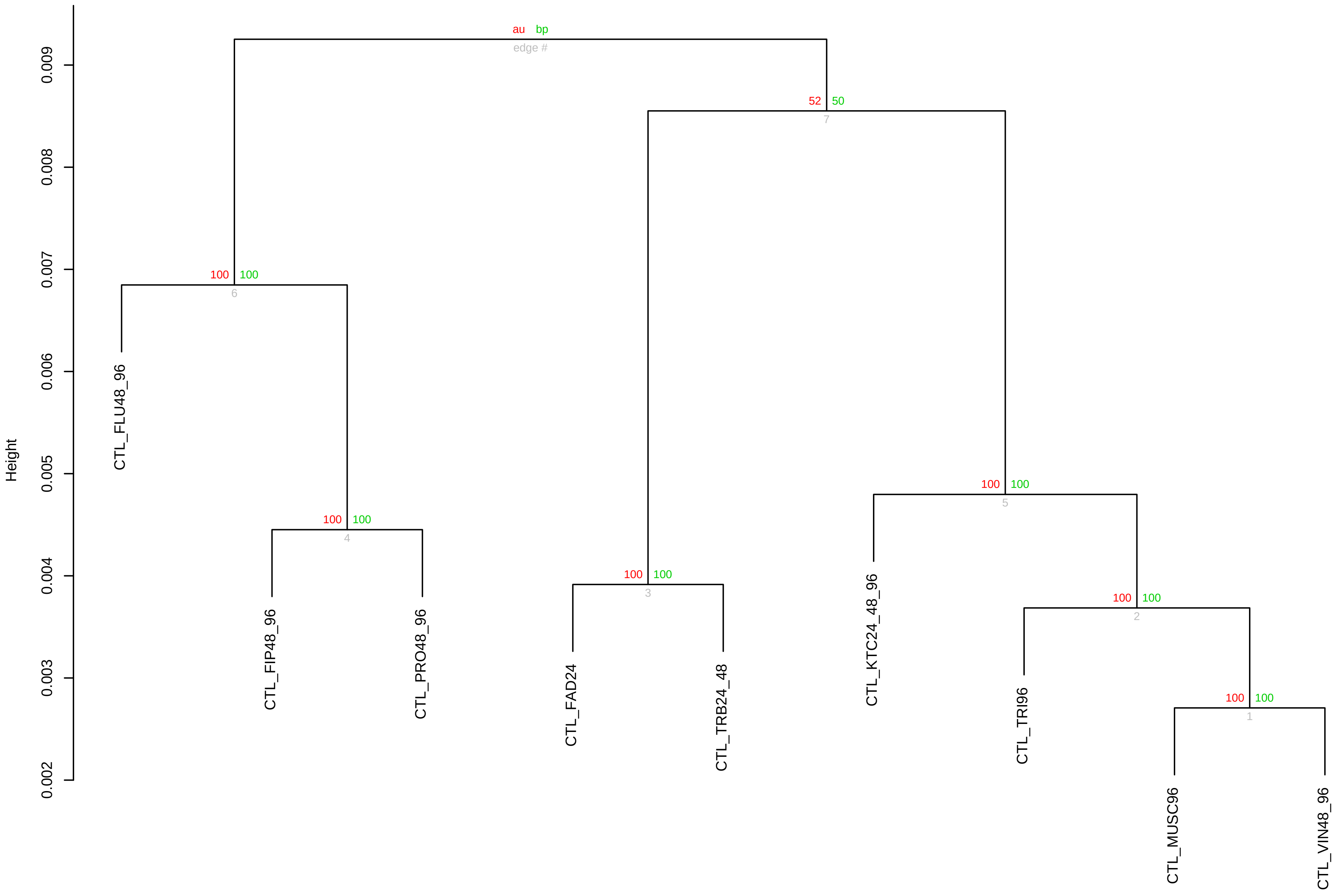

Figure S17A

Distance: correlation
Cluster method: average

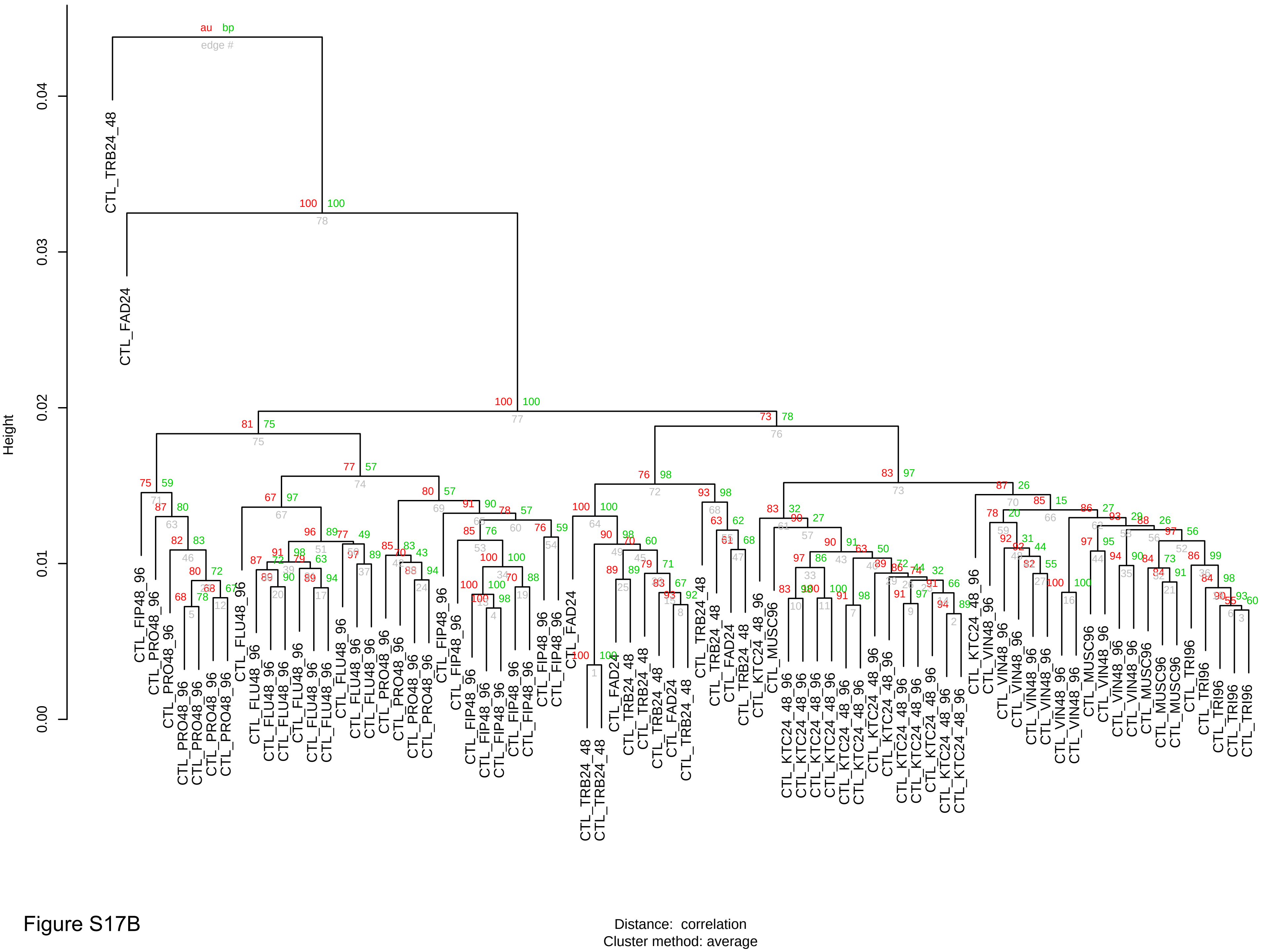
au bp
edge #
Height
0.00
0.01
0.02
0.03
0.04
CTL_TRB24_48
CTL_FAD24
Distance: correlation
Cluster method: average

Figure S17B

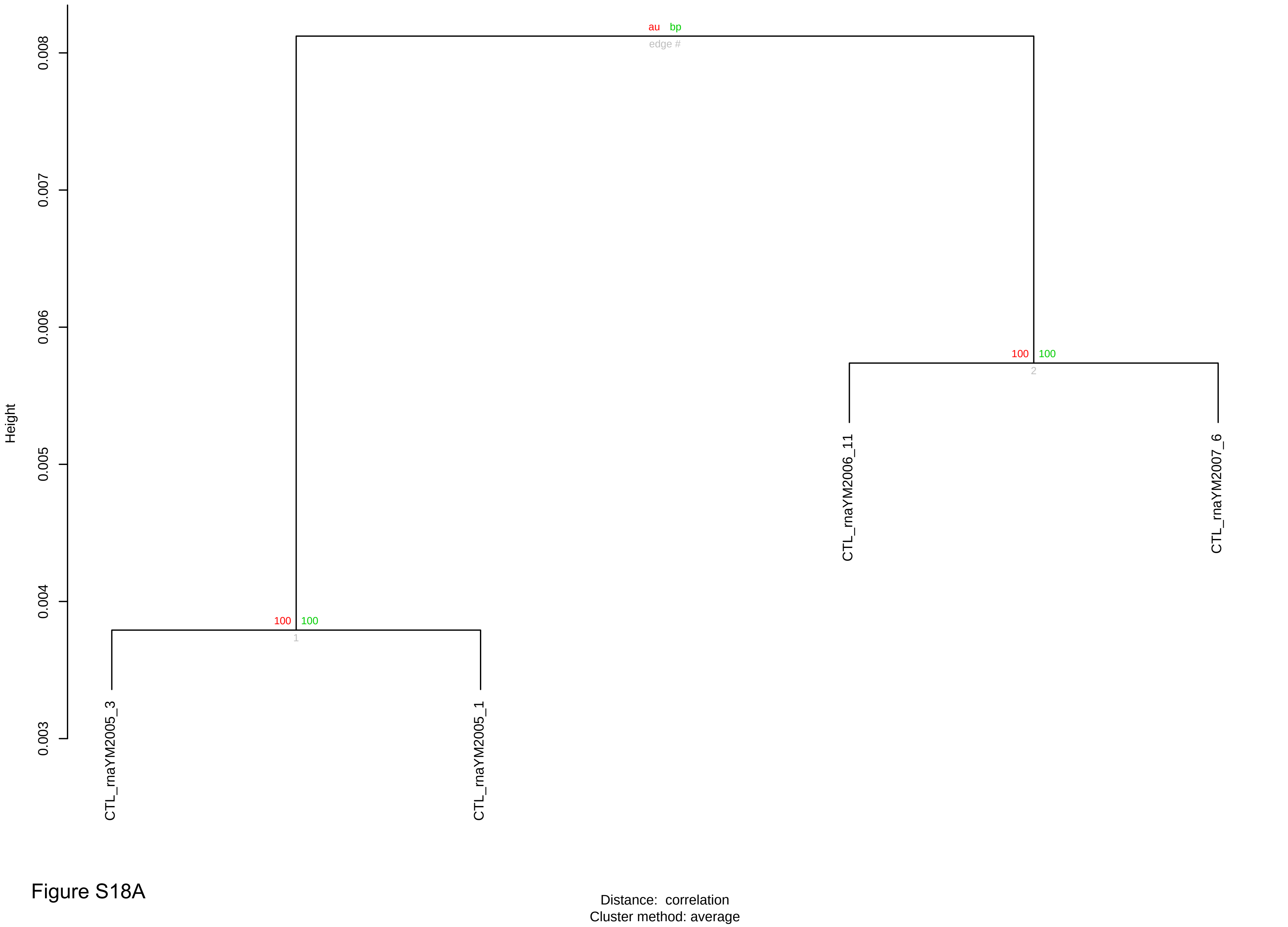
au bp
edge #
100 100
1
100 100
2
Height
0.003
0.004
0.005
0.006
0.007
0.008
CTL_rnaYM2005_3
CTL_rnaYM2005_1
CTL_rnaYM2006_11
CTL_rnaYM2007_6
Distance: correlation
Cluster method: average

Figure S18A

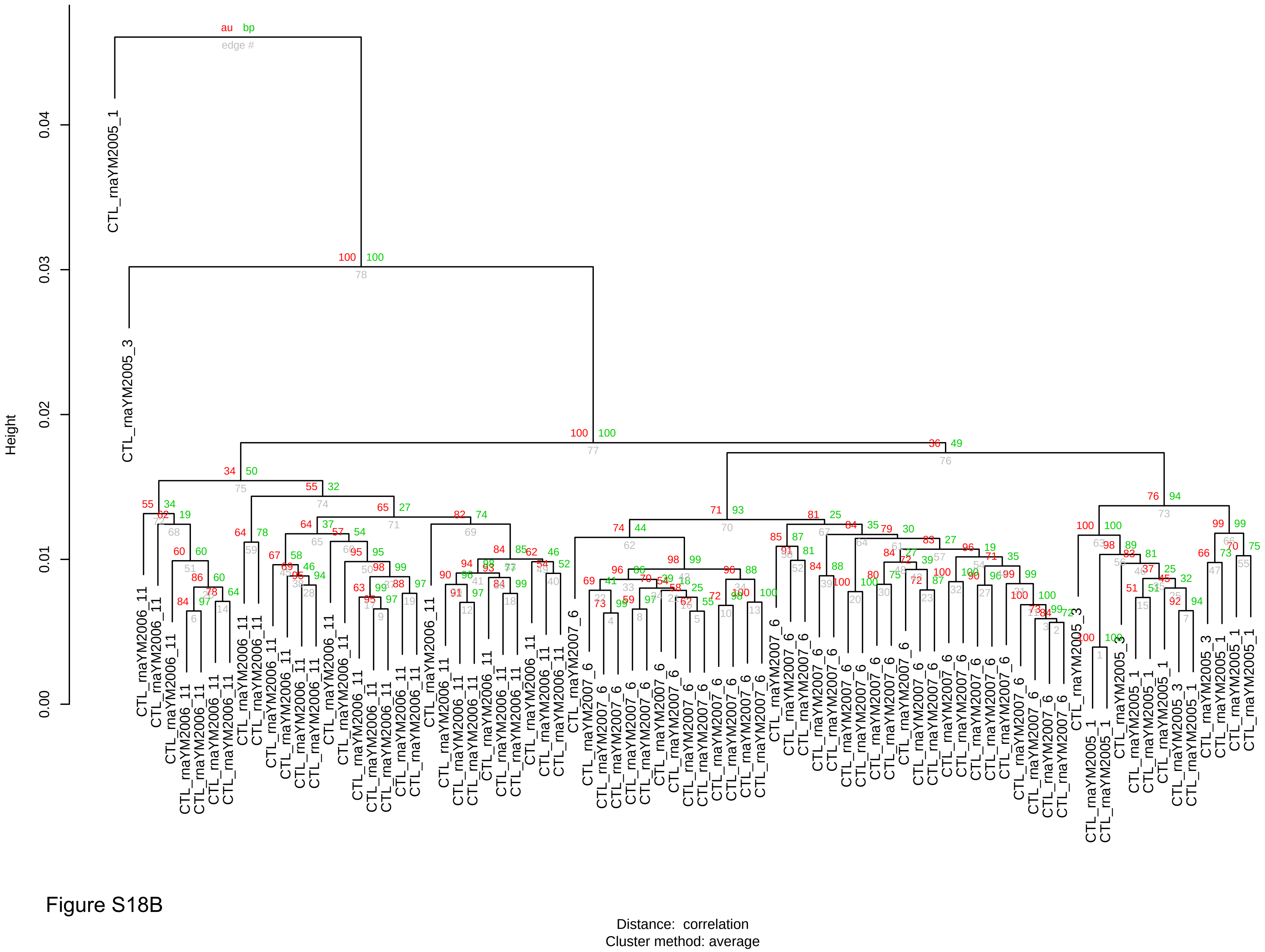
au bp
edge #
Height
0.00
0.01
0.02
0.03
0.04
Distance: correlation
Cluster method: average

Figure S18B

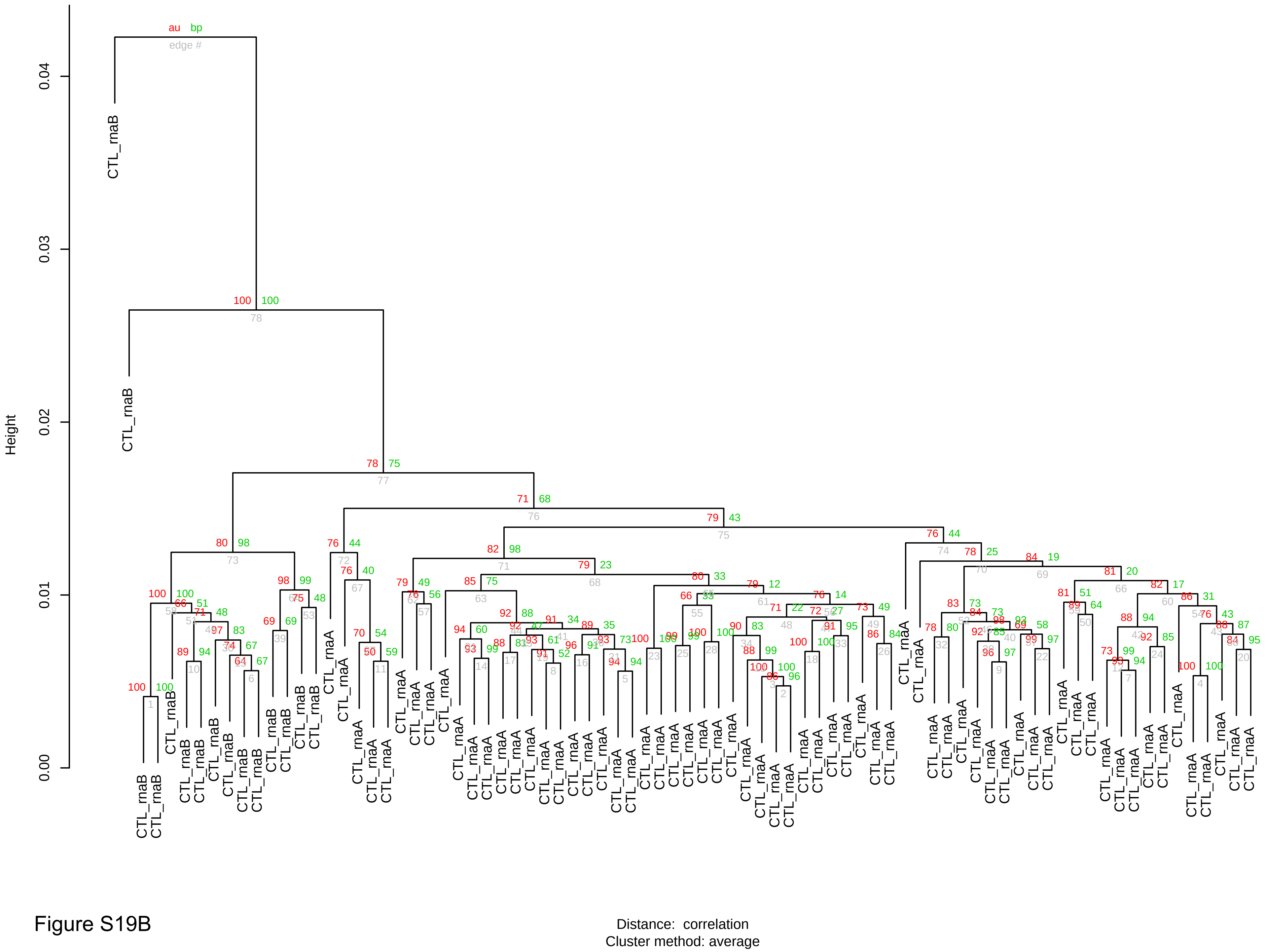
au bp
edge #
Height
CTL_rnaB
CTL_rnaA
Distance: correlation
Cluster method: average

Figure S19B

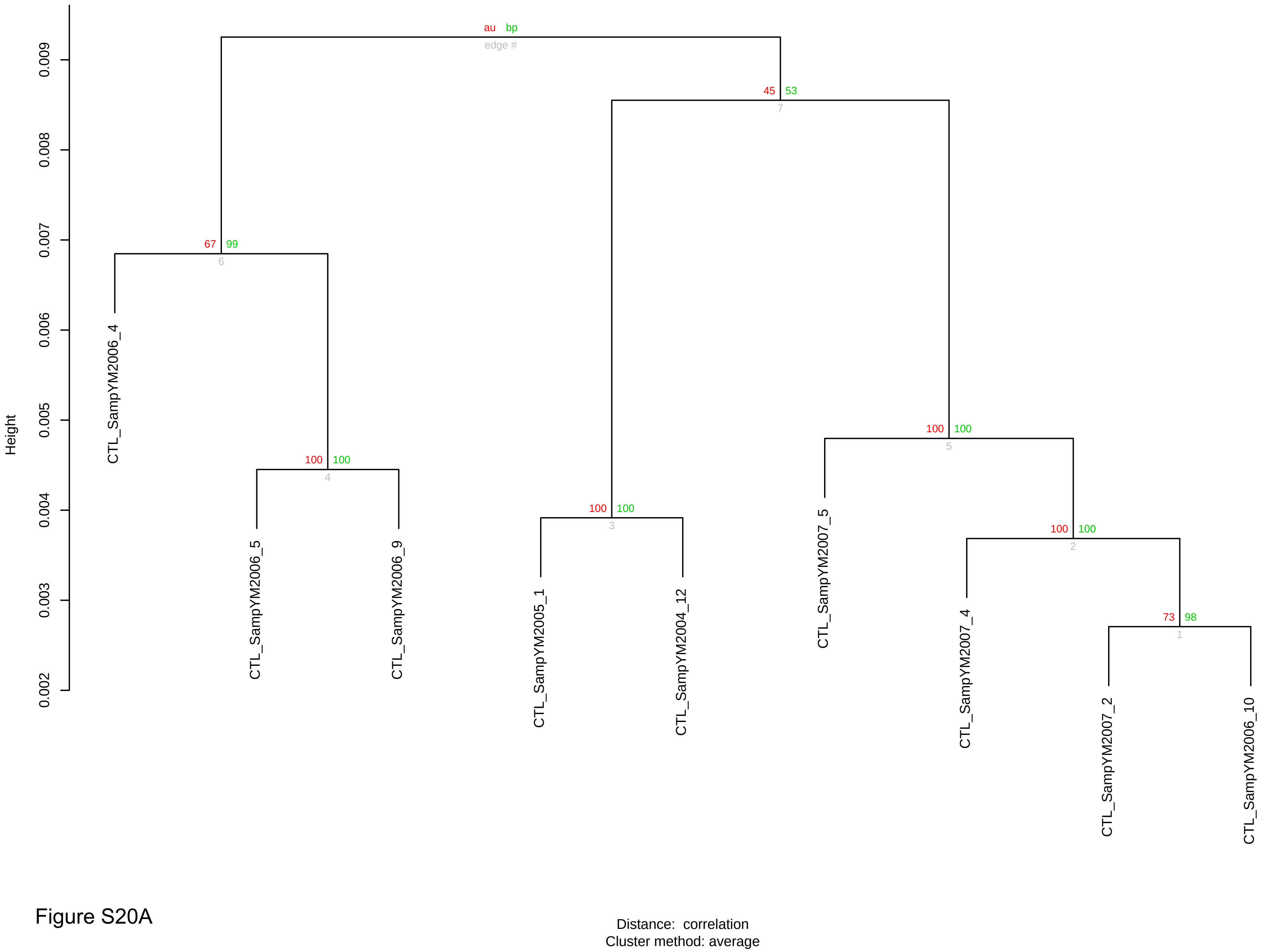

au bp
edge #
45 53
7
67 99
6
100 100
5
100 100
4
100 100
3
100 100
2
73 98
1
Height
0.009
0.008
0.007
0.006
0.005
0.004
0.003
0.002
CTL_SampYM2006_4
CTL_SampYM2006_5
CTL_SampYM2006_9
CTL_SampYM2005_1
CTL_SampYM2004_12
CTL_SampYM2007_5
CTL_SampYM2007_4
CTL_SampYM2007_2
CTL_SampYM2006_10
Distance: correlation
Cluster method: average

Figure S20A

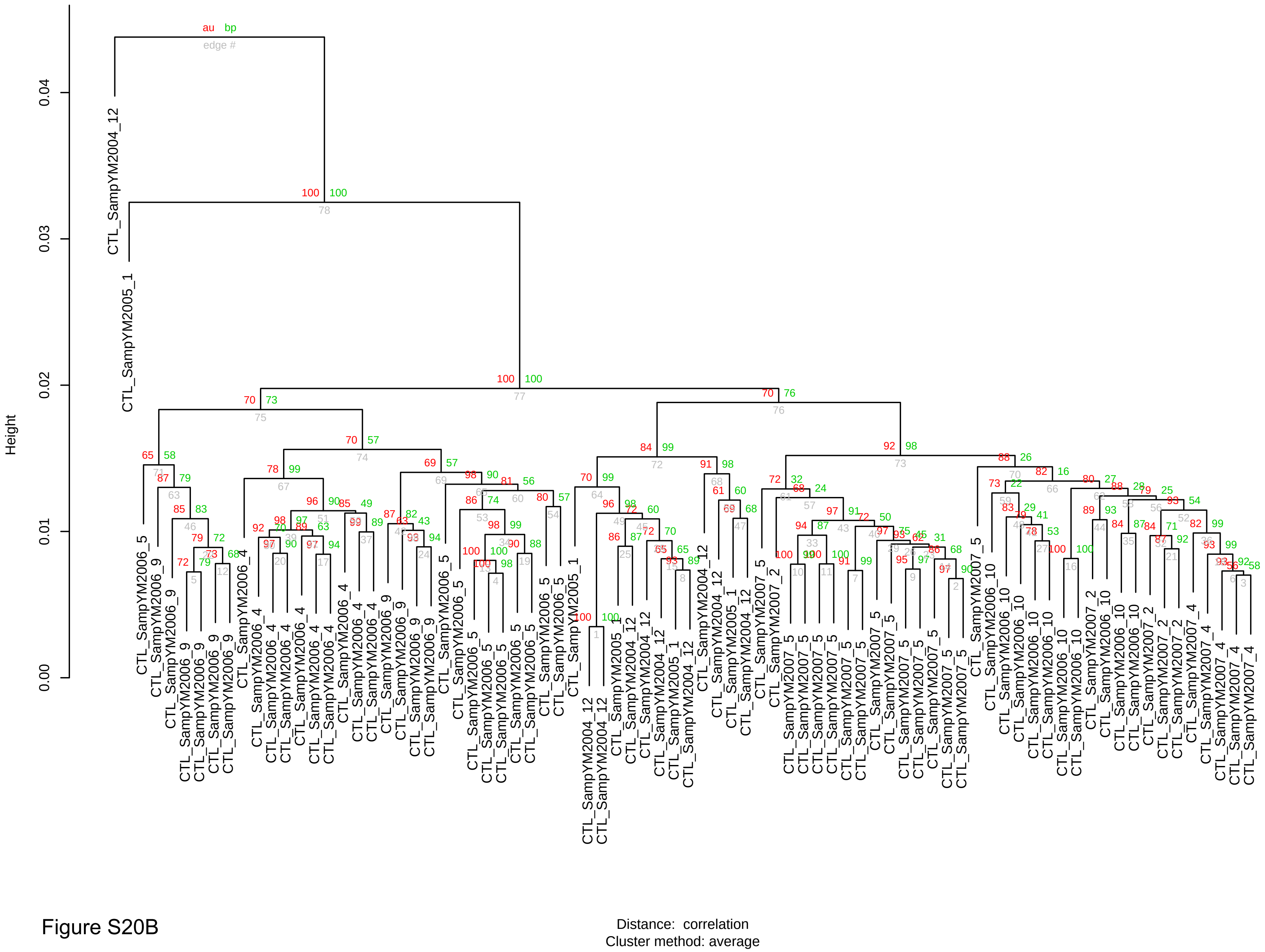

au bp
edge #
Height
0.00
0.01
0.02
0.03
0.04
CTL_SampYM2004_12
CTL_SampYM2005_1
Distance: correlation
Cluster method: average

Figure S20B

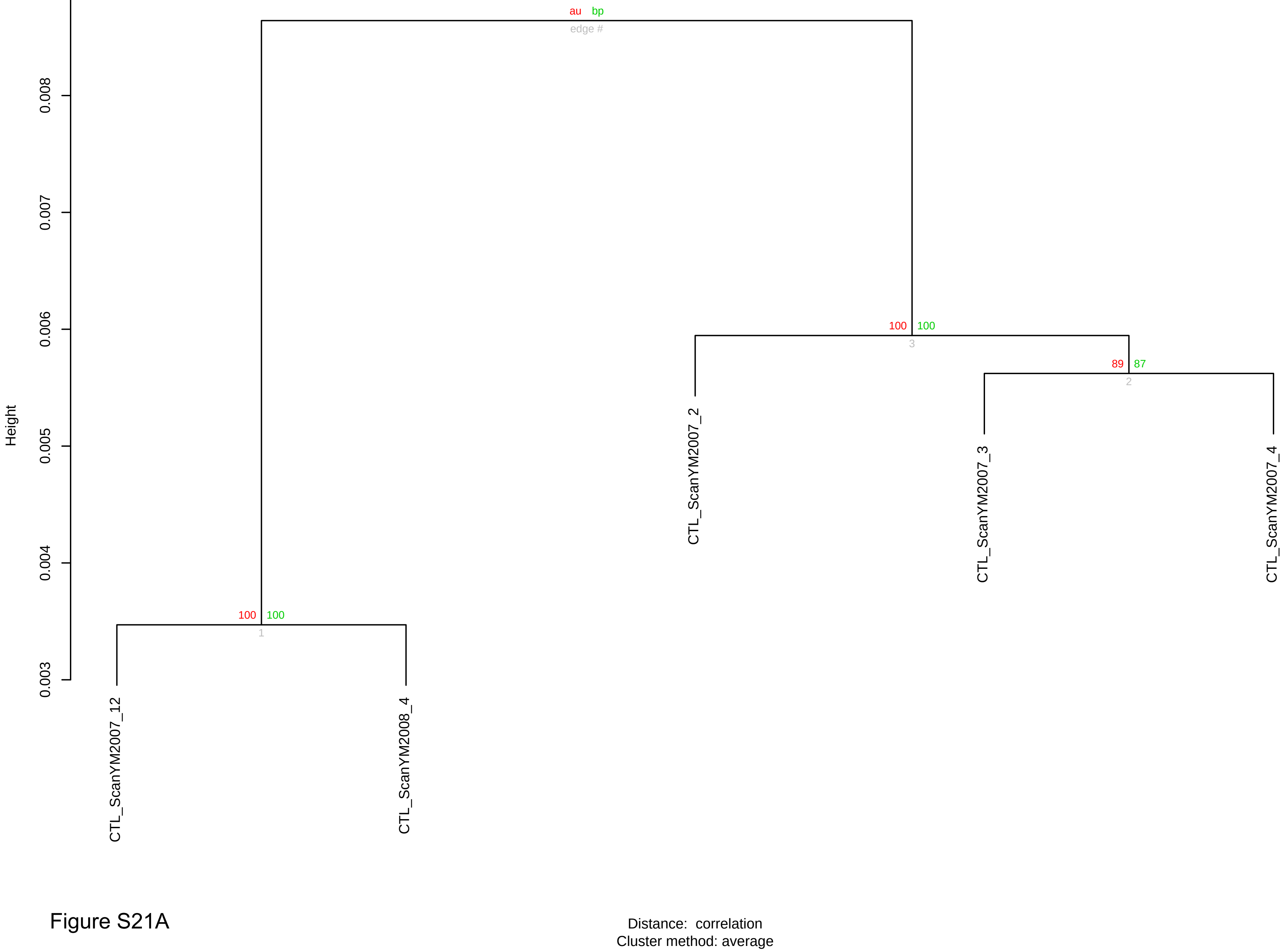
au bp
edge #
Height
0.008
0.007
0.006
0.005
0.004
0.003
100 100
1
100 100
3
89 87
2
CTL_ScanYM2007_12
CTL_ScanYM2008_4
CTL_ScanYM2007_2
CTL_ScanYM2007_3
CTL_ScanYM2007_4
Distance: correlation
Cluster method: average

Figure S21A

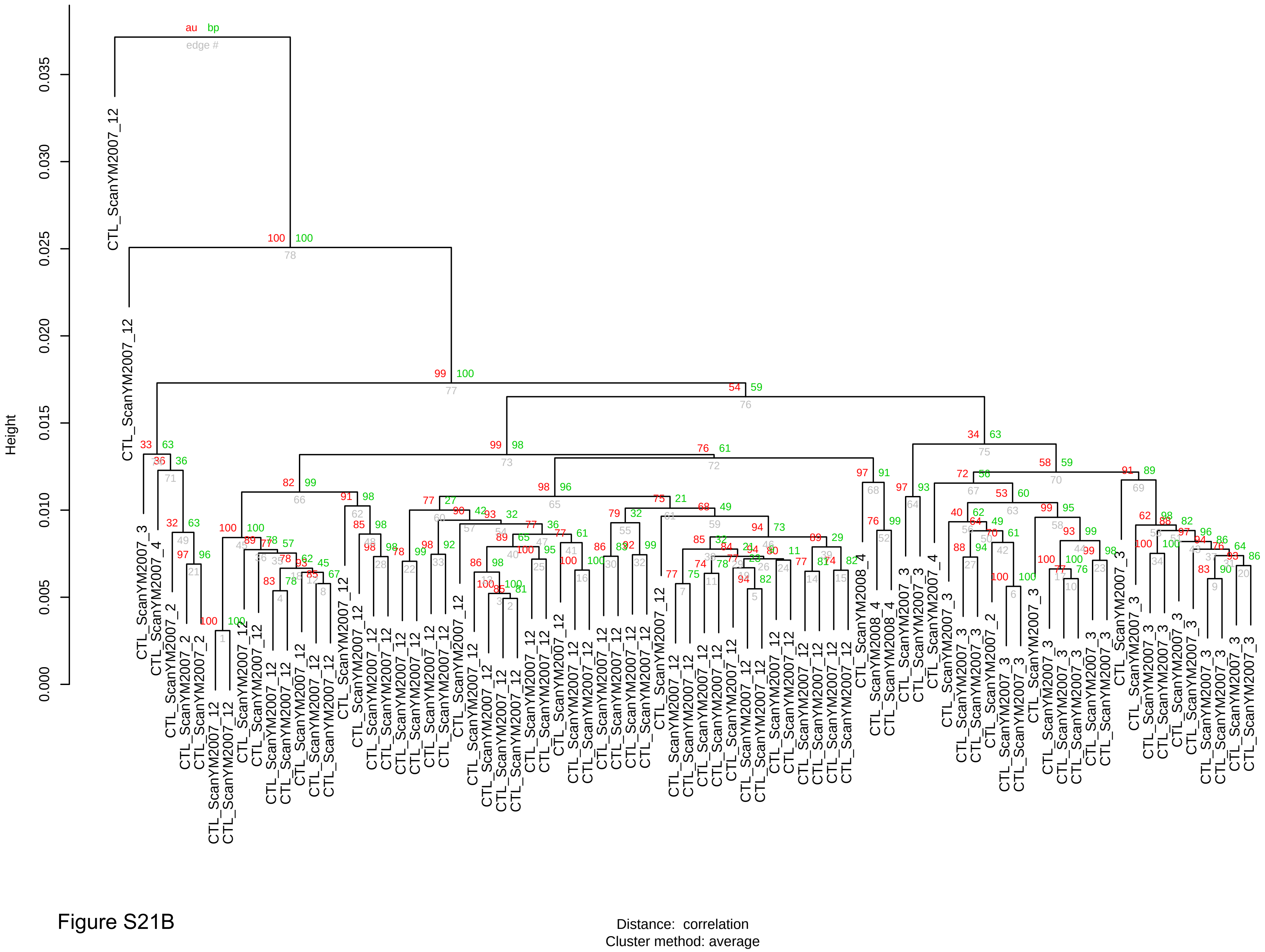
au
bp
edge #
Height
0.000
0.005
0.010
0.015
0.020
0.025
0.030
0.035
CTL_ScanYM2007_12
Distance: correlation
Cluster method: average

Figure S21B
